# Epigenetic Landscapes of Single-Cell Chromatin Accessibility and Transcriptomic Immune Profiles of T Cells in COVID-19 Patients

**DOI:** 10.3389/fimmu.2021.625881

**Published:** 2021-02-24

**Authors:** Shun Li, Bin Wu, Yun Ling, Mingquan Guo, Boyin Qin, Xiaonan Ren, Chao Wang, Hua Yang, Lixiang Chen, Yixin Liao, Yang Liu, Xiuhua Peng, Chunhua Xu, Zhenyan Wang, Yinzhong Shen, Jun Chen, Li Liu, Bowen Niu, Mengmin Zhu, Lingling Liu, Feng Li, Tongyu Zhu, Zhaoqin Zhu, Xiaohui Zhou, Hongzhou Lu

**Affiliations:** ^1^Department of Animal Model, Shanghai Public Health Clinical Center, Fudan University, Shanghai, China; ^2^Department of Infectious Disease, Shanghai Public Health Clinical Center, Fudan University, Shanghai, China; ^3^Department of Diagnosis, Shanghai Public Health Clinical Center, Fudan University, Shanghai, China; ^4^Department of Scientific Research, Shanghai Public Health Clinical Center, Shanghai, China; ^5^Department of Infectious Diseases and Immunology, Shanghai Public Health Clinical Center, Fudan University, Shanghai, China; ^6^Department of Urology, Shanghai Public Health Clinical Center, Shanghai, China

**Keywords:** SARS-CoV-2, COVID-19, chromatin accessibility, transcriptome profiling, scATAC-seq, ScRNA-seq, T cells

## Abstract

T cells play a critical role in coronavirus diseases. How they do so in COVID-19 may be revealed by analyzing the epigenetic chromatin accessibility of cis- and trans-regulatory elements and creating transcriptomic immune profiles. We performed single-cell assay for transposase-accessible chromatin (scATAC) and single-cell RNA (scRNA) sequencing (seq) on the peripheral blood mononuclear cells (PBMCs) of severely ill/critical patients (SCPs) infected with COVID-19, moderate patients (MPs), and healthy volunteer controls (HCs). About 76,570 and 107,862 single cells were used, respectively, for analyzing the characteristics of chromatin accessibility and transcriptomic immune profiles by the application of scATAC-seq (nine cases) and scRNA-seq (15 cases). The scATAC-seq detected 28,535 different peaks in the three groups; among these peaks, 41.6 and 10.7% were located in the promoter and enhancer regions, respectively. Compared to HCs, among the peak-located genes in the total T cells and its subsets, CD4^+^ T and CD8^+^ T cells, from SCPs and MPs were enriched with inflammatory pathways, such as mitogen-activated protein kinase (MAPK) signaling pathway and tumor necrosis factor (TNF) signaling pathway. The motifs of TBX21 were less accessible in the CD4^+^ T cells of SCPs compared with those in MPs. Furthermore, the scRNA-seq showed that the proportion of T cells, especially the CD4^+^ T cells, was decreased in SCPs and MPs compared with those in HCs. Transcriptomic results revealed that histone-related genes, and inflammatory genes, such as NFKBIA, S100A9, and PIK3R1, were highly expressed in the total T cells, CD4^+^ T and CD8^+^ T cells, both in the cases of SCPs and MPs. In the CD4^+^ T cells, decreased T helper-1 (Th1) cells were observed in SCPs and MPs. In the CD8^+^T cells, activation markers, such as CD69 and HLA class II genes (HLA-DRA, HLA-DRB1, and HLA-DRB5), were significantly upregulated in SCPs. An integrated analysis of the data from scATAC-seq and scRNA-seq showed some consistency between the approaches. Cumulatively, we have generated a landscape of chromatin epigenetic status and transcriptomic immune profiles of T cells in patients with COVID-19. This has provided a deeper dissection of the characteristics of the T cells involved at a higher resolution than from previously obtained data merely by the scRNA-seq analysis. Our data led us to suggest that the T-cell inflammatory states accompanied with defective functions in the CD4^+^ T cells of SCPs may be the key factors for determining the pathogenesis of and recovery from COVID-19.

## Introduction

The novel coronavirus, the SARS-CoV-2-caused coronavirus disease (COVID-19), has spread globally (1–4). The clinical symptoms of COVID-19 include fever, pneumonia, dry cough, myalgia, fatigue, diarrhea, and conjunctivitis, while a proportion of patients suffer from acute respiratory distress syndrome (ARDS) and multiple organ failure (5–8). Although much effort is being made by clinicians and scientists to explore antiviral drugs and to produce vaccines, there is yet no clinically approved prophylactic measure or specific cure for COVID-19 (9–12). The status of potential immune mechanisms underlying the pathogenesis of and recovery from COVID-19 requires further investigation.

The adaptive immune system plays an important role in responding to the SARS-CoV-2 infection (13). Our previous study found that CD3^+^ T cells were suppressed in patients with COVID-19 and the loss of CD3^+^ T cells could be an underlying mechanism for the progression of COVID-19 and fatality (14). In addition, blood CD4^+^ and CD8^+^ T-cell counts could provide a promising biomarker for the disease assessment and monitoring of patients with COVID-19 (15). In single-cell resolution, the immune cell profiling of peripheral blood mononuclear cells (PBMCs) and bronchoalveolar immune cells in patients with COVID-19 have been explored by the scRNA-seq (13, 16–23). Zheng et al. (24) reported a single-cell landscape of human circulating immune cell aging and single cell analysis of immune cells in young and aged patients with COVID-19, at the transcriptomic and protein levels. However, the characteristics of chromatin accessibility, cis-regulatory elements, and trans-factors that drive the epigenetic cell states, which are critical for gene transcriptional regulation in the T cells of patients with COVID-19, at moderate and severe or critical stages, are still unclear. Chromium scATAC is a novel technology that can be used to analyze the landscape of chromatin accessibility, providing a deep insight into the cell types and the epigenetic states at the single-cell level (25). Therefore, we combined a single-cell ATAC (scATAC-seq) and RNA sequencing (scRNA-seq) to comprehensively analyze the chromatin accessibility and immune profiling in PBMCs obtained from COVID-19 cases of SCPs and MPs compared with those from HCs.

## Materials and Methods

### Patients

#### Ethical Statement

Ethical approval was obtained from the Research Ethics Committee of Shanghai Publich Health Clinical Center. All participants provided written informed consent for sample collection and subsequent analyses.

#### Subjects and Clinical Sample Collection

Samples from 10 patients with COVID-19 and five health volunteers used in this study were collected from the Shanghai Public Health Clinical Center. All patients were confirmed to be positive for SARS-CoV-2 using PCR with reverse transcription from the swab of an upper respiratory tract (nose and throat) tested at an accredited laboratory. The degree of severity was identified as moderate, severe, or critical infection, according to the recommendations from the WHO. Moderate patients were defined as having fever, respiratory symptoms, and pneumonia as evidenced by CT imaging. Severe infection was defined as one of the following conditions in a patient confirmed to have COVID-19: respiratory distress with a respiratory rate of >30 breaths per minute, blood oxygen saturation of <93%, or arterial oxygen partial pressure/FiO_2_ < 300 mmHg. Critical infection was defined as a respiratory failure requiring mechanical ventilation or shock or other organ failures requiring admission to an intensive care unit. The demographic characteristics of these study populations are provided in Supplementary Table 1. The patients included were five SCPs and five MPs, and five HCs were included as a control group. Detection of SARS-CoV-2 was based on sputum, nasal swab viral PCR assays, clinical symptoms, exposure history, and chest radiography.

The PBMCs were isolated from heparinized venous blood of patients or healthy donors using a Ficoll–Hypaque density solution according to the standard density gradient centrifugation methods. For each sample, the cell viability exceeded 80%. The experimental flow graph for this study is shown in Supplementary Figure 1.

### Processing of scATAC-Seq

Raw sequencing data were converted into the FASTQ format using the cellranger-atac mkfastq (10× Genomics, V.1.2.0). The GRCh38 reference genome was used for data alignment and the cellranger-atac mkfastq was used for generating the FASTQs. For mapping and chromatin accessibility, the cellranger-atac count was used, and the cellranger-atac aggr was used for aggregating the data. The R package harmony (Version: 1.0) was used for removing the batch effects of scATAC (26). All of the quality information of nine samples are shown in the Supplementary Data Sheets 2–10. After filtering, a total of 32,643 cells (11,965/8,487/12,191 cells) for HCs, a total of 29,898 cells (12,776/9,984/7,138 cells) for MPs, and a total of 14,029 cells (5,998/2,264/5,767) for SCPs were left for subsequent analysis.

### Filtering Cells, Calculating Differential Peaks, and Mapping scATAC-Peaks Into Genes

The Signac v0.2.4 was used to filter cells and peaks. The threshold of proportion of readings falling on the peak is not <15%. The lower proportion of cells usually represents low-quality cells or technical errors, which should be deleted. The proportion of the blacklist sequence provided by ENCODE, the filtering threshold, is not higher than 5%. Those thresholds were referenced from the official website of Signac. Nucleosome signal represents the ratio of mononucleosomal/nucleosome-free regions, and the threshold is not higher than 10%. The enrichment of the transcription start site (TSS) is also an important quality control indicator for evaluating Tn5 targeting in the ATAC-seq experiments. A poor ATAC-seq experiment will usually have a lower TSS enrichment score, and the threshold is set to 2%. At the same time, it is necessary to satisfy that each cell must contain one thousandth (0.1%) of the total number of peaks; each peak must cover 2% of the total number of cells (27). The Signac R-package was used for the scATAC-seq data analysis, and the logistic regression model (LR) was applied for statistical testing. In the differences between each group, the *p*-value was ≤0.05, and differences ≥1.5 times were defined as the differential peaks. Regarding scATAC-peaks mapping to genes, the 10× official cellranger-ATAC standard of gene association annotation was used. For the peak annotation, we used the definition of enhancer from the website (http://uswest.ensembl.org/info/genome/funcgen/regulatory_build.html); later, the bedtools were applied for finding the intersection of the peaks and the enhancer region. In addition, the definition of promoter, exon, intron, and intergenic regions were referred from the file (ftp://ftp.ensembl.org/pub/release-84/gtf/homo_sapiens/Homo_sapiens.GRCh38.84.gtf.gz); later, the bedtools were applied for finding the intersection of the peaks and the specific regions.

### Dimensionality Reduction and Clustering for scATAC-seq

The filtered gene-barcode matrix was normalized with the term frequency-inverse document frequency (TF-IDF) normalization. Dimensional reduction was performed by running singular value decomposition (SVD) on the TF-IDF normalized matrix. *t*-Distributed stochastic neighbor embedding (*t*-SNE) was performed on the top 30 principal components for visualizing the cells. Meanwhile, the SLM algorithm was performed for clustering analysis.

### Cell Clustering and Cell Type Identification

We performed scATAC-seq on PBMC from SCPs, MPs, and HCs, taking three cases in each group. The clustering analysis identified 12 distinct clusters composed of T (CD3G), NK (NKG7), B (MS4A1), and monocyte cells (IL-1B) by the signature genes. We further increased the resolution so that T cells were subclustered into CD8^+^ T cells (CD8A) and CD4^+^ T cells.

### R.chromVAR

We measured the activity of TF using chromVAR (V1.8.0). We used the cells by peaks and the Catalog of Inferred Sequence Binding Preferences (CIS-BP) motif (from chromVAR motifs “human_pwms_v1”) matches within those peaks from motifmatchr. We then computed the GC-bias-corrected deviation scores using the chromVAR “deviation Scores” function.

### Aggregate Read Counts in 4-kb Windows Centered on Each Identified Motif Instance

Fimo v5.1.1 was used to scan the peak sequence to match the known motif (JASPAR2016_CORE). Then, the R package was used to calculate the mean readings per million distributions on each motif.

### Co-accessibility

The GRCh38 was used as the reference database. We calculated the co-accessibility between all the peaks for specific regions by using the R package Cicero 14 and the connecting lines were drawn.

### The Heatmap of Gene Ontology (GO) and Kyoto Encyclopedia of Genes and Genomes (KEGG) Enrichments for the Total T Cells, CD4^+^, and CD8^+^T Cells

Each cell types (total T cells, CD4^+^, and CD8^+^ T cells) were pooled, and peaks with an increase and decrease in accessibility from each compared groups (HCs vs. MPs, MPs vs. SCPs, and HCs vs. SCPs) were identified. The GO enrichment and the KEGG pathways were analyzed by using identified peak-related genes, and the significant GO terms and KEGG pathways were identified. Then, the level of accessibility was visualized for each hit peak (row) in the GO term and the KEGG pathway, and the *z*-score normalization was performed across patients.

### Capturing Library Construction and Sequencing for scRNA

The single-cell suspensions of scRNA-seq samples were converted to barcoded scRNA-seq libraries using the Chromium Single Cell 3′ Library, Gel Bead and Multiplex Kit, and Chip Kit (10× Genomics). The Chromium Single Cell 3′ v3 Reagent (10× Genomics, 1000078) kit was used to prepare single-cell RNA libraries according to the manufacturer's instructions. The FastQC software was used for quality check (21). The Cell Ranger software (version 3.0.1) was used for the initial processing of the sequencing data. The Seurat (Version 3.1.2) was applied for removing the batch effects of scRNA-seq.

### The scRNA-seq Data Alignment and Sample Aggregating

We demultipled and barcoded the samples by using The Cell Ranger Software Suite (cellranger-3.0.1) (https://support.10xgenomics.com/). Cellranger-atac mkfastq was used for demultiplexing, and the cellranger count was used for the alignment, de-duplication, filtering, and generating feature-barcode matrices. After getting gene counts from each sample, we aggregate them together. Finally, the gene-barcode matrix of all ten patients and five HCs were integrated with the cellranger-atac aggr.

Seurat v3.1.2 (https://satijalab.org/) was used, and several criteria were applied to each cell, that is, filtering the cells with gene numbers <200, filtering the cells with the unique molecular identifier (UMI) ratio of the MT genes that are ≥15%, filtering the UMI ratio of red blood cells (HBB, HBA1, and HBA2) that are ≥10%, and removing the potential double cells by scrublet. After filtering, a total of 39,233 cells (8,264/8,932/7,297/10,375/4,365 cells) for HCs, a total of 34,702 cells (5,994/6,198/7,165/6,385/8,960 cells) for severe or critical cases, and a total of 33,927 cells (7,071/7,531/2,997/8,105/8,223 cells) for moderate cases were left for subsequent analysis. The UMI count matrix was converted to Seurat objects using the R package Seurat v3.1.2.

### Dimensionality Reduction and Clustering for scRNA-seq

The filtered gene-barcode matrix was normalized with the LogNormalize methods in Seurat and analyzed by the principal component analysis (PCA) using the top 2, 000 most variable genes. Then, *t*-SNE) was performed on the top 30 principal components for visualizing the cells. Meanwhile, a graph-based clustering was performed on the PCA-reduced data for clustering analysis with Seurat v3.1.2.

### Cell Clustering and Cell Type Identification

We performed scRNA-seq on PBMC from five patients with severe/critical infection (severe/critical cases 1–5, SCPs), five patients with moderate COVID-19 (moderate cases 1–5, MPs), and five healthy controls (health volunteers 1–5, HCs). The clustering analysis showed 38 distinct clusters composed of myeloid cells (CD14, CD1C, and FCGR3A), T cells (CD3E), natural killer (NK) cells (NCAM1), and B cells (MS4A1), which were identified by signature genes. We re-clustered the subclusters of the T cells to further dissect their heterogeneity. According to the recently published classification criteria and typical markers, the T cells were categorized into CD4^+^ T cells (CD3E and CD4) and CD8^+^ T cells (CD3E and CD8). The CD4^+^ T cells were subdivided into four clusters, namely the naïve CD4^+^ T cells, which expressed high levels of CCR7, LEF1, and TCF7; the central memory CD4^+^T cells, which expressed high levels of CCR7 but more AQP3 and CD69 compared to naïve CD4^+^ T cells; the effector memory CD4^+^ T cells, which expressed high levels of CCR6 and CXCR6; and the regulatory T (Treg) cells, which expressed FOXP3. In addition, the CD4^+^ T cells were subidentified into T helper-1 (Th1) (expressed tbx21), Th17 (expressed RORC), and Treg cells (highly expressed FOXP3). The CD8^+^ T cells were subdivided into three clusters, namely the naïve CD8^+^ T cells, which expressed high levels of CCR7 and LEF1, similar to naïve CD4^+^ T cells; the effector memory CD8^+^ T cells, which expressed high levels of GZMK; and the cytotoxic CD8^+^ T cells, which expressed high levels of GZMB. The TYMS^+^ MKI67^+^ cells were proliferating T cells. The top 30 DEG marker genes of each cluster are shown by heatmaps.

### Differential Analysis for Cell Types

Findmarker in Seurat v3.1.2 was used to perform differential analysis and the bimod likelihood ratio statistical test was used.

### Regulatory Network Inference

The single-cell regulatory network was constructed with the single-cell regulatory network inference and clustering (SCENIC) (28). Specifically, GRNBoost2 (https://github.com/tmoerman/arboreto) in pySCENIC was applied to infer gene regulatory networks from the expression data. A heatmap from the regulator's group was generated with the R package AUCell.

### Gene Functional Annotation

For differentially expressed genes (DEGs), GO was analyzed by using topGO (http://www.bioconductor.org/packages/release/bioc/html/topGO.html) and the website (http://www.genome.jp/kegg-bin/show_organism?menu_type=pathway_maps&org=hsa) was used for analyzing the KEGG pathway.

### Raw Data Deposition

The raw data reported in this paper have been submitted in the China National GeneBank DataBase (https://db.cngb.org/) and the submission number is CNP0001507.

### Integration Analysis of scRNA and scATAC Data

MAESTRO (Release V1.3.0) was used for the integration analysis of scRNA and scATAC data, and the methods were referenced from the published paper (29).

## Results

### Epigenetic Landscapes of Single-Cell Chromatin Accessibility of T Cells From Patients With COVID-19

**Figure 1:**
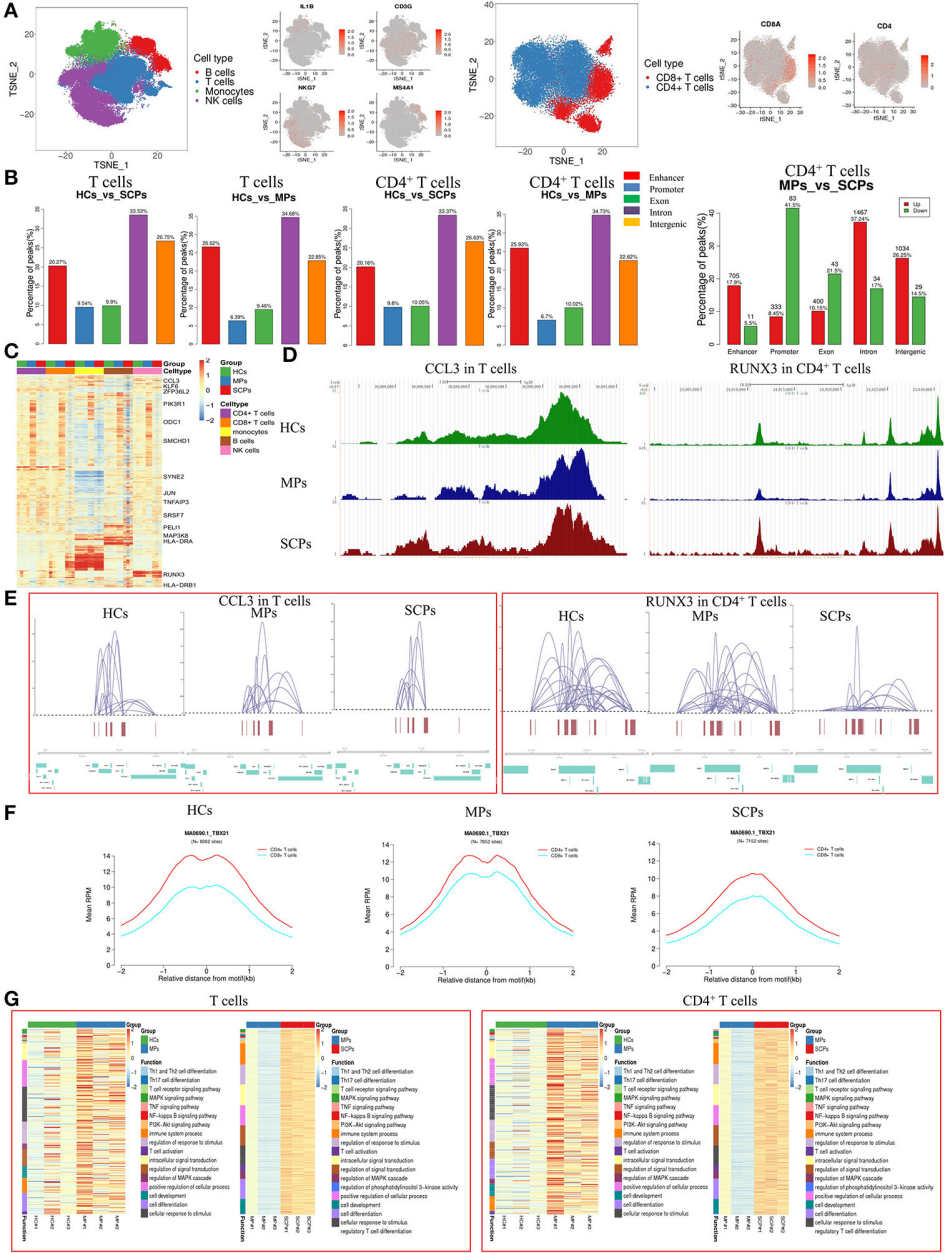
Single-cell landscape of T cells in patients with COVID-19 by scATAC-seq. **(A)** The *t*-distributed stochastic neighbor embedding (*t*-SNE plot shows a comparison of the clustering distribution of B, T, monocyte, and natural killer (NK) cells in the peripherial blood mononuclear cells (PBMC) of patients with COVID-19 through a single-cell assay for transposase-accessible chromatin (ATAC). The T cells were reclustered into CD4^+^T cells and CD8^+^ T cells with *t*-SNE projection of canonical markers, including MS4A1 for B cells, CD3G for T cells, IL-1B for monocytes, and NKG7 for NK cells, CD8^+^T cell marker (CD8A), and CD4^+^ T cell marker (CD4). **(B)** Distribution of different peaks in total T and CD4^+^ T cells from severely ill/critical patients (SCPs) vs. healthy volunteer controls (HCs) and moderate patients (MPs) vs. HCs. The upregulated and downregulated differential peaks in CD4^+^ T cells from MPs vs. SCPs are shown on the right. Five genomic features were studied, including enhancer, promoter, exon, intron, and intergenic elements. **(C)** Heatmaps of *z*-scores of 14,921 cis-regulatory elements in CD4^+^, CD8^+^, monocytes, B, and NK cells by scATAC-seq; representative genes are labeled. **(D)** Single-cell ATAC-seq genome tracks for the CCL3 loci in total T cells and RUNX3 loci in CD4^+^ T cells among HCs, MPs, and SCPs. **(E)** The reconstruction of genome-wide cis-regulatory interaction networks for representative regions across specific genes for total T and CD4^+^ T cells among three groups. **(F)** Aggregate read counts in 4-kb windows centered on identified motif instance is shown for CD4^+^ and CD8^+^ T cells in HCs, MPs, and SCPs. Blue indicates CD8^+^ T cells and red indicates CD4^+^ T cells. **(G)** The heatmaps of Gene Ontology (GO) and Kyoto Encyclopedia of Genes and Genomes (KEGG) enrichments for the total T and CD4^+^ T cells in the group of HCs vs. MPs and MPs vs. SCPs revealed by scATAC-seq.

In an epigenetic analysis based on scATAC-seq, we analyzed the PBMC from SCPs, MPs, and HCs, taking three cases from each group. The clustering analysis identified 12 distinct clusters composed of T (CD3G), NK (NKG7), B (MS4A1), and monocyte cells (IL1B) by those signature genes (30) (Figure 1A). We further increased the resolution so that T cells were subclustered into CD8^+^ T cells and CD4^+^ T cells (Figure 1A and Supplementary Figures 2A,B). We checked the distribution of genomic features for different peaks from all cell types, the total T cells, and its subsets, CD4^+^ T and CD8^+^ T cells, among the three groups of SCPs, MPs, and HCs. Five genomic distribution features, including enhancer, promoter, exon, intron, and intergenic elements, were investigated. We detected 28,535 different peaks across all cells, of which 41.6 and 10.7% were located in the promoter and enhancer regions, respectively (Supplementary Figure 3A). We also calculated the percentage of those different peaks in total T cells and its subsets, CD4^+^ T cells and CD8^+^ T cells, and found that the different peaks were mainly located in the region of intron, and also in the promoter and enhancer regions (Figure 1B and Supplementary Figures 3A–D). In CD4^+^ T cells, the proportions of accessible cis-regulatory elements (ATAC-seq peaks are located either in the enhancer or in the promoter region) were decreased from 32.63% (HCs vs. MPs, 25.93% in the enhancer region and 6.7% in the promoter region) to 29.96% (HCs vs. SCPs, 20.16% in the enhancer region and 9.8% in the promoter region). In the CD8^+^ T cells, the proportion was increased from 30.51% (HCs vs. MPs, 20.75% in the enhancer region and 9.76% in the promoter region) to 31.58% (HCs vs. SCPs, 18.42% in the enhancer region, and 13.16% in the promoter region). After comparing MPs with SCPs in CD4^+^ T cells, the downregulated peaks were located mainly in the regions of promoter and exon, and the upregulated peaks were located in the enhancer, intron, and intergenic regions (Figure 1B). The different peaks located in the promoter regions of CD4^+^ T cells were increased from 6.7% (HCs vs. MPs) to 9.8% (HCs vs. SCPs). In total, we identified 14,921 cis-regulatory elements across all cell types, and a heatmap of cis-regulatory elements indicates the accessibility in cell types from the three different groups (Figure 1C). Genome tracks facilitated a comparison of aggregate single-cell accessibility profiles of individual genomic loci. For example, the accessibility profiles of CCL3, which is involved in the chemotaxis of inflammatory cells, and of RUNX3, which is involved in the positive regulation of cellular process, and the response to stimulus and regulation of the biological process were compared among total T cells and its subsets, CD4^+^ T and CD8^+^ T cells, in SCPs, MPs, and HCs (Figure 1D and Supplementary Figures 4A–J).

The cis-regulatory interaction networks of specific regions for the total T cells and its subsets, CD4^+^ T, and CD8^+^ T cells, among the three groups were constructed from this data. The results showed that some loci (e.g., CCL3) in T cells had similar interactive networks with other genes among the three groups. However, some genes (e.g., RUNX3 in CD4^+^ T cells) were less interactive in SCPs compared to MPs (Figure 1E and Supplementary Figures 5A–L). The chromatin accessibility profiles of the selected motifs were also analyzed. Different motifs differed in accessibility in the total T cells and its subsets, CD4^+^ T and CD8^+^ T cells, varied among the three groups. Compared to MPs, the motifs of TBX21, NFKB1, TP53, STAT1, MAFK, and RUNX3 were less accessible in the CD4^+^ T and CD8^+^ T cells of SCPs, but much less in the CD4^+^ T cells. Since T-bet (encoded by TBX21 gene) plays a key role in Th1 commitment, the altered accessibility status of TBX21 in the CD4^+^ T cells indicated a possible decreased Th1 function in SCPs compared to that in MPs (Figure 1F and Supplementary Figures 6A–D).

We analyzed the different peaks and their associated genes for the total T cells and its subsets, CD4^+^ T cells and CD8^+^ T cells, comparing HCs vs. MPs, MPs vs. SCPs, and HCs vs. SCPs. Then, the GO analysis and KEGG pathways were used to further investigate the relevance of the differential peak-related genes. For total T cells, the GO analysis revealed that the genes involved in the immune system processes, cell activation, the immune response, regulation of the responses to stimuli, regulation of signal transduction, T cell activation, etc. were upregulated in SCPs compared to those in HCs. The regulation of the immune system process, regulation of the response to stimulus, and regulation of signaling, etc. were downregulated in SCPs compared to those in HCs. In the group of MPs, the genes related to the MAPK cascade and to a positive regulation of MAPK cascade [echoing a previous report (13)] were peak-enriched compared with those in HCs. The KEGG analysis comparing SCPs with HCs revealed that the peak-enriched genes were clustered in pathways, such as the MAPK signaling pathway, TNF signaling pathway, NF-kappa B signaling pathway, and PI3K-Akt signaling pathway (Figure 1G and Supplementary Figures 7A–C). The GO analysis of CD4^+^ T cells done by comparing SCPs to HCs revealed that the immune system processes, cell activation, regulation of the response to stimulus, regulation of intracellular signal transduction, etc. were peak-enriched with SCPs. The regulation of the immune system process, positive regulation of the immune system process, and the regulation of signaling, etc. were downregulated in SCPs compared to that of HCs. The genes related to cell communication, regulation of transport, positive regulation of signaling, and cell surface receptor signaling pathways, etc. were peak-enriched in MPs compared to those in HCs. The KEGG analysis revealed that the peak-enriched genes gathered in SCPs compared with HCs in the following pathways, such as Th1 and Th2 cell differentiation, Th17 cell differentiation, T cell receptor signaling pathway, MAPK signaling pathway, and TNF signaling pathway. Although the peak-enriched genes in the groups of MPs vs. HCs are gathered in the same pathway as that in the group of SCPs vs. HCs, the genes involved in these pathways were much fewer than in the group of HCs vs. SCPs. Only three genes (DLL3, MAPK11, JAK3) were involved in the Th1 and Th2 cell differentiation and four genes (TNFRSF1A, MAPK11, CEBPB, and MAP3K14) were related to the TNF-signaling pathway in the groups of HCs vs. MPs (Figure 1G and Supplementary Figures 7D–F). In CD8^+^ T cells, the GO analysis revealed that the genes related to the immune system processes, cell activation, immune response, regulation of the response to stimulus, regulation of cell death, etc. were upregulated in SCPs compared to those in HCs, whereas regulation of the immune system processes, positive regulation of the immune system process and regulation of signaling, etc. were downregulated in SCPs compared to those in HCs. The genes related to cell communication, regulation of the cellular process, positive regulation of response to stimulus, etc. were peak-enriched in CD8^+^ T cells from MPs compared with those from HCs. The KEGG analysis revealed that the peak-enriched genes gathered in the T-cell receptor signaling pathway, the MAPK signaling pathway, and the TNF signaling pathway in CD8^+^ T cells from SCPs compared with those from HCs (Supplementary Figures 7G–K). In short, the scATAC-seq analysis were indicated an activated and inflammatory state of T cells (especially, CD8^+^ T cells) during COVID-19, associated with a possibility of the decreased function of CD4^+^ T cells (i.e., Th1) in the cases of SCPs.

### Transcriptomic Immune Profiles of T Cells From Patients With COVID-19

**Figure 2:**
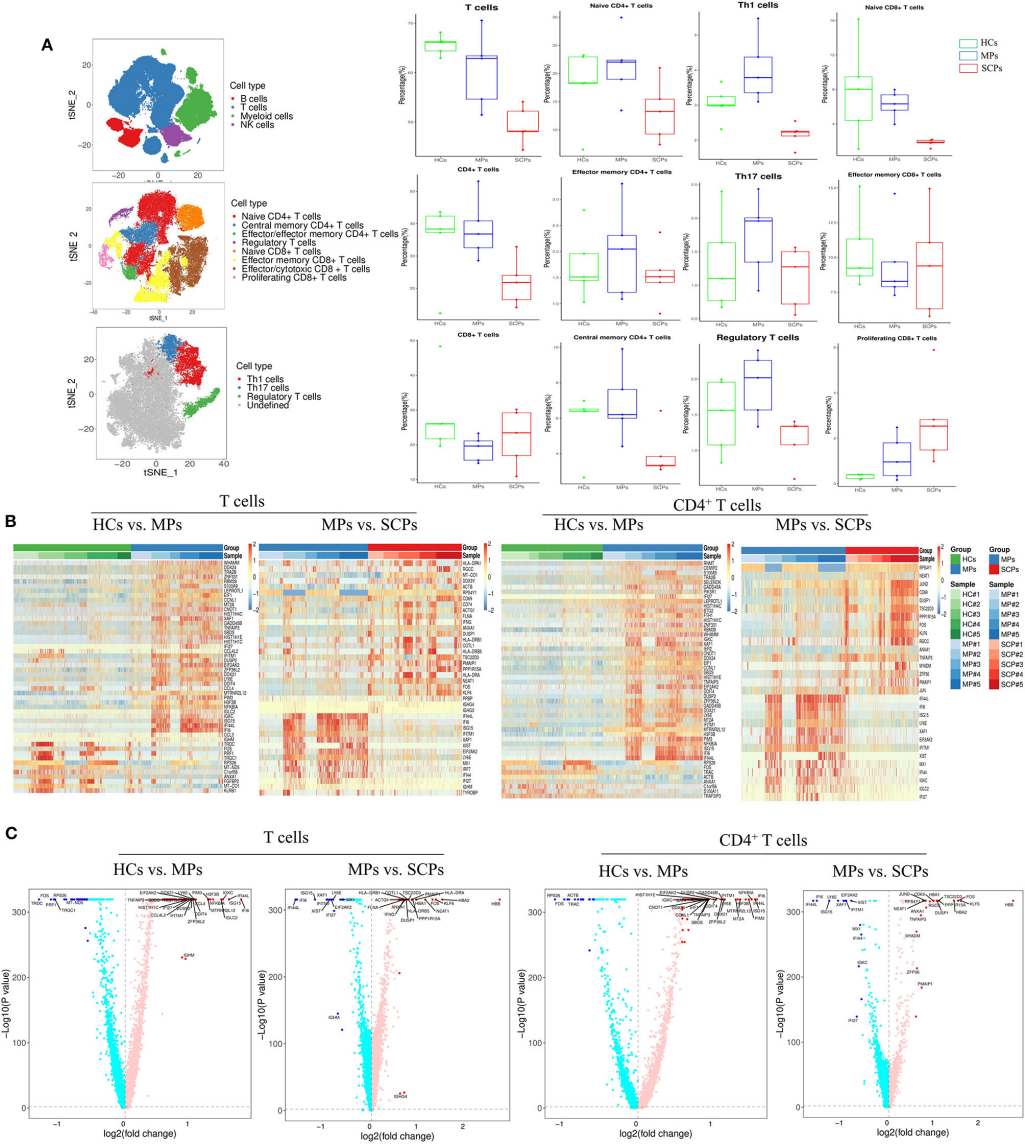
Single-cell transcriptomic immune profiling of T cells in patients with COVID-19 by single cell RNA sequencing (scRNA-seq). **(A)** The *t*-SNE plot shows a comparison of the clustering distribution of PBMCs, the clustering of T cells, and the clustering of T helper-1 (Th1), Th17, and regulatory T (Treg) cells. The boxplots show the proportions of major cell types and subtypes of CD4^+^ and CD8^+^ T cells among HCs, MPs, and SCPs (using two-sided Student's *t*-test for pairwise comparisons). **(B)** The heatmaps show the top differentially expressed genes (DEGs) in the group of HCs vs. MPs and MPs vs. SCPs among the total T and CD4^+^ T cells. **(C)** Volcano plots show a fold change in the group of HCs vs. MPs and MPs vs. SCPs among the total T and CD4^+^ T cells. Findmarker in Seurat v3.1.2 was used to perform differential analysis and a bimod likelihood ratio statistical test was used. Significantly, differential expression genes were defined by both *p*-value (≤0.05) and fold change (≥1.5).

In addition to the epigenetic analysis based on scATAC-seq, we also applied ascRNA-seq to profile the immune transcriptomics of T cells in patients with COVID-19. We performed the scRNA-seq (3′ transcriptome gene expression) on PBMC from SCPs, MPs, and HCs taking five cases from each group, in which three cases were also applied to the scATAC-seq study described above. Our scRNA-seq showed a decreased proportion of T and NK cells and an increased proportion of myeloid cells in SCPs and MPs compared with those from HCs (Figure 2A and Supplementary Figures 8A–D). The enhanced resolution showed that the subsets of T cells and the reduction of CD4^+^T cells could account for the major decrease in the T-cell proportions in SCPs (Figure 2A and Supplementary Figures 8E–H). Those data were consistent with the previous reports of COVID-19-related lymphopenia, especially in the severe and critical cases (14, 15). In the subsets of CD4^+^ T cells, the Th1 cells and the Treg cells were decreased in SCPs (Figure 2A). In the subsets of CD8^+^ T cells, the proportion of naive CD8^+^ T cells was decreased in MPs and SCPs, and the proliferating CD8^+^ T cells have an increasing trend in MPs and SCPs compared to HCs. SCPs showed a slightly higher proportion in effector/memory CD8^+^ T cells compared with those in HCs (Figure 2A).

We analyzed DEG in the total T cells and in its subsets, CD4^+^ T cells and CD8^+^ T cells, by comparing the groups of HCs vs. MPs, MPs vs. SCPs, and HCs. vs. SCPs. Heatmaps and volcano plots show the DEGs profiles among each group of T cells (Figures 2B,C and Supplementary Figure 9A). Upregulated or downregulated genes in terms of the GO analysis and KEGG pathways were analyzed. For total T cells, the GO analysis revealed that the genes associated to the innate immune response, defense response to virus, type-I interferon signaling pathway, and response to type-I interferon were upregulated in MPs compared with HCs. There were only 30 upregulated genes comparing MPs and SCPs, and the genes were mainly associated with cell activation and the immune system process. The genes involved in the immune system processes, regulation of cell death, cell activation, immune response, etc. were upregulated in SCPs compared to HCs, echoing the previous report (31). Histone-related genes (HIST1H1D, HIST1H4C, HIST1H1E, HIST1H1C) and inflammatory genes (NFKBIA, TNFAIP3, PIK3R1, S100A9, etc.) were upregulated both in SCPs and MPs compared with HCs. We further found that those inflammatory genes (NFKBIA, TNFAIP3, PIK3R1, S100A9, S100A8, etc.) were gradually enhanced from HCs to MPs and finally to SCPs in the total T cells. Among the total T cells from SCPs, the T-cell activation marker, CD69, and HLA class II genes (HLA-DRA, HLA-DRB1, and HLA-DRB5) were upregulated. The KEGG analysis revealed that the upregulated genes were mainly enriched with the IL-17 signaling pathway and in the TNF signaling pathway, both in SCPs vs. HCs and MPs vs. HCs (Supplementary Figures 10A–C). To uncover the transcriptional signatures in CD4^+^ T cells, the DEGs of all groups are displayed as heatmaps and volcano plots (Figures 2B,C and Supplementary Figure 9B). The genes associated with innate immune responses, RNA metabolic processes, immune system processes, etc. were upregulated in MPs compared with those in HCs. We also found that the histone-related genes and inflammatory genes that were mentioned earlier in the context of total T cells were upregulated both in SCPs and MPs. In addition, as in the total T cells, those inflammatory genes (NFKBIA, TNFAIP3, PIK3R1, S100A9, etc.) were also gradually enhanced from HCs to MPs and finally to SCPs among CD4^+^ T cells. The KEGG analysis revealed that the upregulated genes were enriched with the IL-17 signaling and TNF signaling pathways in the CD4^+^ T cells collected from both SCPs and MPs vs. HCs (Supplementary Figures 10D–F). Regarding the TBX21 expression level in CD4^+^ T cells, MPs (mean counts = 0.03799) showed similar level as HCs (mean counts = 0.03576). However, the TBX21 expression decreased in SCPs (mean counts = 0.02542) compared to HCs (mean counts = 0.03576). The IFNG and the TBX21 downstream target gene showed repression both in MPs (mean counts = 0.02124) and SCPs (mean counts = 0.03415) compared to HCs (mean counts = 0.04259). To illustrate the transcriptional signatures of CD8^+^ T cells, the DEGs of the groups are shown by heatmaps and volcano plots (Figures 2B,C and Supplementary Figures 9C–E). In CD8^+^ T cells of MPs, the genes related to innate immune responses, responses to viruses, etc. were upregulated in comparison to HCs. Comparing the overlapping of upregulated genes of CD8^+^ T cells with HCs vs. SCPs and HCs vs. MPs, we found histone-related genes, such as HIST1H1D, HIST1H4C, HIST1H1E and HIST1H1C, inflammatory genes, such as NFKBIA, PIK3R1, MAP3K8, S100A8, and S100A9, and chemotactic genes, such as CCL3 and CCL4 in both SCPs and MPs groups. The KEGG analysis revealed that the upregulated genes were enriched with the following pathways, such as the pathways of apoptosis, TNF signaling pathway, and NF-kappa B signaling (Supplementary Figures 10G–I), in SCPs and MPs, compared with HCs. In brief, the scRNA-seq followed by the immune transcriptomic analysis indicated an activation state (through CD69 and HLA class II genes), chemotaxis (e.g., CCL3 and CCL4), an inflammatory state (e.g., NFKBIA, PIK3R1, S100A9, etc.) in T cells, especially in CD8^+^T cells, in COVID-19 cases. Additionally, CD4^+^ T cells showed signs of decreased quantity and function (e.g., anti-viral Th1 and inflammation-suppressive Treg cells) in the cases of SCPs.

### Integrated Analysis of the scATAC-seq and scRNA-seq

**Figure 3:**
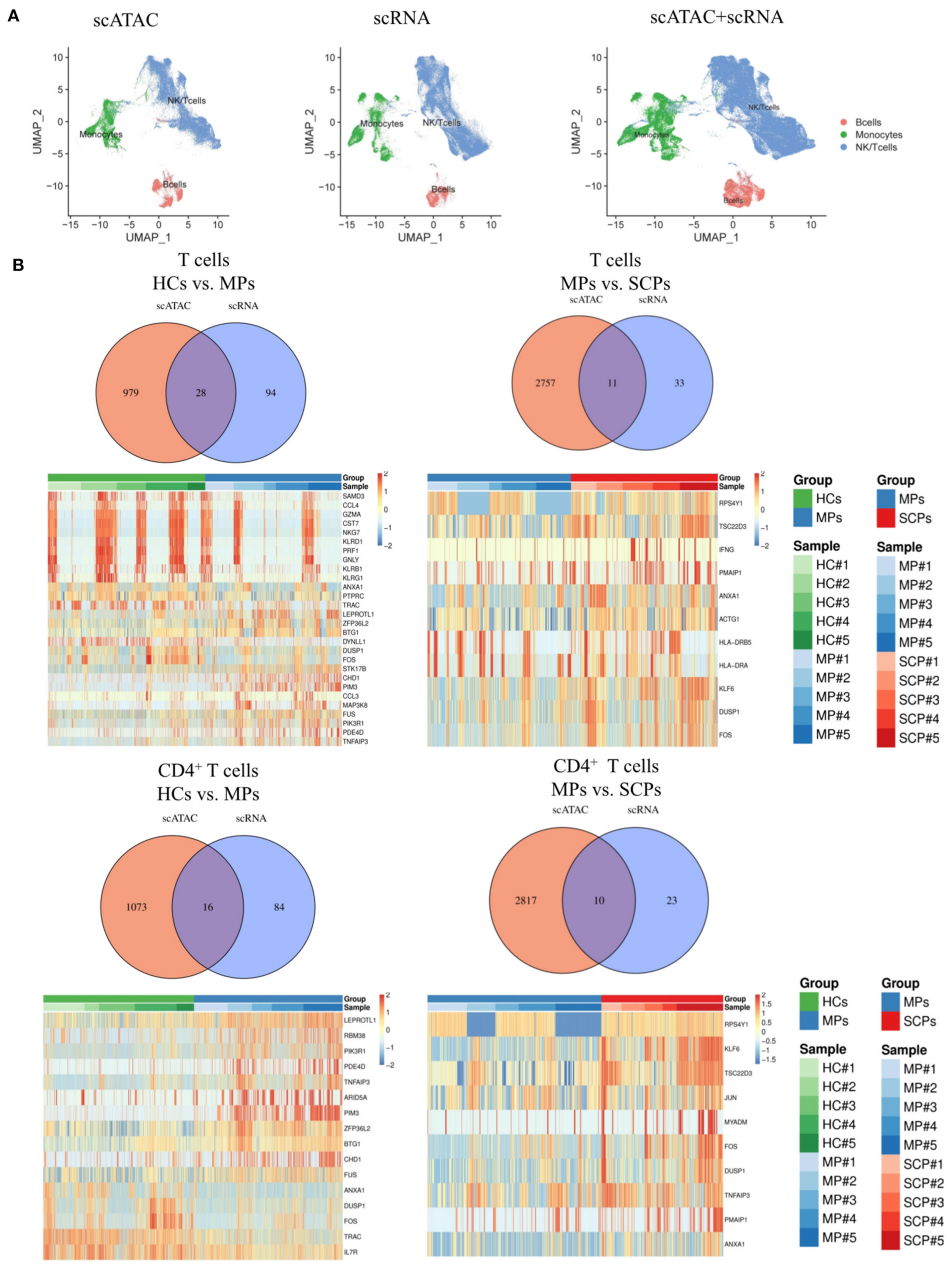
Integrated analysis of scATAC-seq and scRNA-seq. **(A)** The integrated analysis of scATAC-seq and scRNA-seq data shows scATAC (left), scRNA (middle), and merged scATAC and scRNA by the UMAP plot using MAESTRO. **(B)** Venn diagram of all overlapping differential genes for scATAC-seq and scRNA-seq in total T cells and CD4^+^ T cells from the group of HCs vs. MPs and MPs vs. SCPs. The heatmaps of all the overlapping differential genes in the total T cells and CD4^+^ T cells from the group of HCs vs. MPs and MPs vs. SCPs.

We suggest that our data indicated some pathway consistencies between scATAC-seq data and scRNA-seq data, though they came from different levels of the transcription process. Indeed, both scATAC-seq and scRNA-seq KEGG analyses revealed the TNF signaling pathway to be involved in COVID-19; the scATAC-seq data showing lower TBX21 locus accessibility were consistent with the scRNA-seq data, showing decrease in Th1 cells of SCPs compared to the MCPs and HCs. From the integrated analysis ofscATAC-seq and scRNA-seq data, the UMAP plots showed that B cells, monocytes, and NK/T cells were clustered in both scATAC-seq and scRNA-seq and in correlation of scATAC and scRNA to some degree (Figure 3A). The Venn diagrams and heatmaps show all the overlapping differential genes between scATAC and scRNA in T cells and its subsets, CD4^+^ and CD8^+^T cells, from the group of HCs vs. MPs, MPs vs. SCPs, and SCPs vs. HCs (Figure 3B and Supplementary Figures 11A–E). The altered expression of T-cell activation marker (CD69) and inflammatory genes (S100A9, S100A8, PIK3R1, NFKBIA, and TNFAIP3) were also detected in T cells by the integrated analysis of the two sequencing systems. Among the total T and CD4^+^ T cells, the scRNA data analysis showed that the expression of CD69 were gradually increased from HCs to MPs and finally to SCPs. The scATAC data showed that the peaks related to gene CD69 were located in the promoter and distal regions. Compared with HCs, the chromatin accessibility in the promoter region of CD69 was decreased in MPs and slightly lower in SCPs. Among the genes in the TNF signaling pathway (NFKBIA, TNFAIP3, JUNB, MAP3K8), the expression of those genes were increased in MPs and SCPs compared to HCs. The scATAC analysis showed that the chromatin accessibility in the promoter region of JUNB was gradually decreased from HCs to MPs and finally to SCPs. The chromatin accessibility in the promoter region of the genes (NFKBIA, TNFAIP3, and MAP3K8) was similar among the group of HCs, MPs, and SCPs (Supplementary Tables 3–13).

## Discussion

The immune response elicited by virus infection is one of the main ways against the pathogenesis of diseases. Innate and acquired immune responses are essential for an effective viral clearance. The T cells exert diverse functions in defense and antibody response against intracellular and extracellular pathogens (32). The CD4^+^ T cells play the role in secreting cytokines and helping B cells for producing specific neutralizing antibodies, such as CD8^+^ T cells, which are capable of eliminating infected cells (33). Our group previously (14) reported that the CD3^+^T cells were the major cell types that were suppressed in patients with COVID-19, and the reduced CD4^+^ and CD8^+^ T cell counts were predictive of disease progression. Agerer et al. (34) revealed that SARS-CoV-2 escapes CD8 T-cell surveillance *via* mutations in MHC-I restricted epitopes. In our present study, we observed decreased T-cell counts and increased counts of myeloid cells in the peripheral blood collected from patients with COVID-19. Thus, in the current study, we carried out scATAC-seq and scRNA-seq using PBMCs and emphatically depicted the chromatin landscape and transcriptomic immune profiling of patients with COVID-19 in T cells, CD4^+^T cells, and CD8^+^ T cells.

There have been several reports of single cell transcriptomic sequencing from the previous studies on COVID-19. Liao et al. (19) used the scRNA-seq and scTCR-seq to determine the bronchoalveolar lavage fluid (BALF) cell transcriptional signature. Recently, Wilk et al. (22) used the Seq-Well platform scRNA-seq to analyze the PBMCs from patients with COVID-19 and HCs and found signatures of IFN-I-driven inflammation, HLA class II downregulation, and a developing neutrophil population in patients with COVID-19. Lee et al. (18) detected the upregulation of pro-inflammatory cytokine genes in the PBMCs of patients with COVID-19 using the 10× Genomics scRNA-seq, and in severe COVID-19 cases, a type I IFN response coexisted with the TNF/IL-1β-driven inflammation (18). Zhang et al. (35) reported that most cell types from SARS-COV-2-infected patients showed a strong interferon-α response and an overall acute inflammatory response. Zhu et al. (31) reported that in patients with COVID-19, XAF1-, TNF-, and FAS-induced T-cell apoptosis were observed, and STAT1 and IRF3 signaling pathways were activated. In addition, Lucas et al. (36) analyzed an immune-response profile associated with severe COVID-19 outcome and early immune signatures that correlated with divergent disease trajectories). Ni et al. (37) reported that patients with COVID-19 who experienced severe symptoms had associated defective cellular immunity. Xiong et al. (23) characterized the SARS-COV-2-specific cytotoxic T cells by single-cell immune profiling and indicated that SARS-COV-2 infection can induce virus-reactive cytotoxic T cells. Li et al. (20) reported a single cell RNA and an immune repertoire profiling in patients with COVID-19 and revealed a novel neutralizing antibody. Zheng et al. (24) compared immune cell types in peripheral blood collected from young and old subjects and patients with COVID-19 and provided a comprehensive atlas of human circulating immune cell aging.

In this study, we presumed that the epigenetic status of T cells is functionally relevant to the pathogenesis stage of COVID-19, which is supported by our data obtained from the scATAC-seq and scRNA-seq. To our knowledge, this is the first epigenetic landscape analysis for T cells of COVID-19 cases in single-cell resolution. Besides the discoveries of reported transcriptomic immune profiles of T cells in patients with COVID-19, our scRNA-seq data showed that histone-related genes were highly expressed in the total T cells, CD4^+^ T, and CD8^+^ T cells, both in SCPs cases. In addition, decreased Th1 cells were observed in SCPs and MPs in the CD4^+^ T cells. Activation marker (CD69) and HLA class II genes (HLA-DRA, HLA-DRB1, and HLA-DRB5) were upregulated in SCPs of the CD8^+^T cells. The scATAC-seq data of the peak-enriched genes indicated that an inflammatory state of T cells combined with a possible deficiency in quantity and the function of CD4^+^ T cells may play a key role to orchestrate the CD8^+^ T-cell-mediated anti-viral effects. The scRNA-seq data corroborated the results of scATAC-seq. Our results together present a landscape of chromatin epigenetic status and transcriptomic immune profiles of T cells in patients with COVID-19. The landscape indicates that a T-cell inflammatory state and a deficiency of CD4^+^ T cells in SCPs may contribute to the mechanisms underlying the pathogenesis of and recovery from COVID-19. This, in turn, sheds light on the possibility of T-cell immunotherapy for COVID-19.

## Data Availability Statement

The original contributions presented in the study are publicly available. The raw data have been deposited in the China National GeneBank DataBase and can be found here: https://db.cngb.org/, CNP0001507.

## Ethics Statement

The studies involving human participants were reviewed and approved by the Research Ethics Committee of Shanghai Public Health Clinical Center. The patients/participants provided their written informed consent to participate in this study.

## Author Contributions

XZ, HL, TZ, and SL conceived and designed the study. BW, ZZ, MG, and BQ isolated the PBMCs. YLiu, YLia, ZW, YS, LiL, and JC collected and analyzed the clinical data. BW, XR, CW, HY, LC, YLiu, XP, CX, BN, MZ, LinL, and FL helped in RNA isolation and cDNA library construction. SL, XZ, and BW performed the data collection. SL, XZ, and YLiu analyzed the data. SL and XZ plotted the figures and drafted the manuscript. SL, XZ, HL, and TZ revised the paper and all authors reviewed and/or edited the paper.

## Conflict of Interest

The authors declare that the research was conducted in the absence of any commercial or financial relationships that could be construed as a potential conflict of interest.
