# Supplementary material for: Epigenetic Landscapes of Single-Cell Chromatin Accessibility and Transcriptomic Immune Profiles of T Cells in COVID-19 Patients
Source: Front Immunol. 2021 Feb 24;12:625881. doi: 10.3389/fimmu.2021.625881 (PMC7943924; doi:10.3389/fimmu.2021.625881)
Supplement: Supplementary file 1 [file Table_1.DOCX]

| Donor | Age （Years） | Sex | scRNA-seq | scATAC-seq |
| --- | --- | --- | --- | --- |
| Health volunteer #1 | 32 | M | Yes | Yes |
| Health volunteer #2 | 62 | M | Yes | Yes |
| Health volunteer #3 | 48 | F | Yes | Yes |
| Health volunteer #4 | 26 | M | Yes |  |
| Health volunteer #5 | 30 | M | Yes |  |
| Moderate case #1 | 29 | M | Yes | Yes |
| Moderate case #2 | 54 | F | Yes | Yes |
| Moderate case #3 | 55 | M | Yes | Yes |
| Moderate case #4 | 52 | M | Yes |  |
| Moderate case #5 | 49 | F | Yes |  |
| Crtical case #1 | 76 | M | Yes | Yes |
| Crtical case #2 | 76 | M | Yes | Yes |
| Crtical case #3 | 63 | M | Yes |  |
| Severe case #1 | 55 | M | Yes |  |
| Severe case #2 | 65 | M | Yes | Yes |

Supplementary table 1 Informations of patients with COVID-19
