# Supplementary material for: Epigenetic Landscapes of Single-Cell Chromatin Accessibility and Transcriptomic Immune Profiles of T Cells in COVID-19 Patients
Source: Front Immunol. 2021 Feb 24;12:625881. doi: 10.3389/fimmu.2021.625881 (PMC7943924; doi:10.3389/fimmu.2021.625881)
Supplement: Supplementary file 15 [file Data_Sheet_1.PDF]

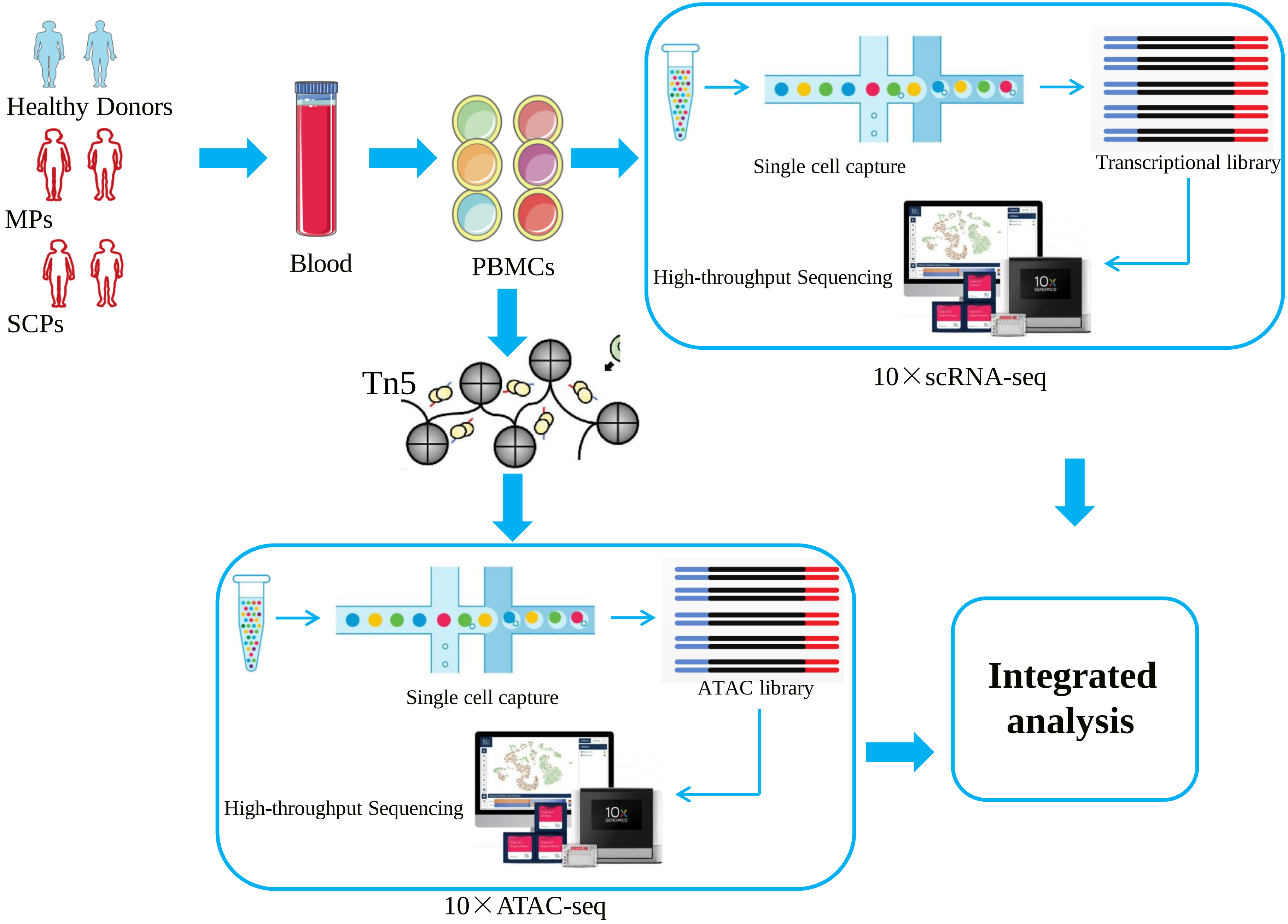

FIGURE S1. The flow diagram of the project.

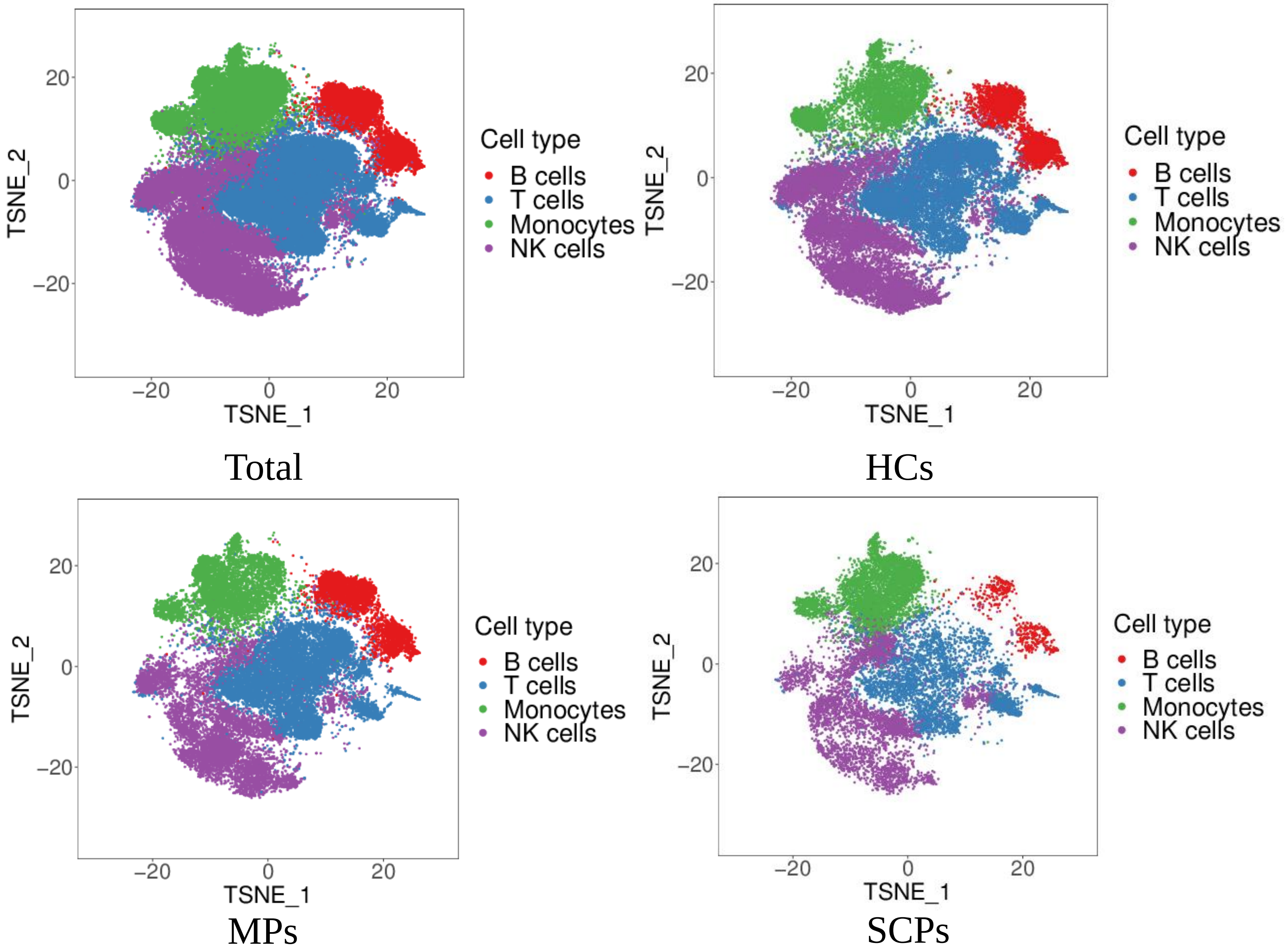

FIGURE S2. (A) An overview of T, B, NK and monocyte cells in the PBMC of COVID-19 patients by single-cell ATAC. The t-SNE plot shows a comparison of the clustering distribution of each groups.

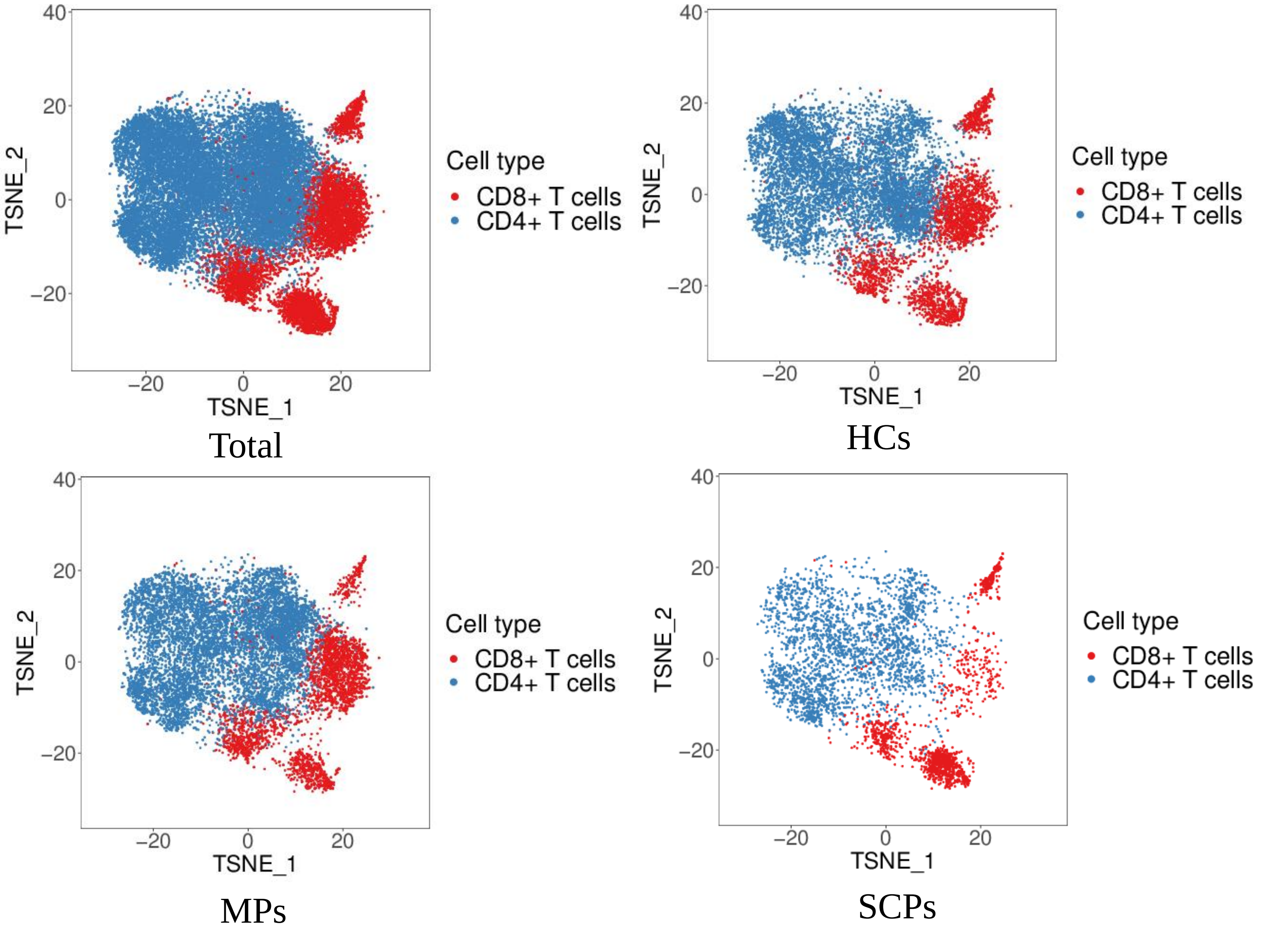

FIGURE S2. (B) Characterization of T cells in the PBMC of COVID-19 patients by single-cell ATAC. The t-SNE plot shows that the T cells re-clustered into $CD4^{+}$ T cells and $CD8^{+}$ T cells.

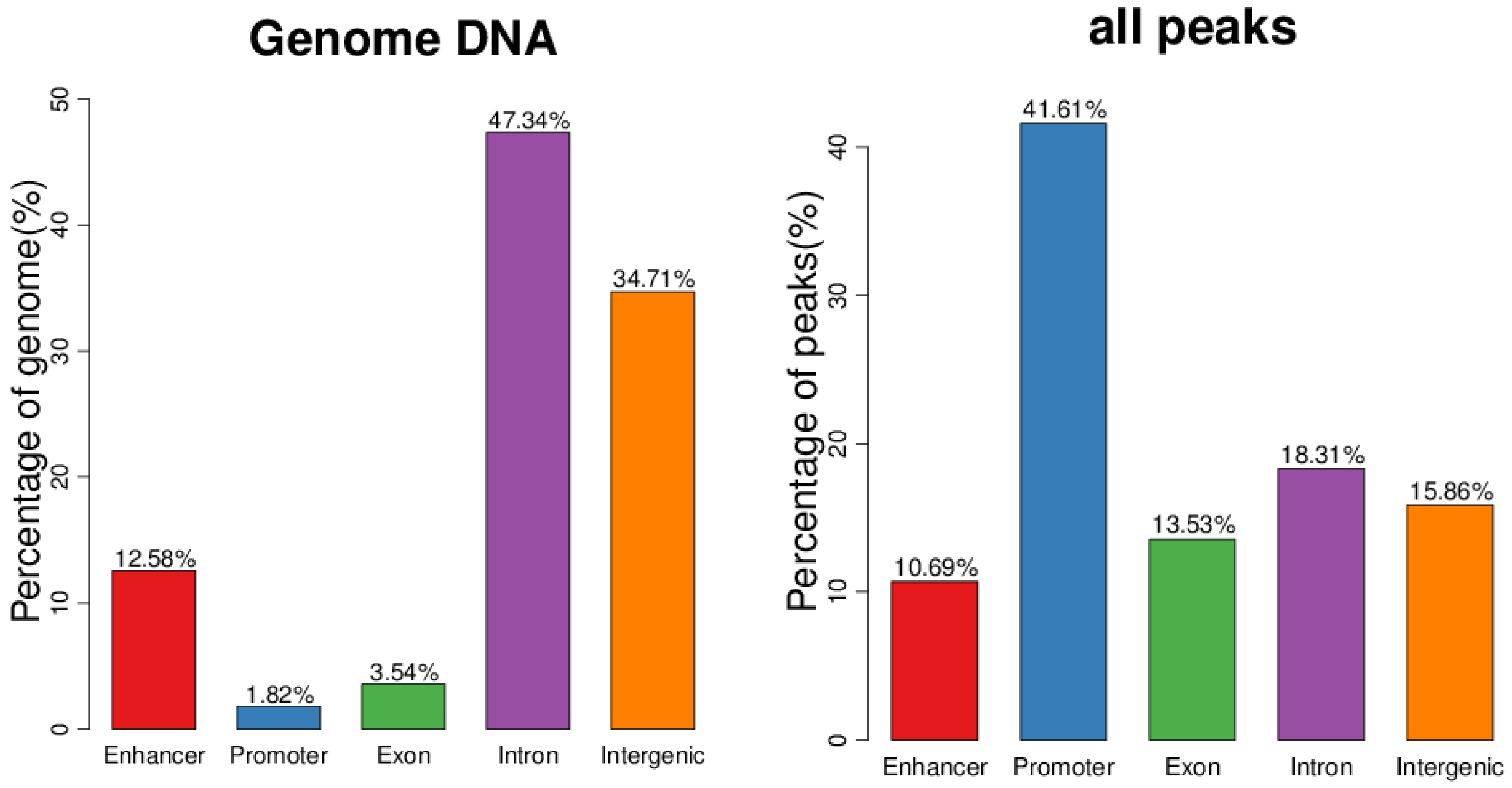

FIGURE S3. (A) Distribution of genomic features and percentage of all different peaks located in the region of enhancer, promoter, exon element, intron and intergenic.

T cells

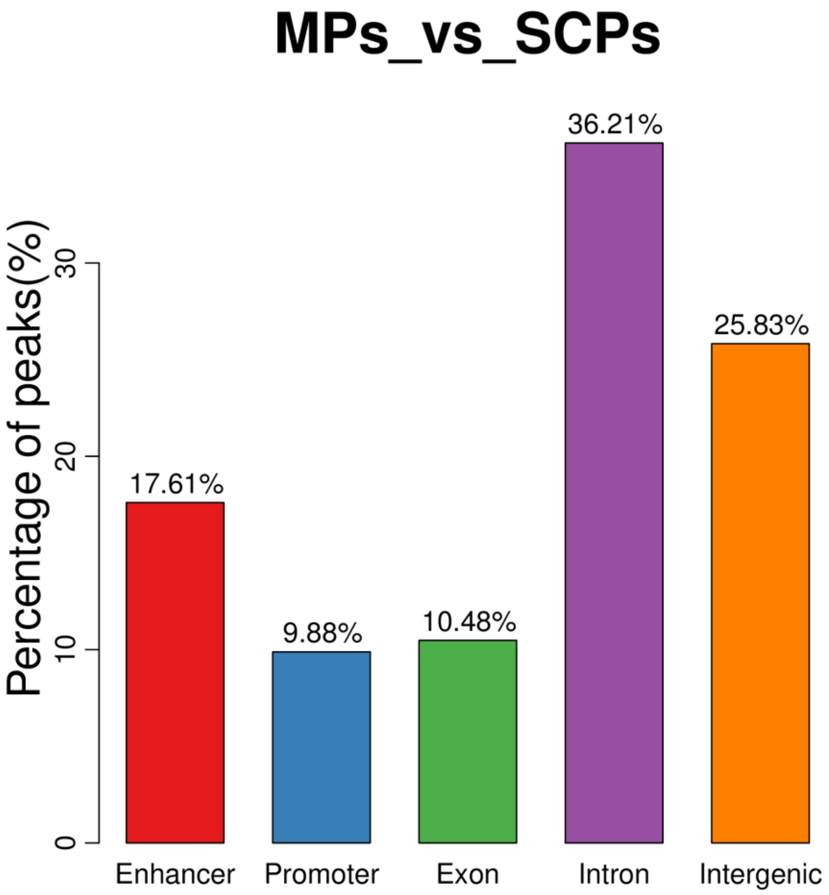

FIGURE S3. (B) Distribution of genomic features of different peaks from T cells in the group of MPs vs. SCPs. 5 genomic features were studied, enhancer, promoter, exon element, intron and intergenic.

CD4^{+} T cells

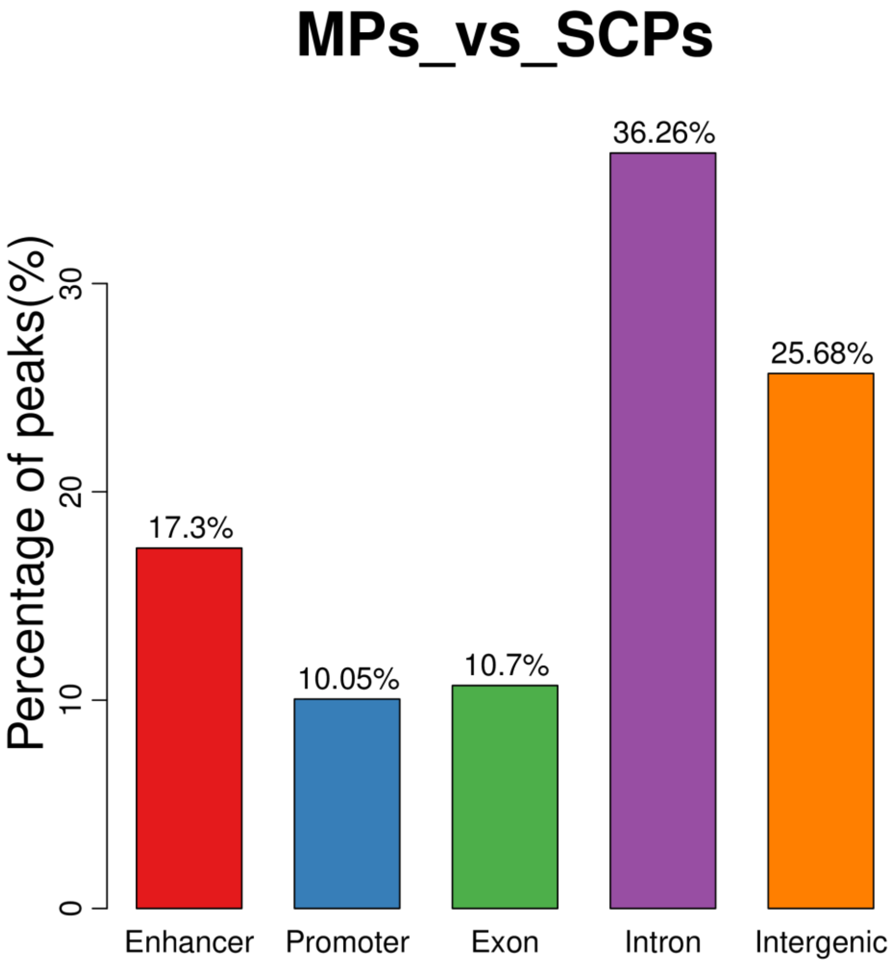

FIGURE S3. (C) Distribution of genomic features of different peaks from CD4^{+} T cells in the group of MPs vs. SCPs. 5 genomic features were studied, enhancer, promoter, exon element, intron and intergenic.

CD8$^+$ T cells

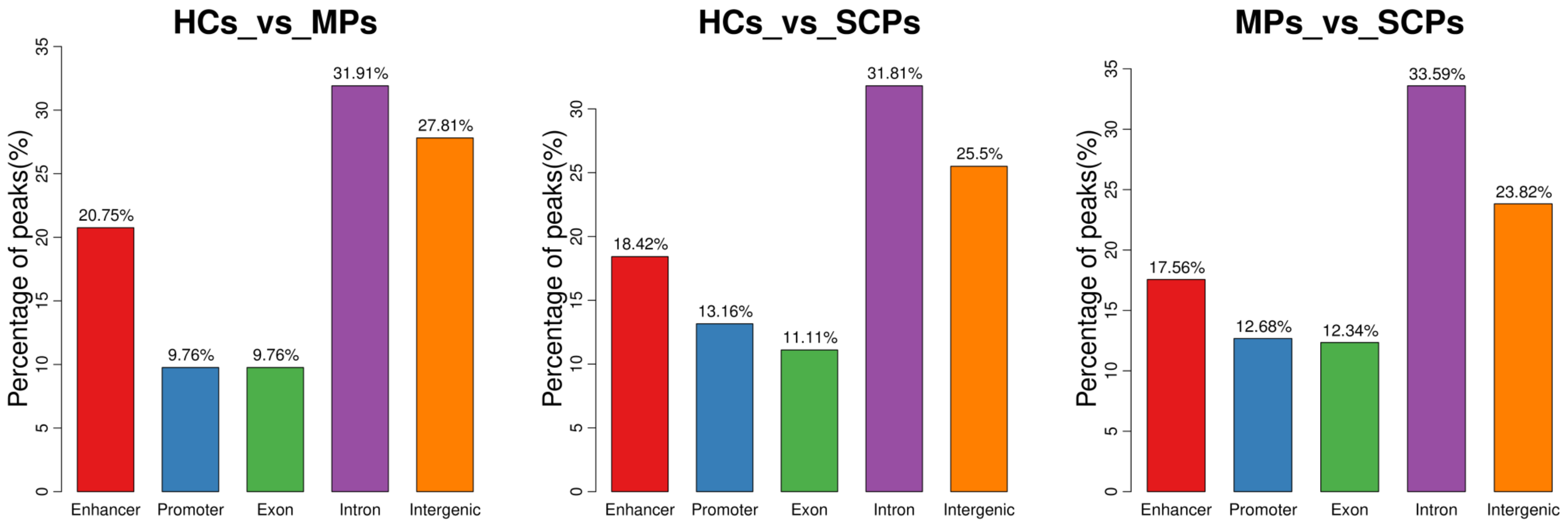

FIGURE S3. (D) Distribution of genomic features of different peaks from CD8$^+$ T cells among different groups. 5 genomic features were studied, enhancer, promoter, exon element, intron and intergenic.

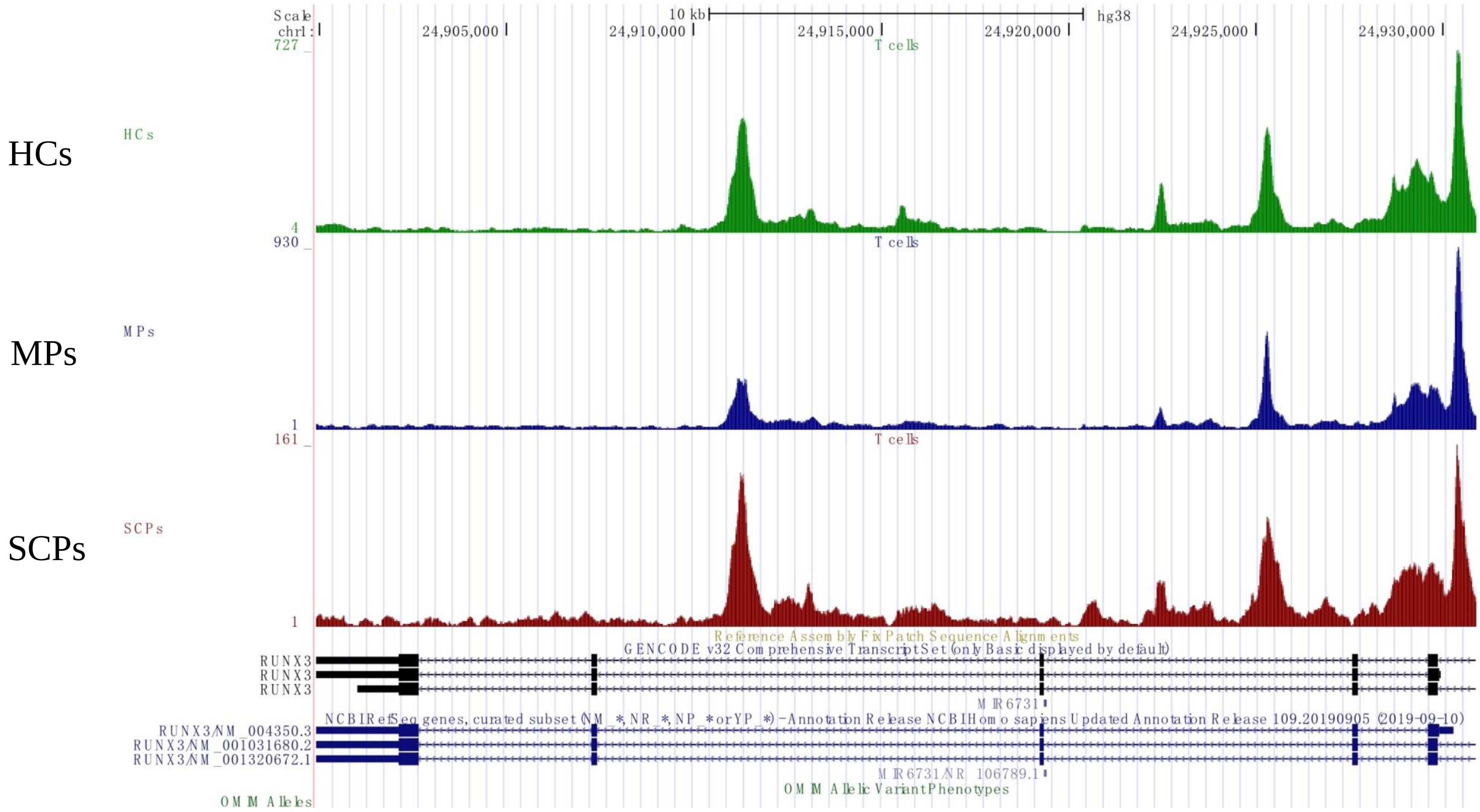

FIGURE S4. (A) Single-cell ATAC-seq genome tracks for the RUNX3 locus in T cells among HCs and patients with server/critical and moderate COVID-19 infection.

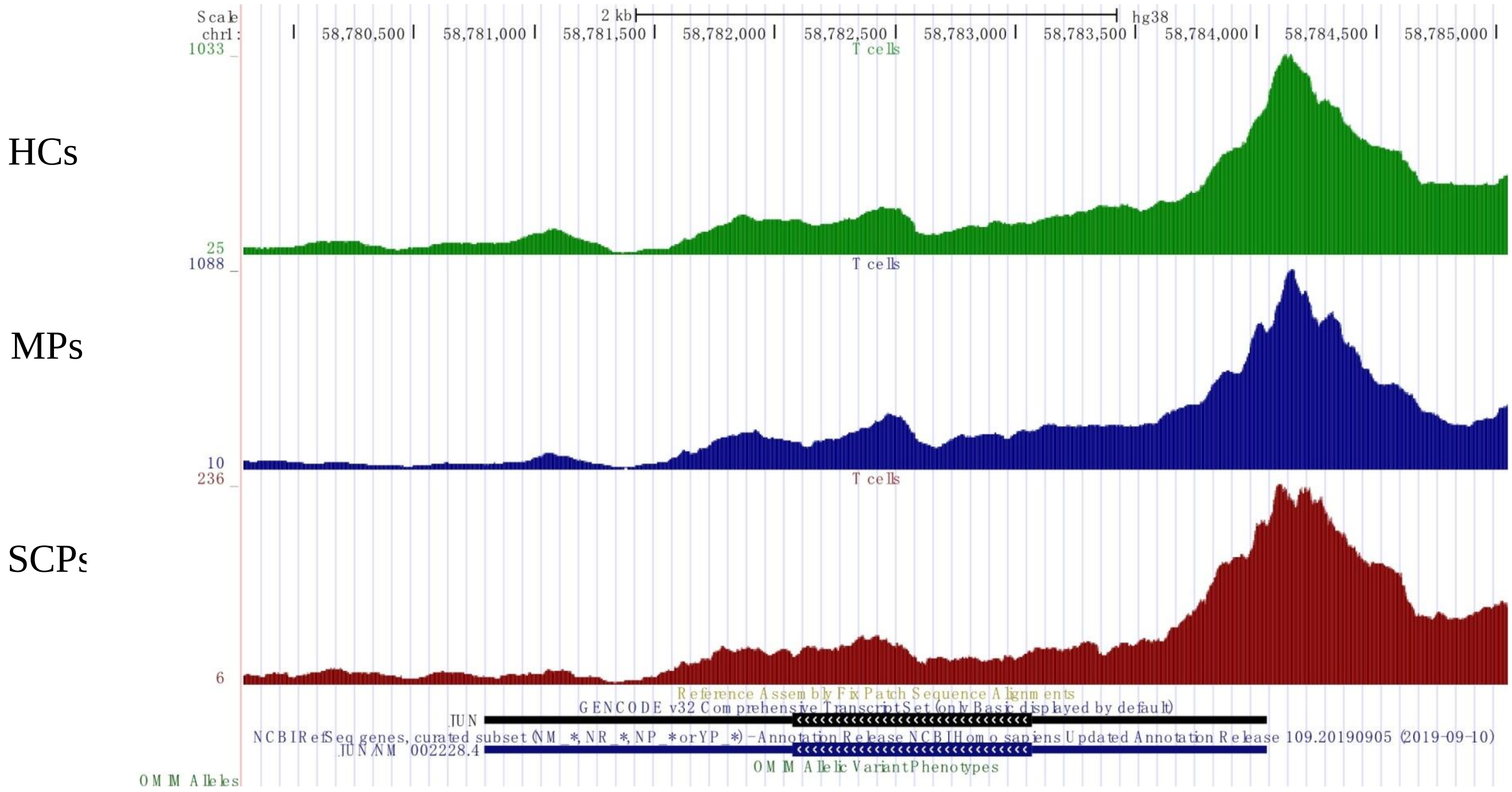

FIGURE S4. (B) Single-cell ATAC-seq genome tracks for the JUN locus in T cells among HCs and patients with server/critical and moderate COVID-19 infection.

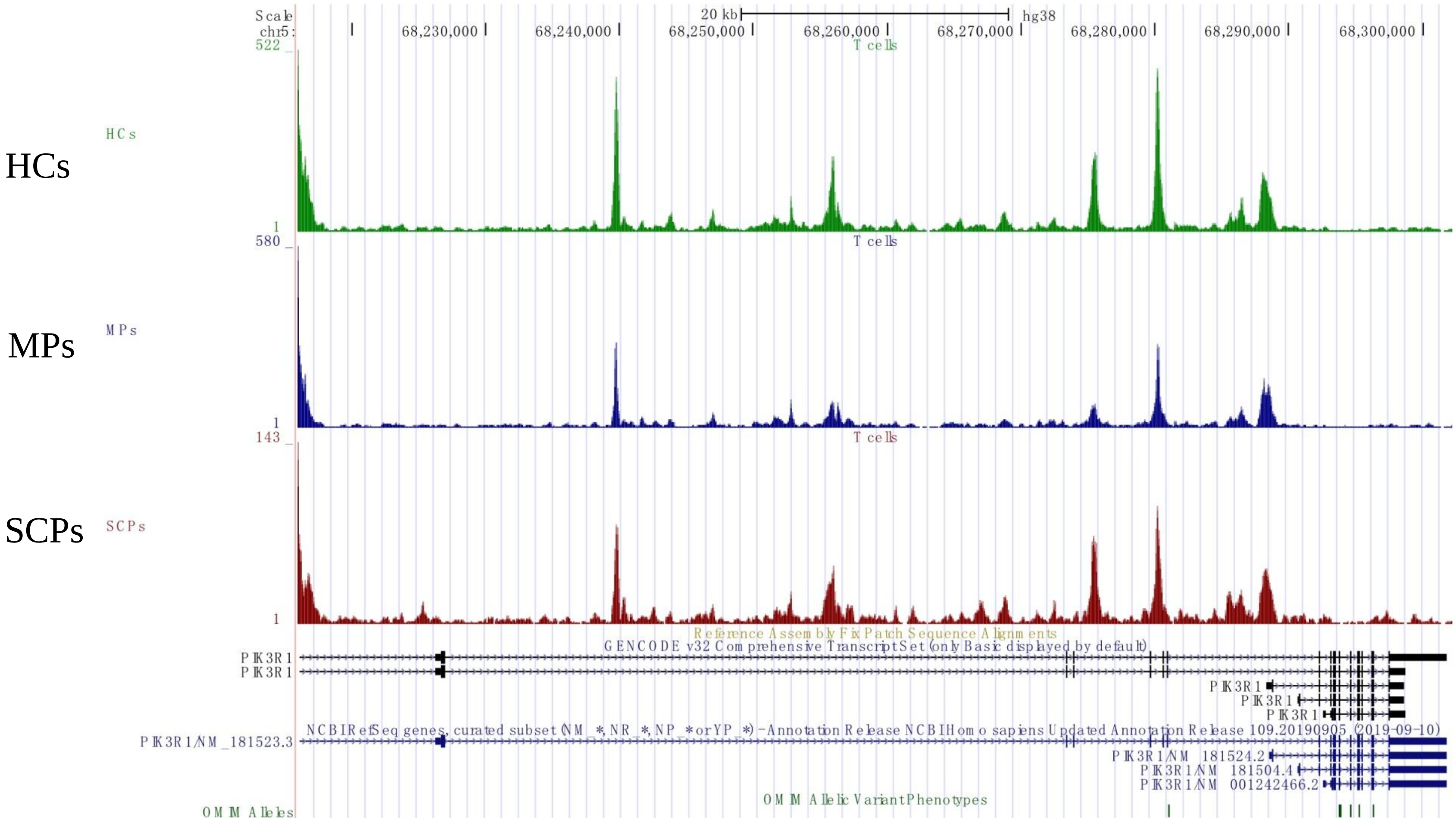

FIGURE S4. (C) Single-cell ATAC-seq genome tracks for the PIK3R1 locus in T cells among HCs and patients with server/critical and moderate COVID-19 infection.

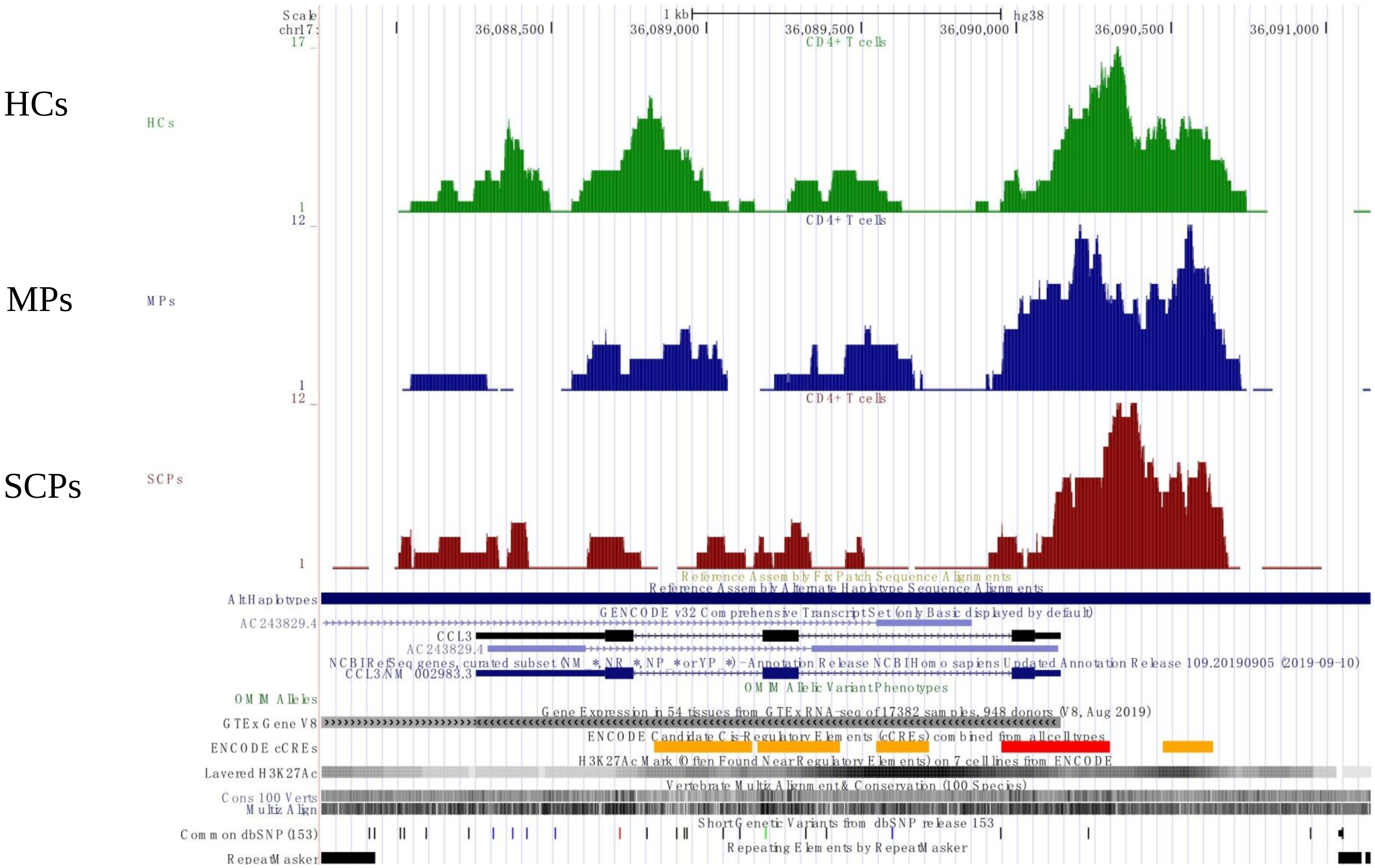

FIGURE S4. (D) Single-cell ATAC-seq genome tracks for the CCL3 locus in $CD4^+$ T cells among HCs and patients with server/critical and moderate COVID-19 infection.

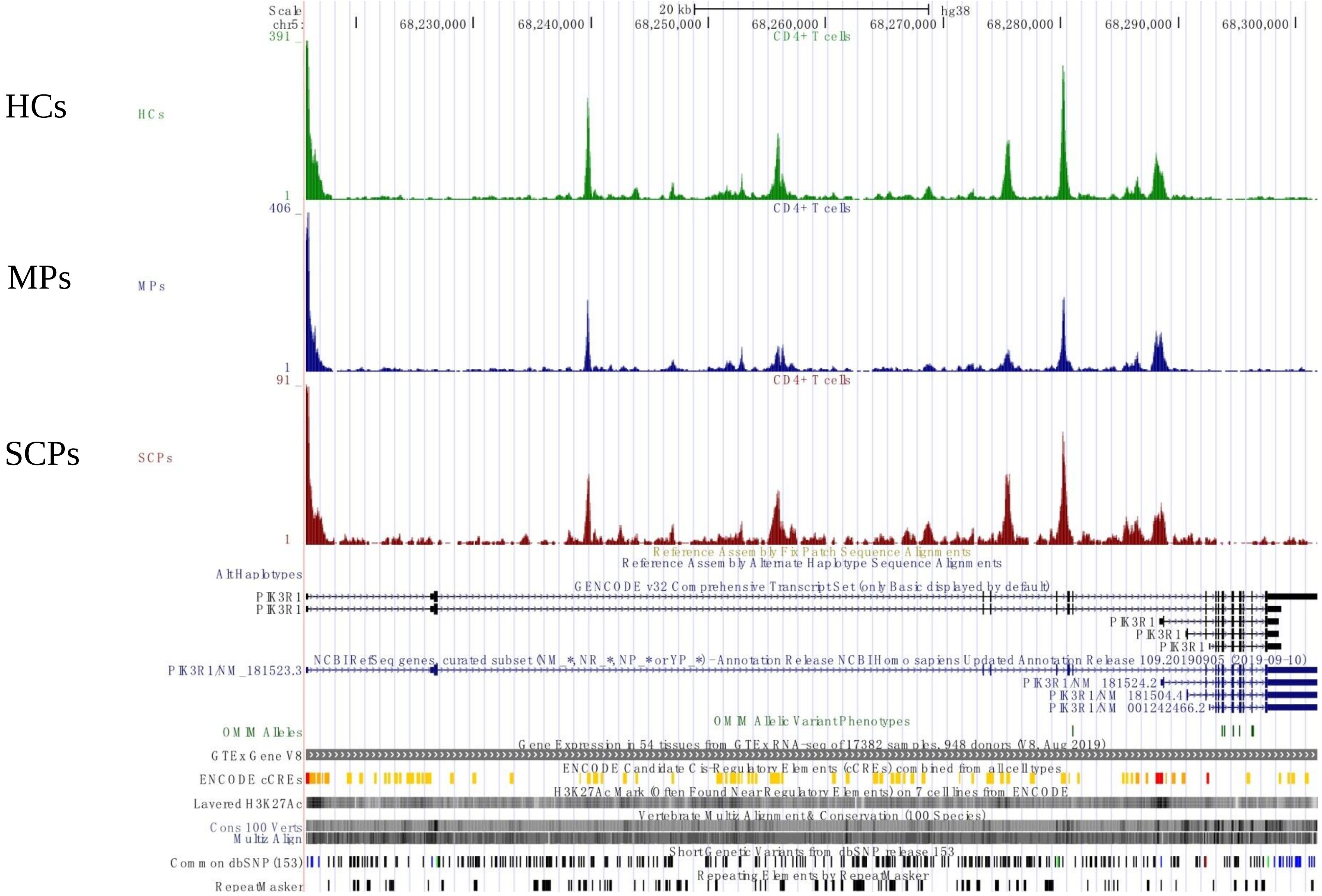

FIGURE S4. (E) Single-cell ATAC-seq genome tracks for the PIK3R1 locus in CD4+ T cells among HCs and patients with server/critical and moderate COVID-19 infection.

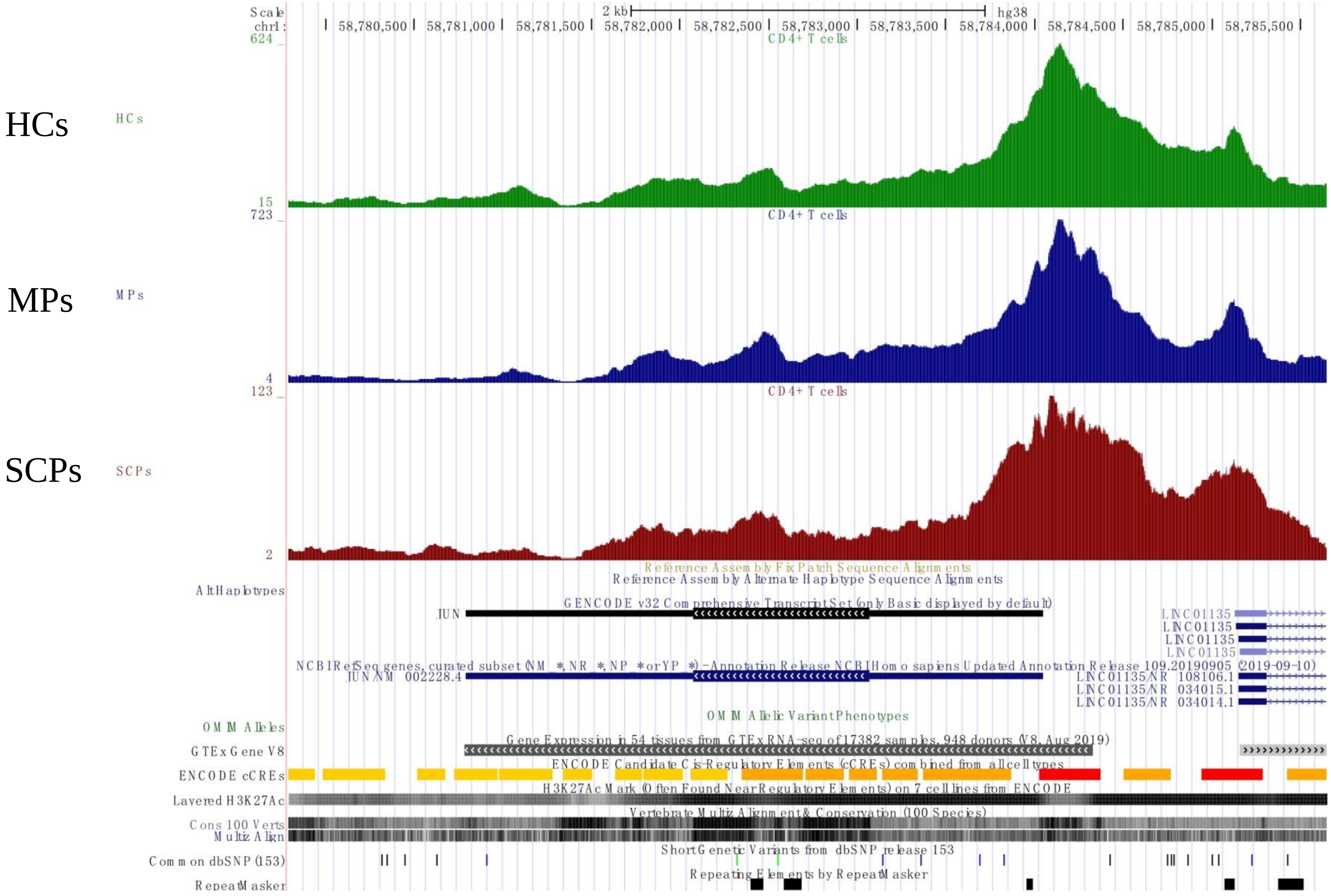

FIGURE S4. (F) Single-cell ATAC-seq genome tracks for the JUN locus in $CD4^+$ T cells among HCs and patients with server/critical and moderate COVID-19 infection.

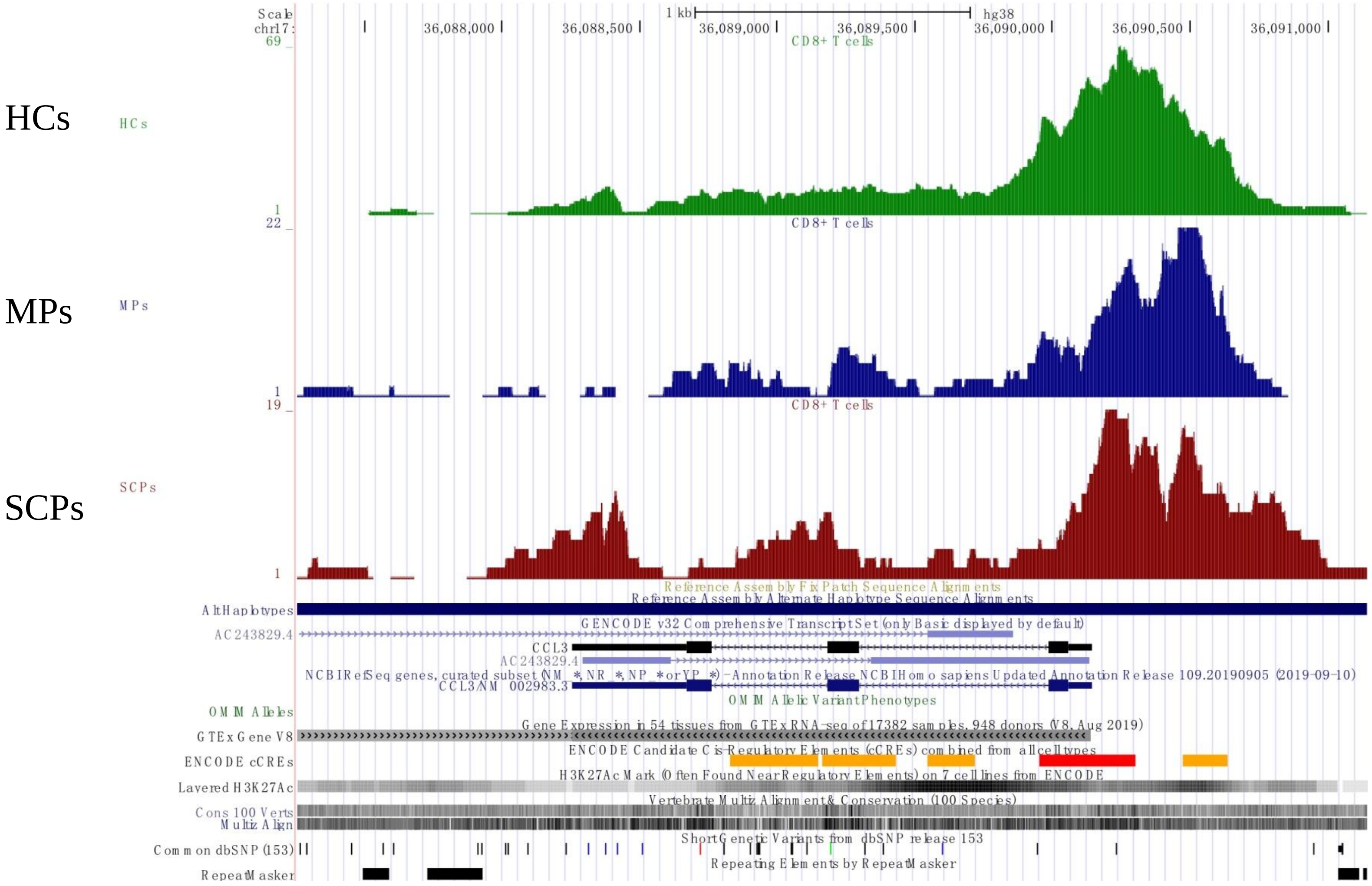

FIGURE S4. (G) Single-cell ATAC-seq genome tracks for the CCL3 locus in CD8^{+} T cells among HCs and patients with server/critical and moderate COVID-19 infection.

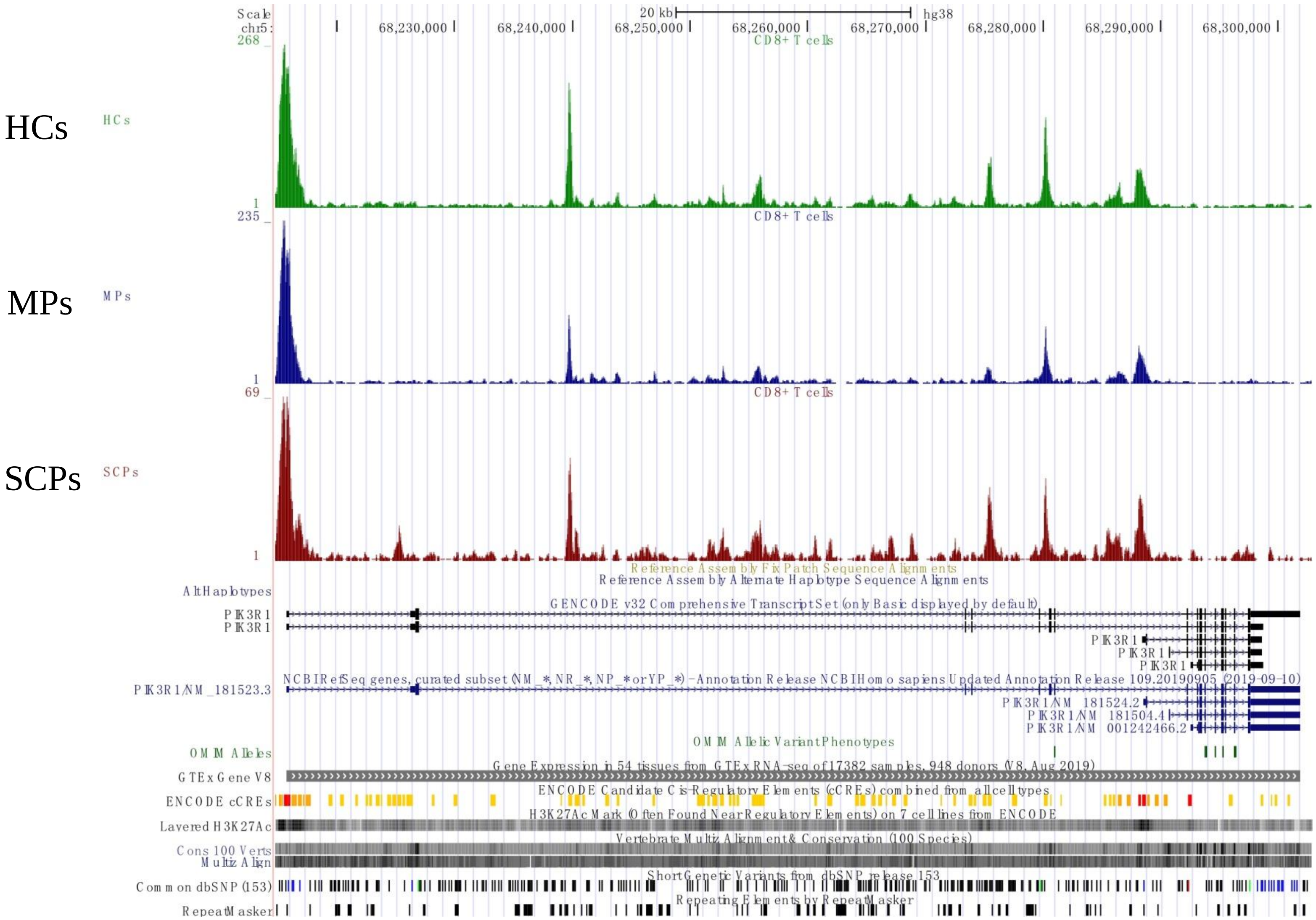

FIGURE S4. (H) Single-cell ATAC-seq genome tracks for the PIK3R1 locus in CD8+ T cells among HCs and patients with server/critical and moderate COVID-19 infection.

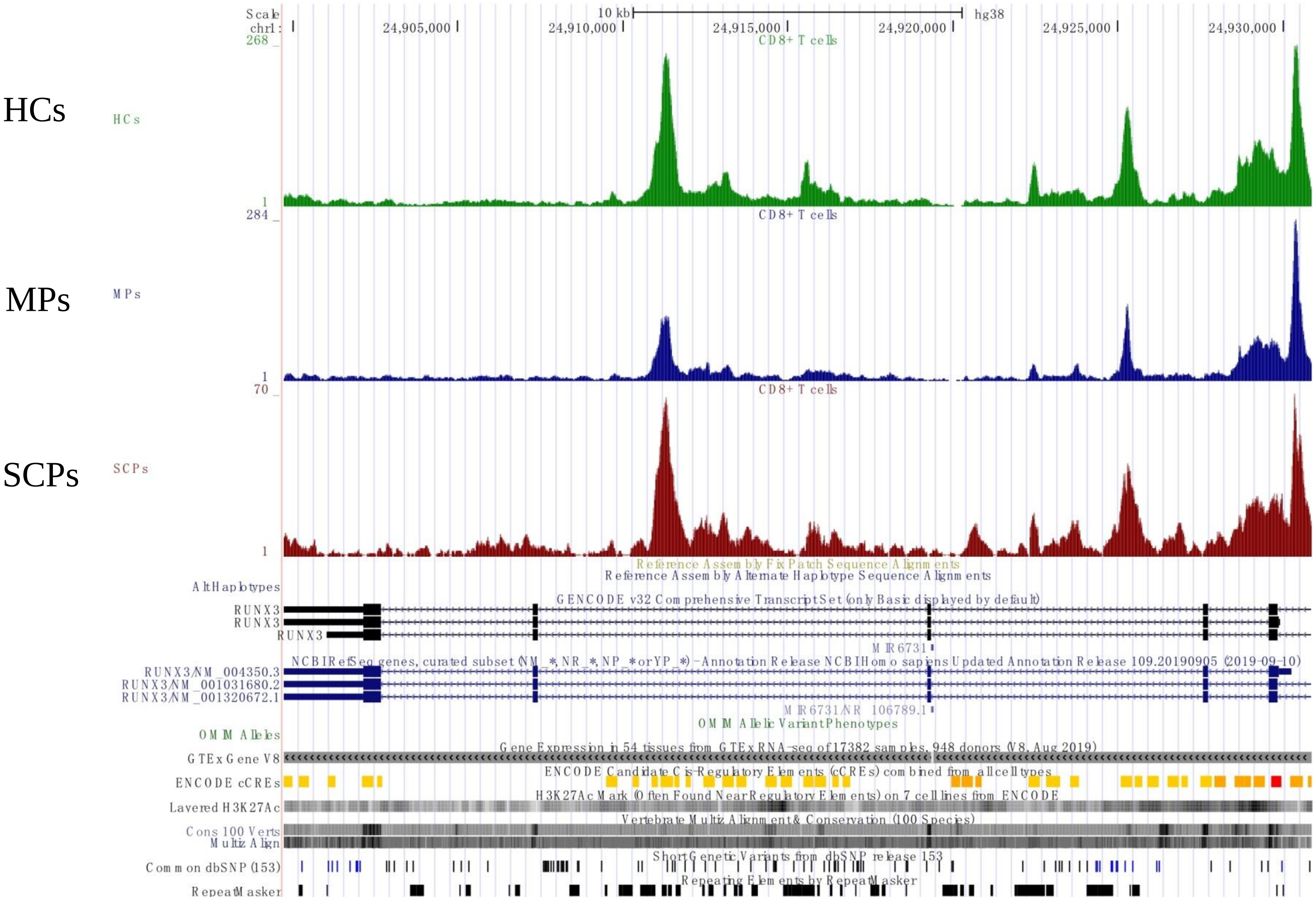

FIGURE S4. (I) Single-cell ATAC-seq genome tracks for the RUNX3 locus in CD8^{+} T cells among HCs and patients with server/critical and moderate COVID-19 infection.

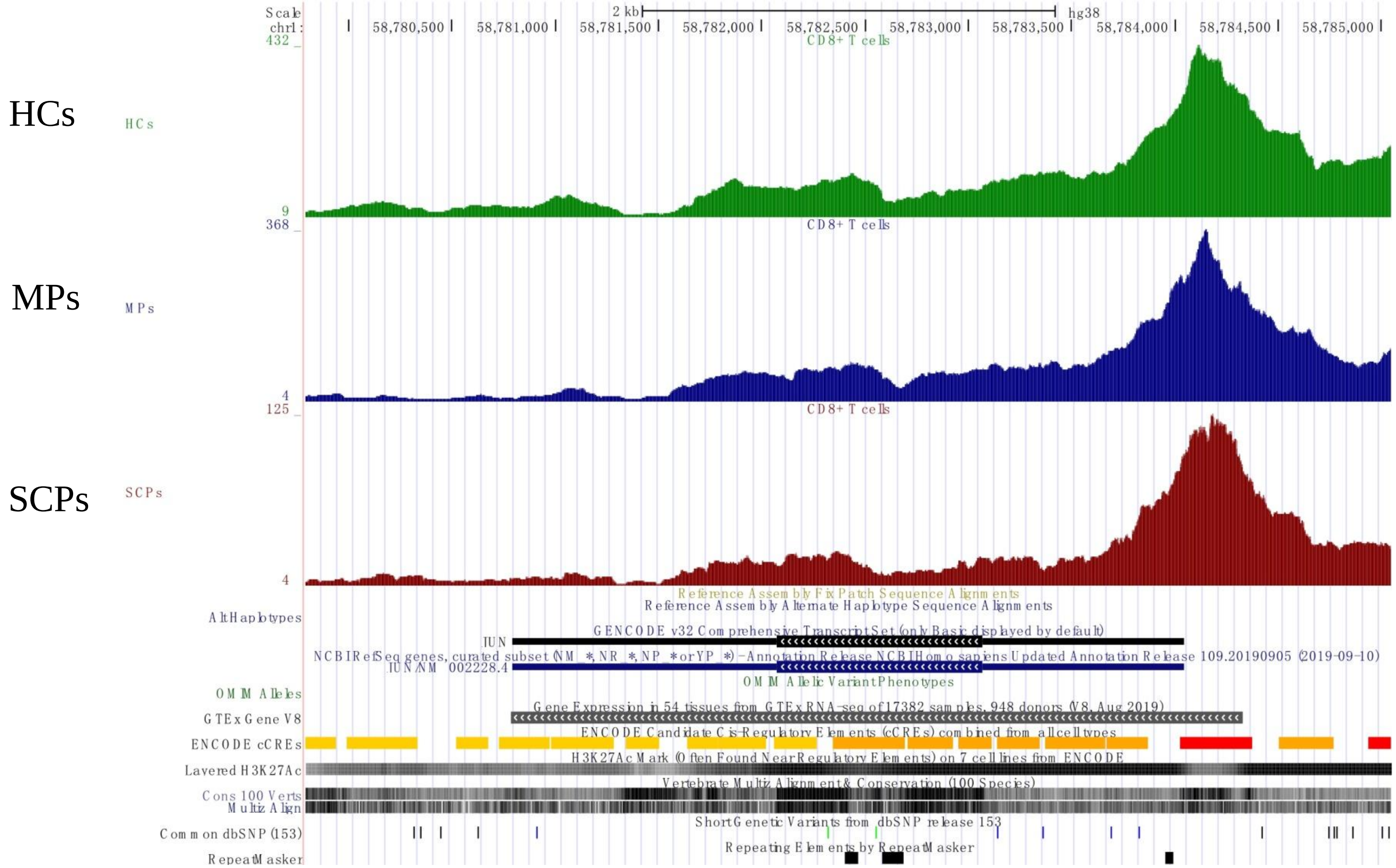

FIGURE S4. (J) Single-cell ATAC-seq genome tracks for the JUN locus in $CD8^+$ T cells among HCs and patients with server/critical and moderate COVID-19 infection.

PIK3R1 T cells

HCs MPs SCPs

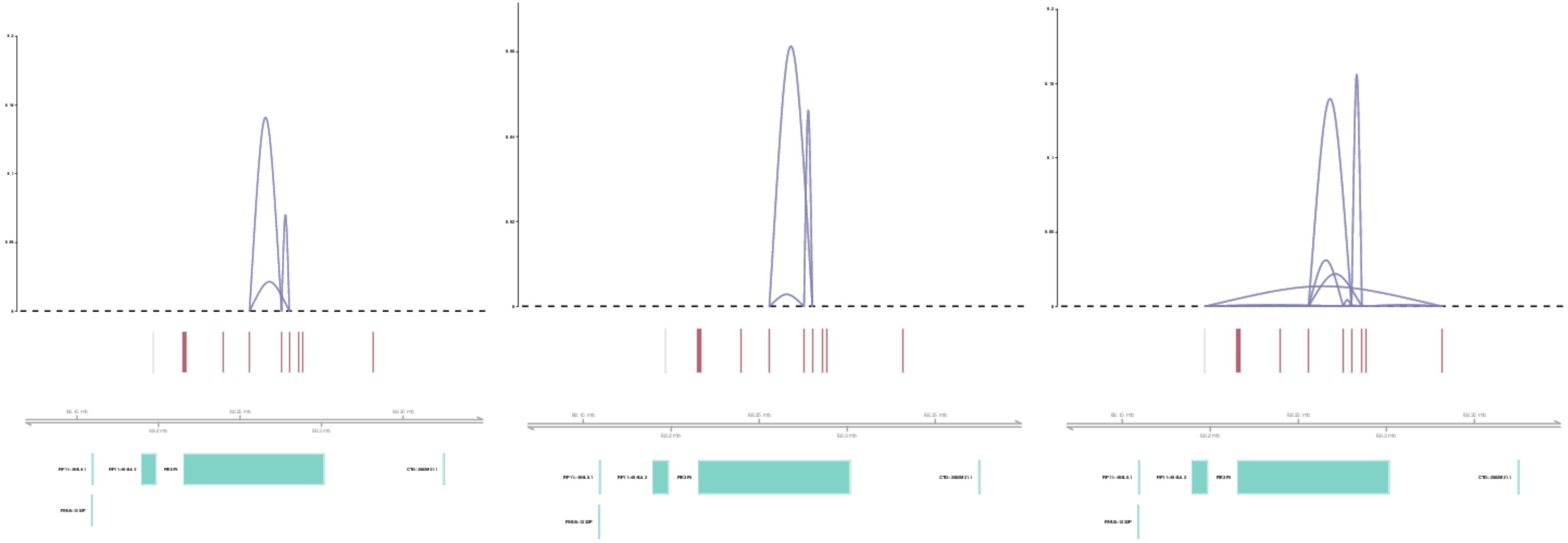

FIGURE S5 (A). The reconstruction of genome-wide cis-regulatory interaction networks for certain regions across specific genes for all cell types among three groups in T cells.

RUNX3 T cells

HCs MPs SCPs

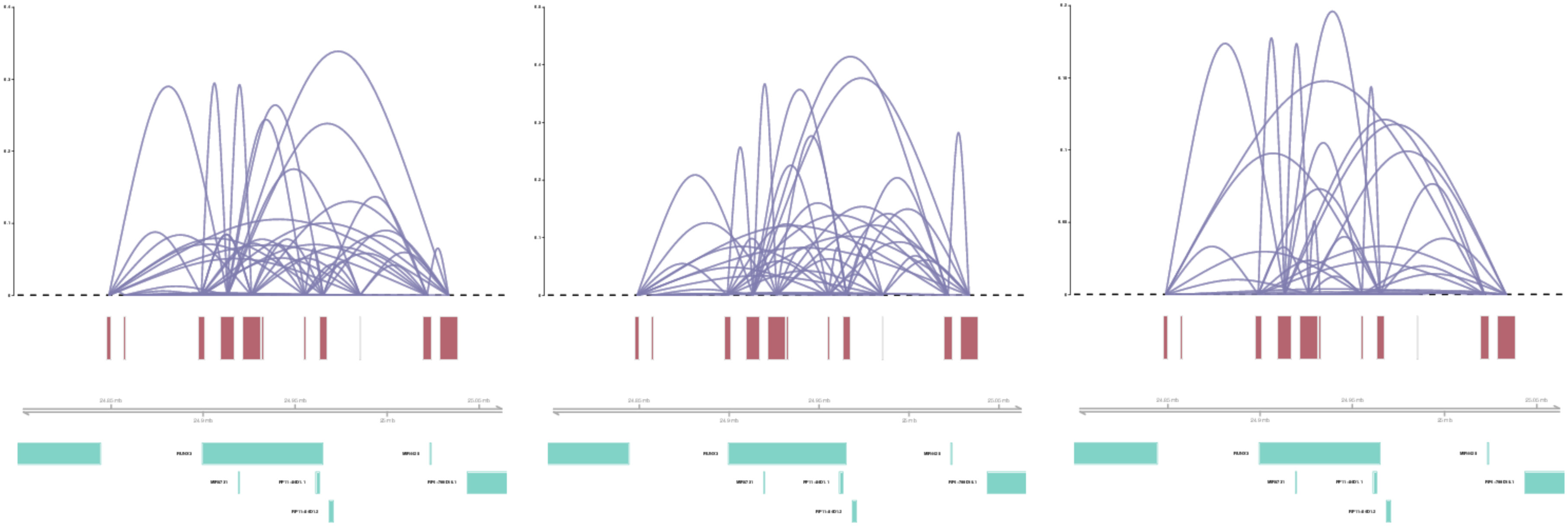

FIGURE S5 (B). The reconstruction of genome-wide cis-regulatory interaction networks for certain regions across specific genes for all cell types among three groups in T cells.

JUN T cells

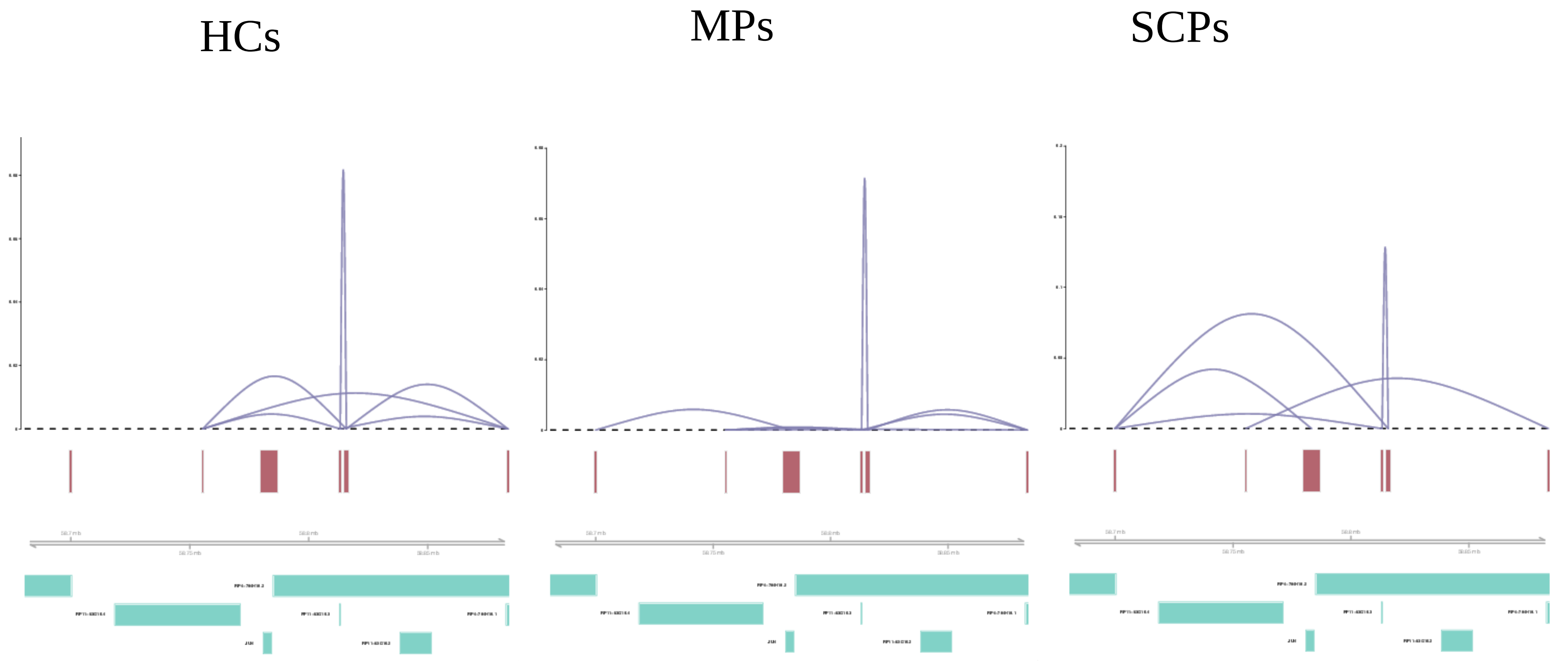

FIGURE S5 (C). The reconstruction of genome-wide cis-regulatory interaction networks for certain regions across specific genes for all cell types among three groups in T cells.

CCL3 CD4$^+$ T cells

HCs MPs SCPs

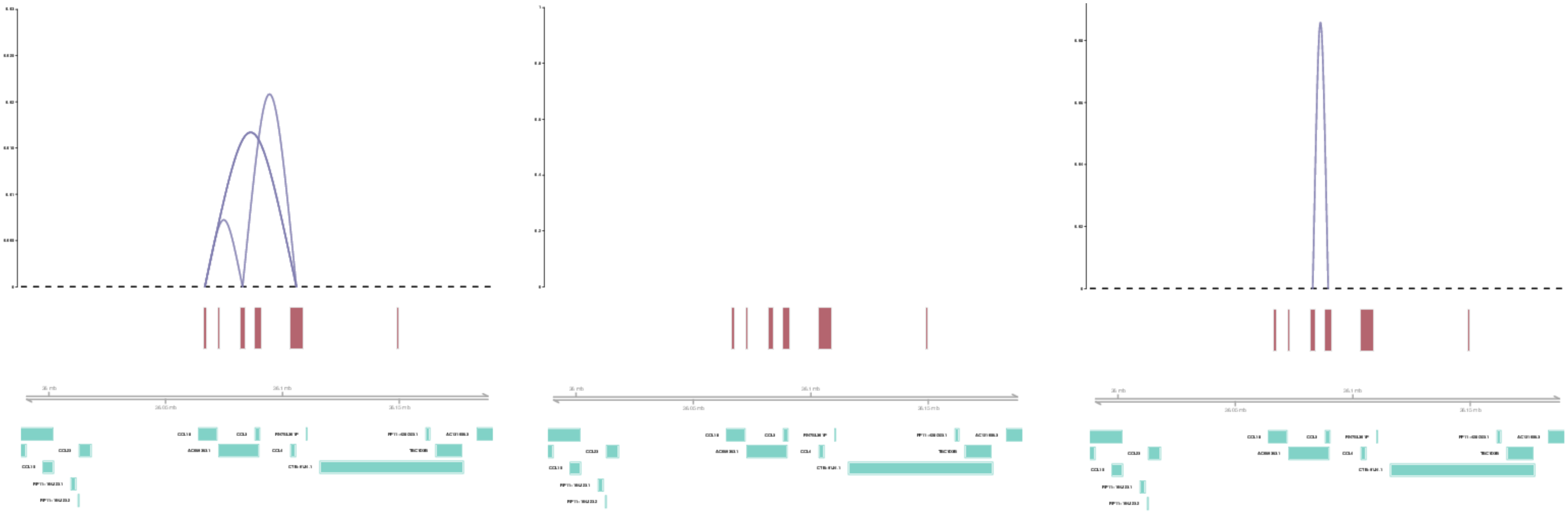

FIGURE S5 (D). The reconstruction of genome-wide cis-regulatory interaction networks for certain regions across specific genes for all cell types among three groups in CD4$^+$ T cells.

PIK3R1 CD4$^+$ T cells

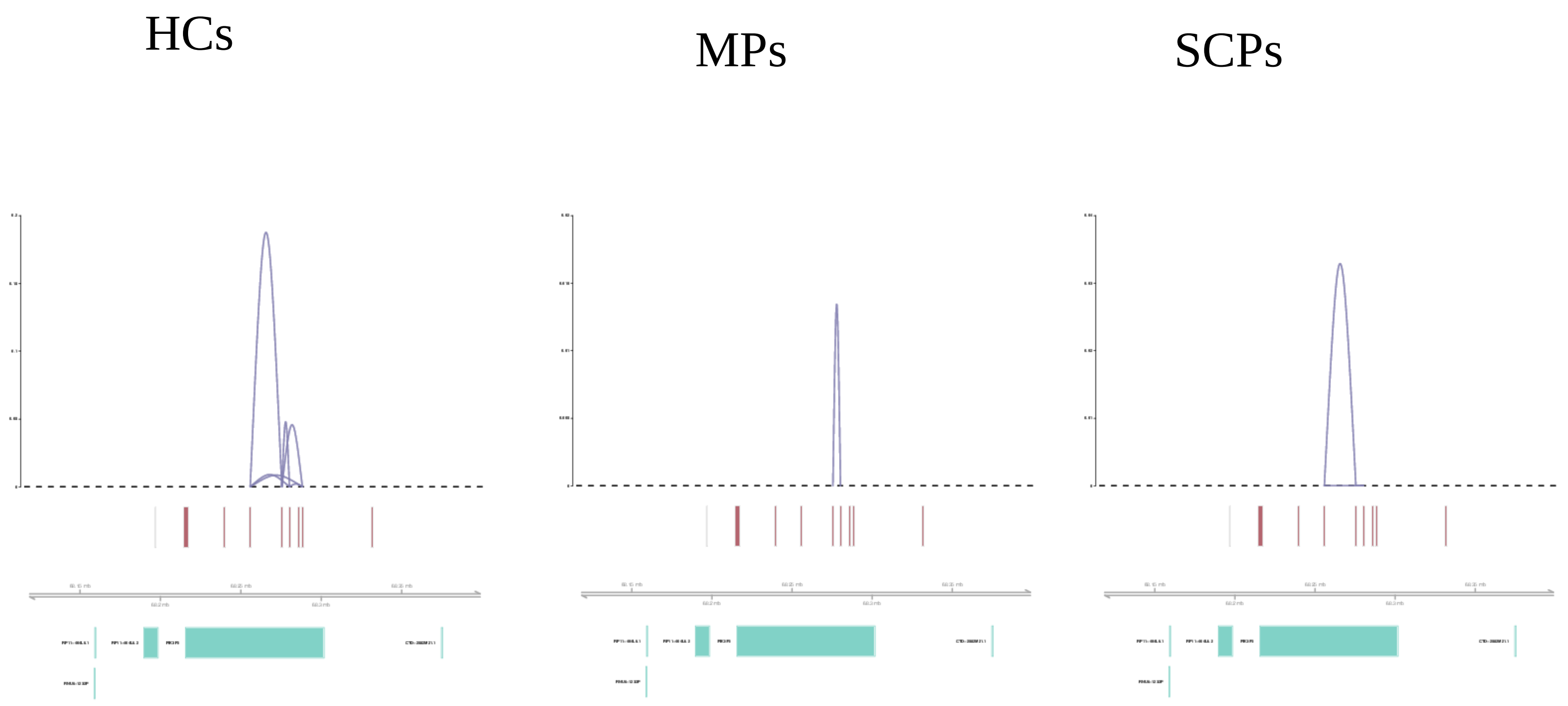

FIGURE S5 (E). The reconstruction of genome-wide cis-regulatory interaction networks for certain regions across specific genes for all cell types among three groups in CD4$^+$ T cells.

JUN CD4$^+$ T cells

HCs MPs SCPs

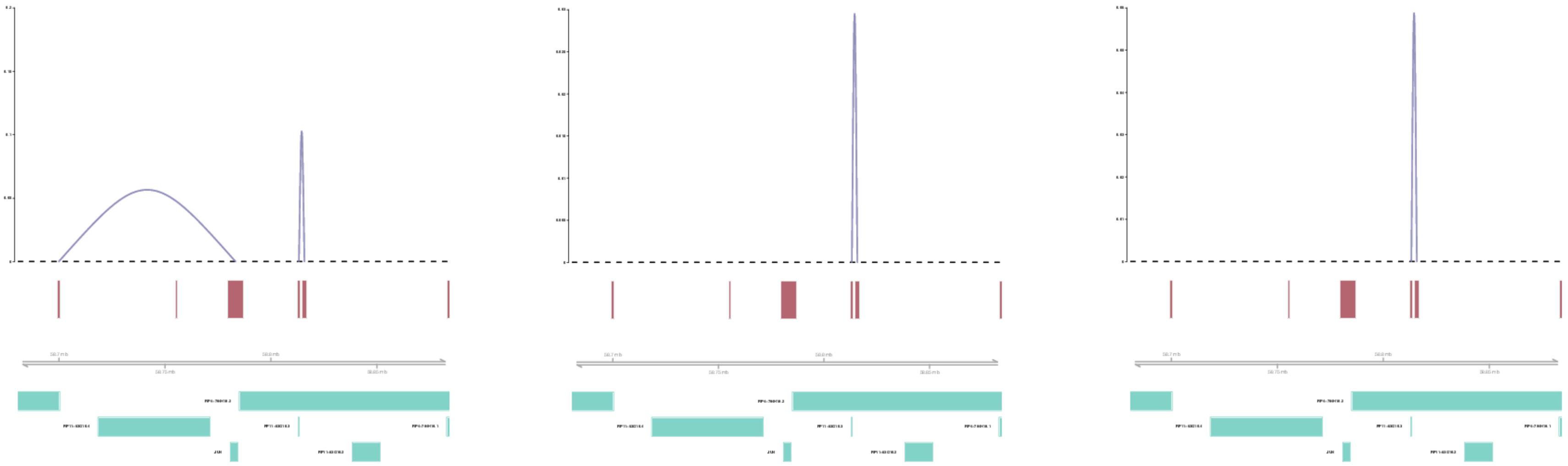

FIGURE S5 (F). The reconstruction of genome-wide cis-regulatory interaction networks for certain regions across specific genes for all cell types among three groups in CD4$^+$ T cells.

CCL3 $CD8^+$ T cells

HCs MPs SCPs

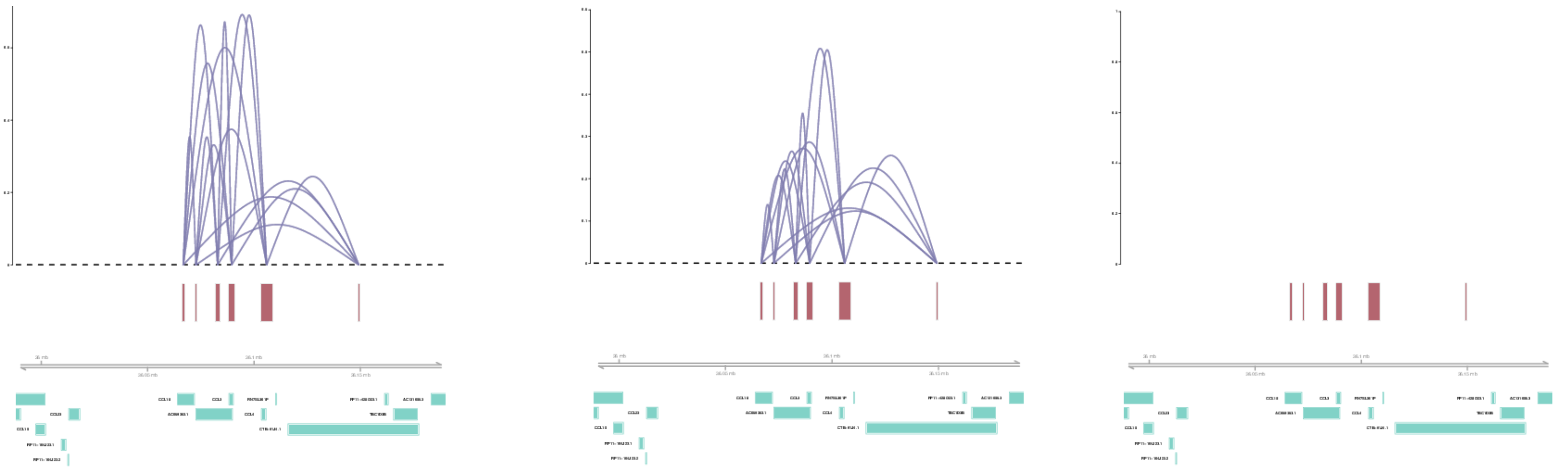

FIGURE S5 (G). The reconstruction of genome-wide cis-regulatory interaction networks for certain regions across specific genes for all cell types among three groups in $CD8^+$ T cells.

PIK3R1 CD8$^+$ T cells

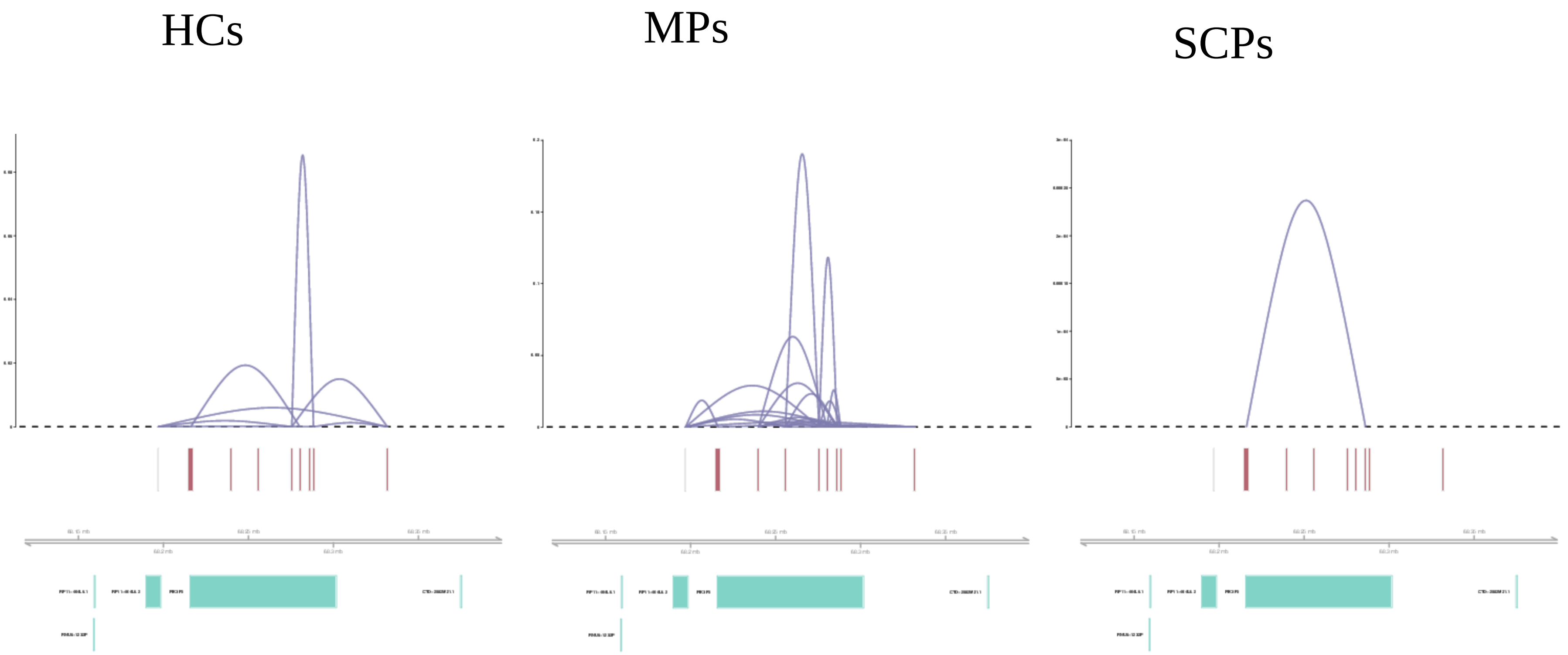

FIGURE S5 (H). The reconstruction of genome-wide cis-regulatory interaction networks for certain regions across specific genes for all cell types among three groups in CD8$^+$ T cells.

RUNX3 CD8$^+$ T cells

HCs MPs SCPs

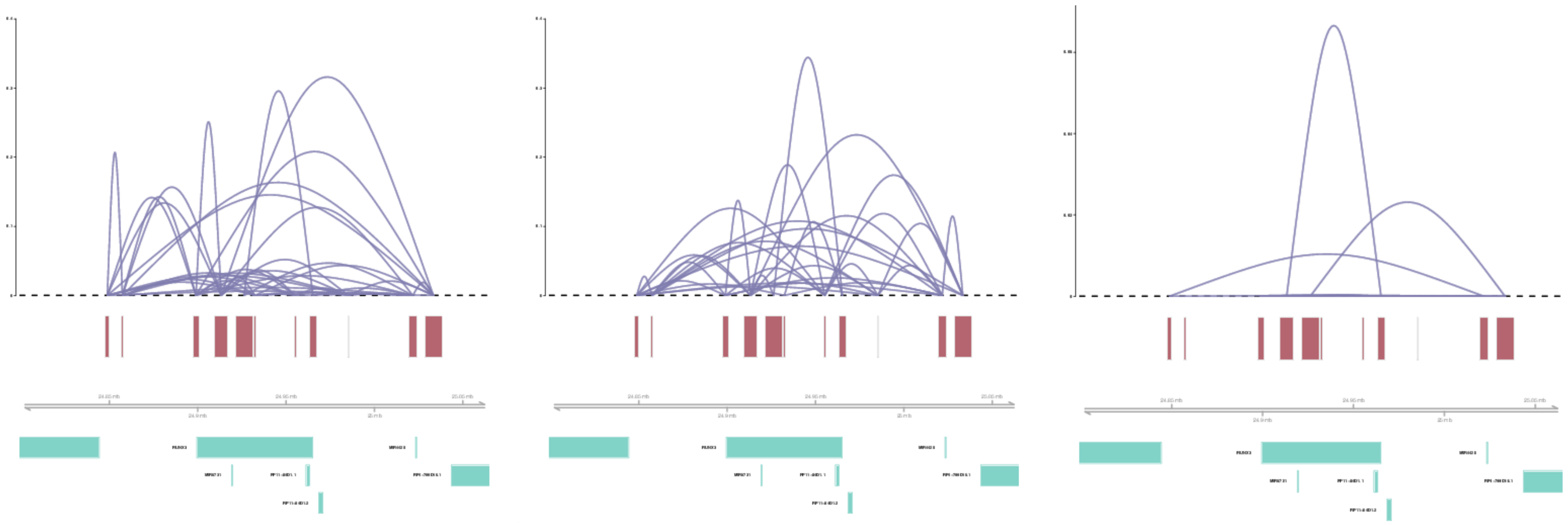

FIGURE S5 (I). The reconstruction of genome-wide cis-regulatory interaction networks for certain regions across specific genes for all cell types among three groups in CD8$^+$ T cells.

JUN CD8^{+} T cells

HCs MPs SCPs

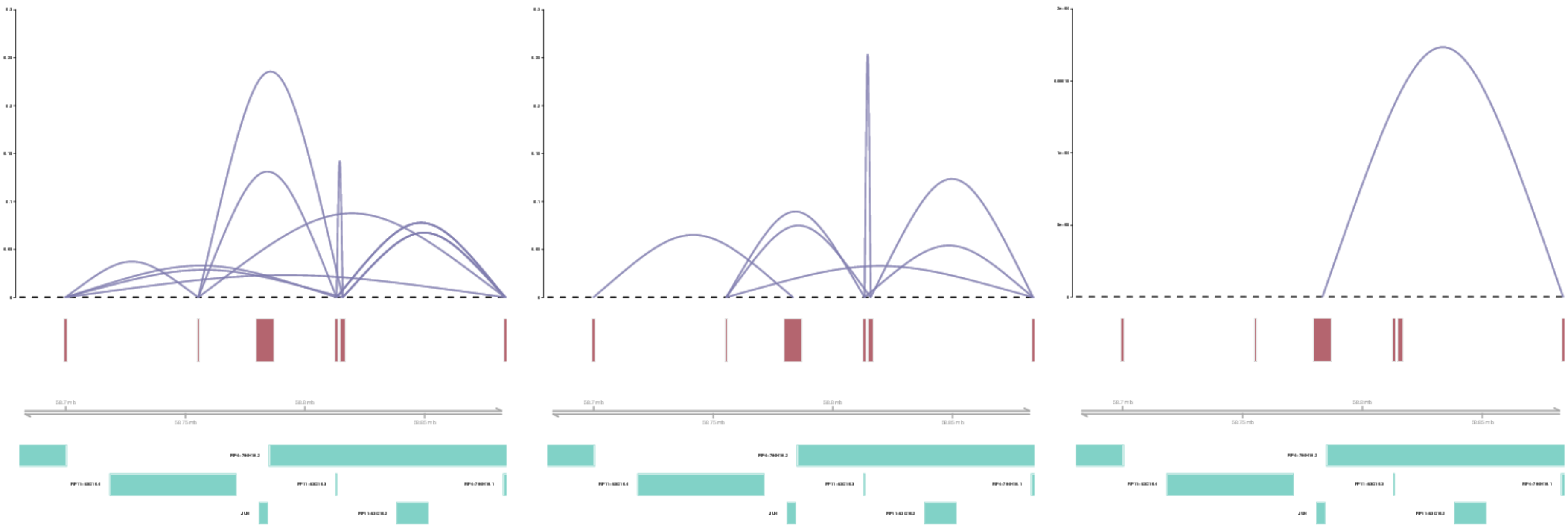

FIGURE S5 (J). The reconstruction of genome-wide cis-regulatory interaction networks for certain regions across specific genes for all cell types among three groups in CD8^{+} T cells.

HCs

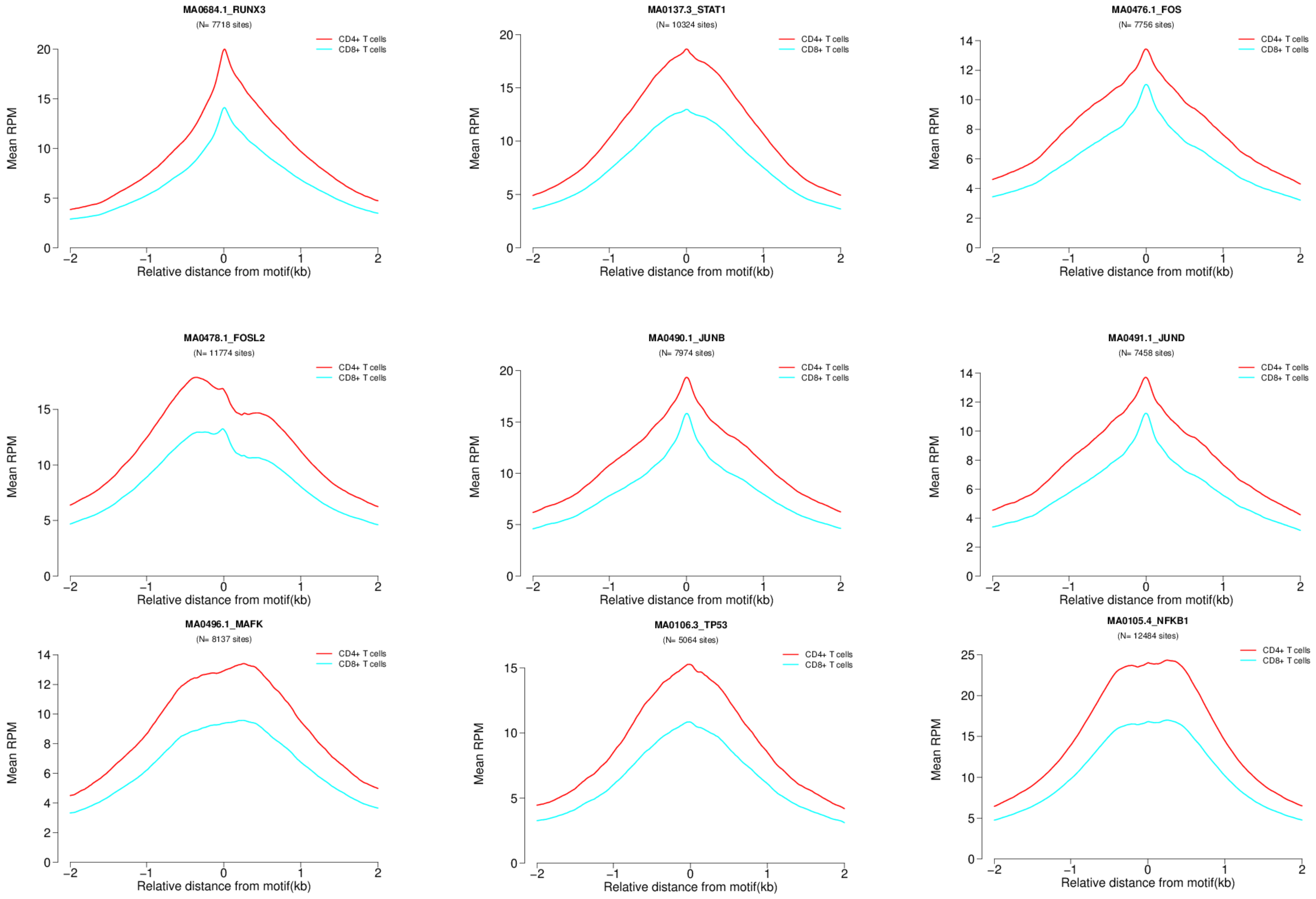

FIGURE S6 (A). Aggregate read counts in 4kb windows centered on each identified motif instance is shown for HCs.

MPs

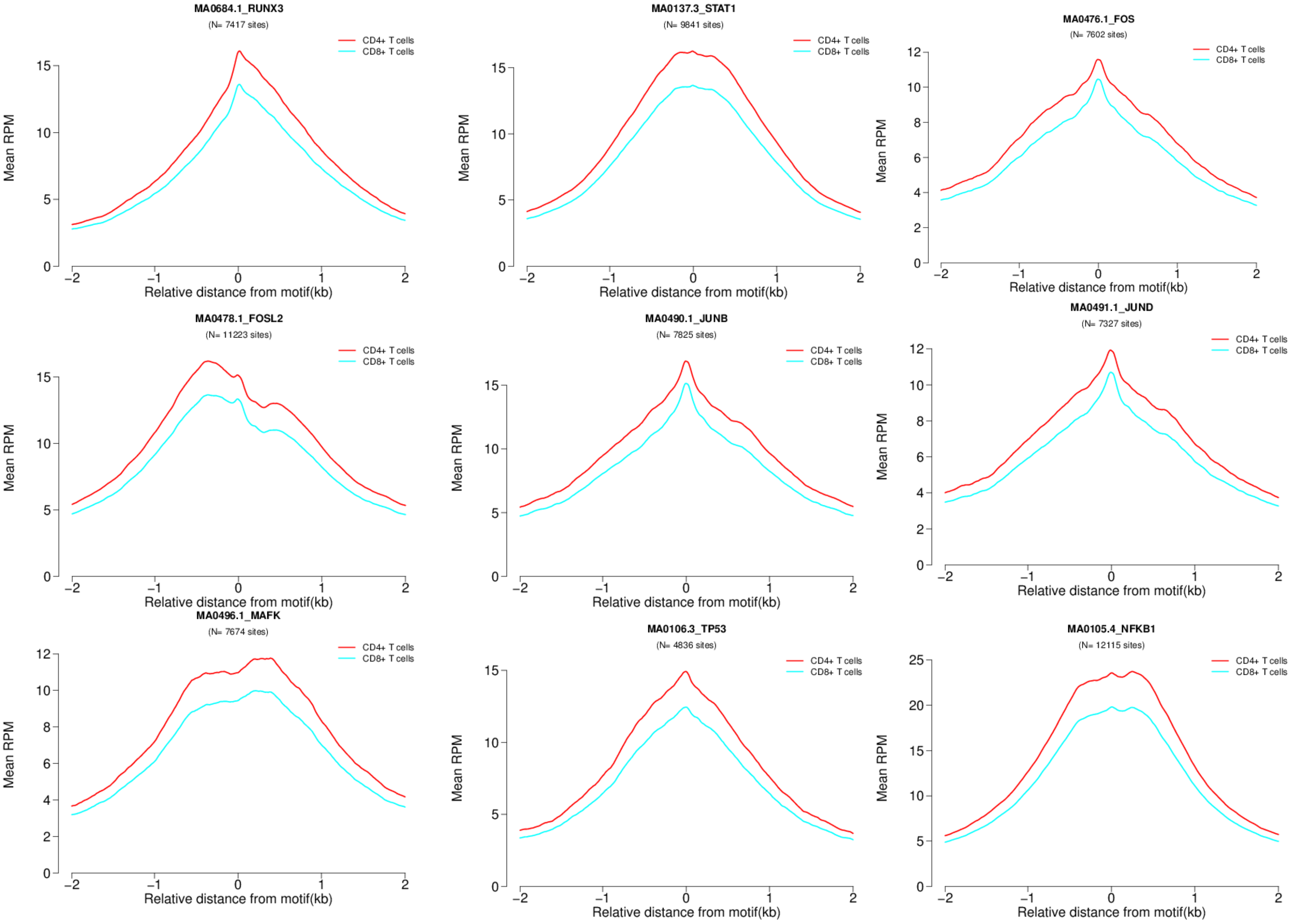

FIGURE S6 (B). Aggregate read counts in 4kb windows centered on each identified motif instance is shown for MPs.

SCPs

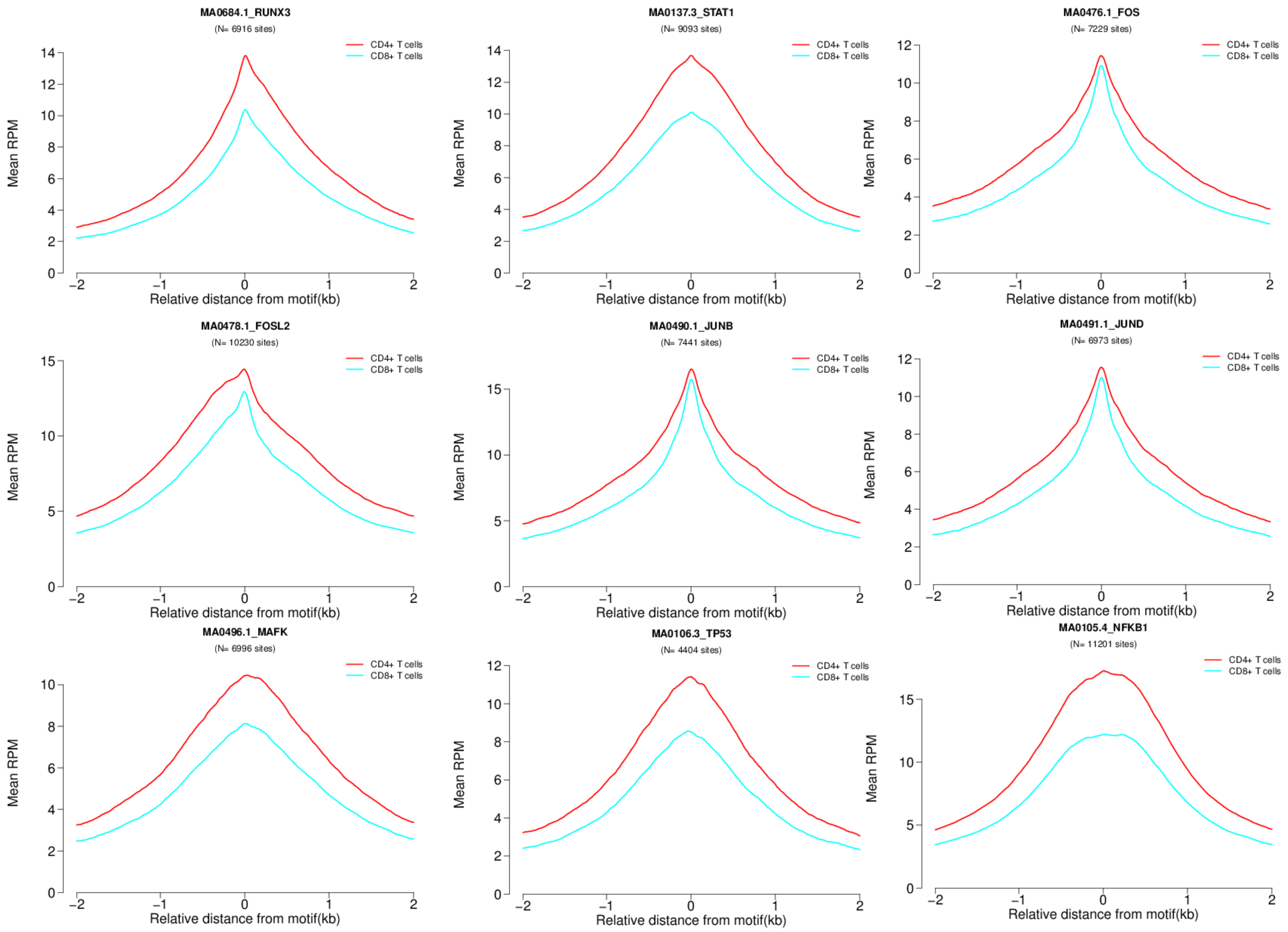

FIGURE S6 (C). Aggregate read counts in 4kb windows centered on each identified motif instance is shown for severe/critical cases.

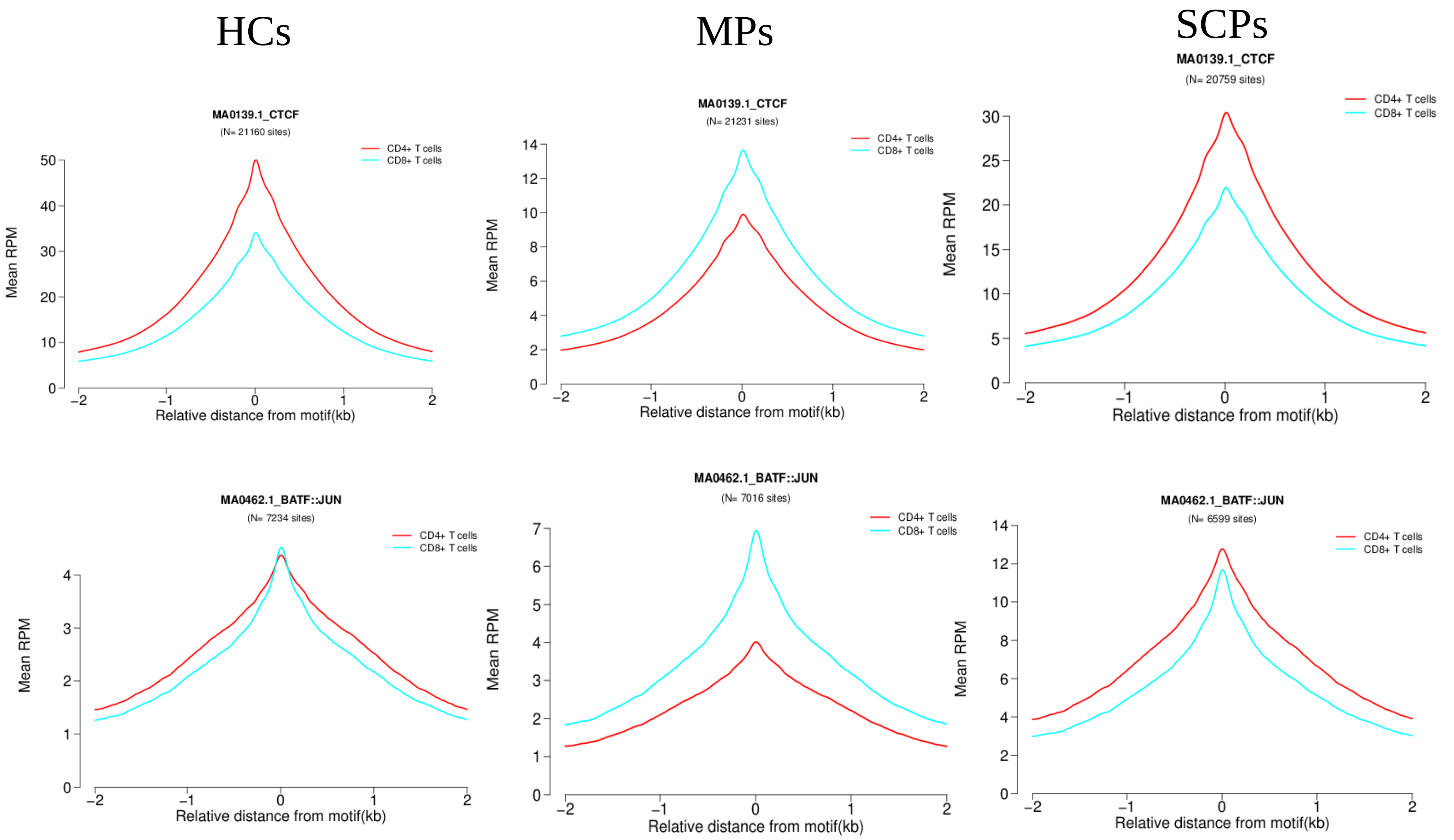

FIGURE S6 (D). Aggregate read counts in 4kb windows centered on CTCF and BATF::JUN motif instance is shown for HCs, MPs and SCPs.

T cells

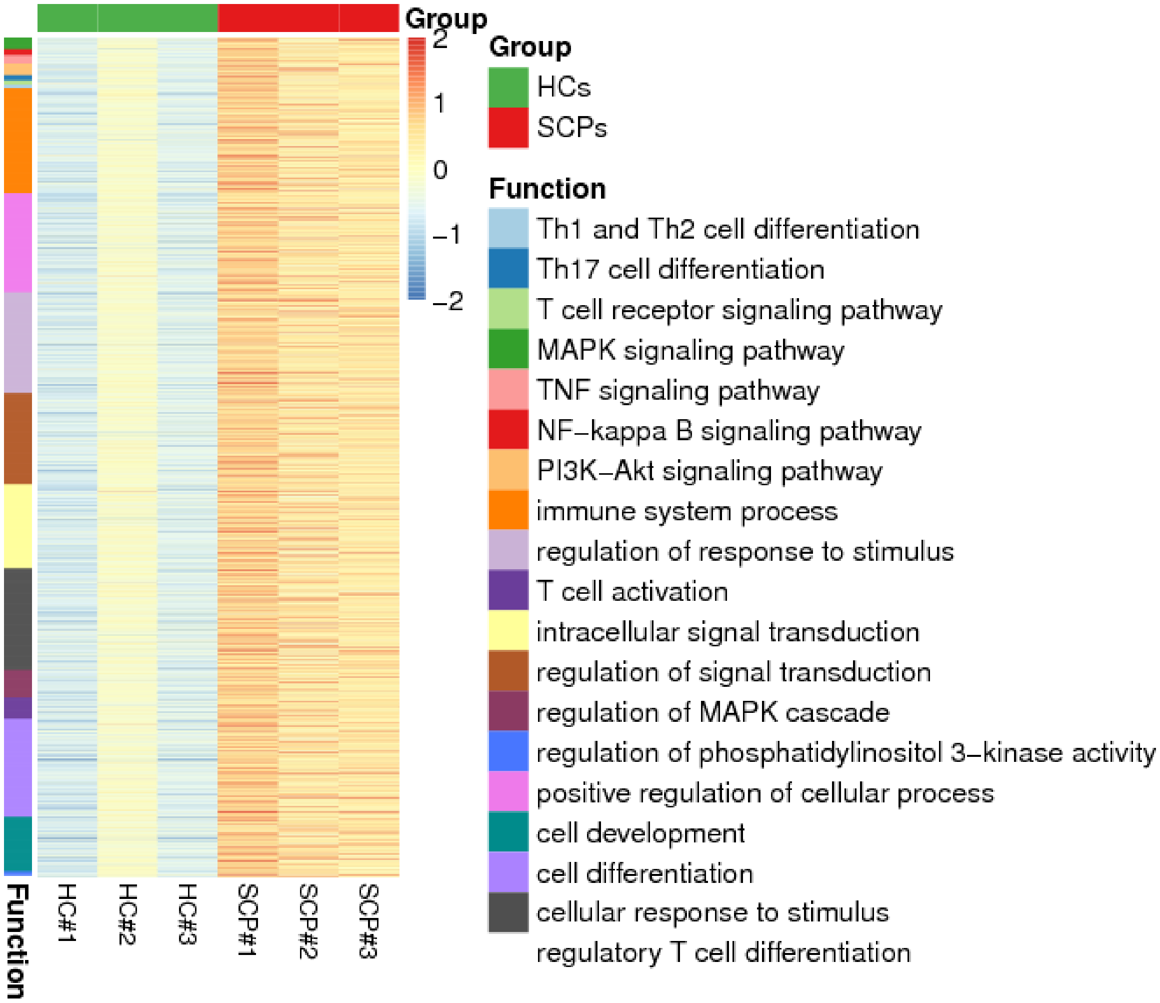

FIGURE S7 (A). The heatmap of up-regulated GO and KEGG enrichments for the T cells of HCs vs. SCPs by scATAC-seq.

T cells

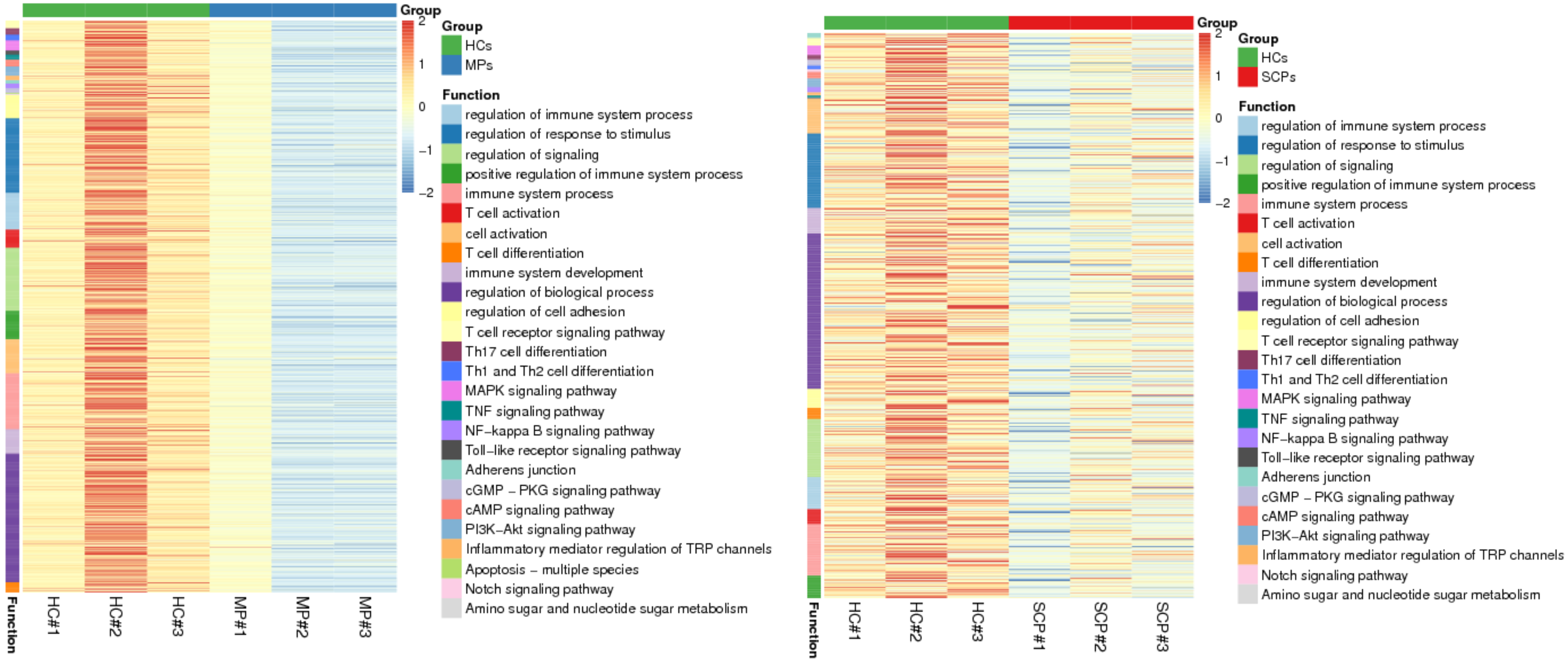

FIGURE S7 (B). The heatmap of down-regulated GO and KEGG enrichments for the T cells of HCs vs. MPs and HCs vs. SCPs by scATAC-seq.

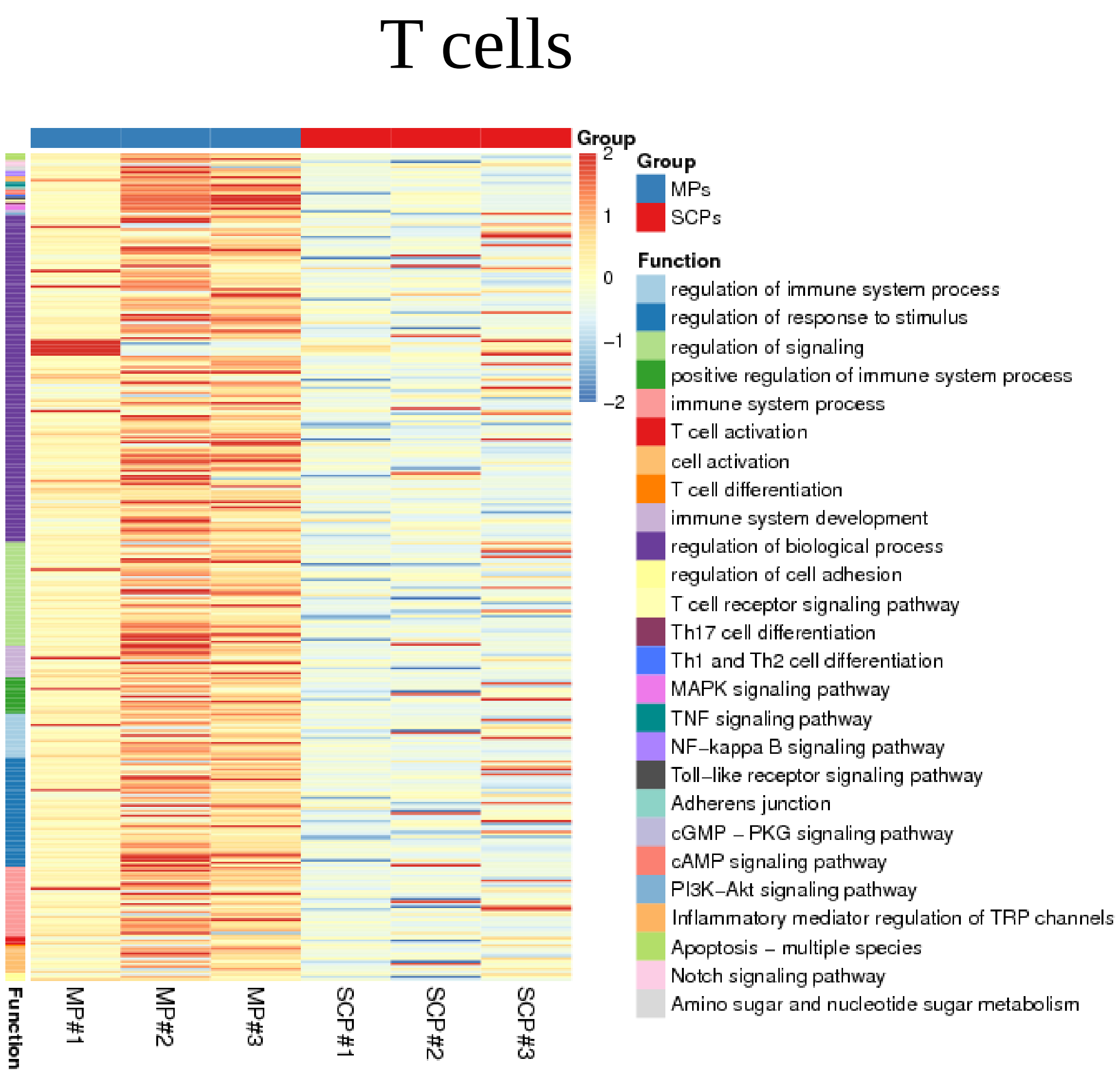

FIGURE S7 (C). The heatmap of down-regulated GO and KEGG enrichments for the T cells of MPs vs. SCPs by scATAC-seq.

CD4⁺ T cells

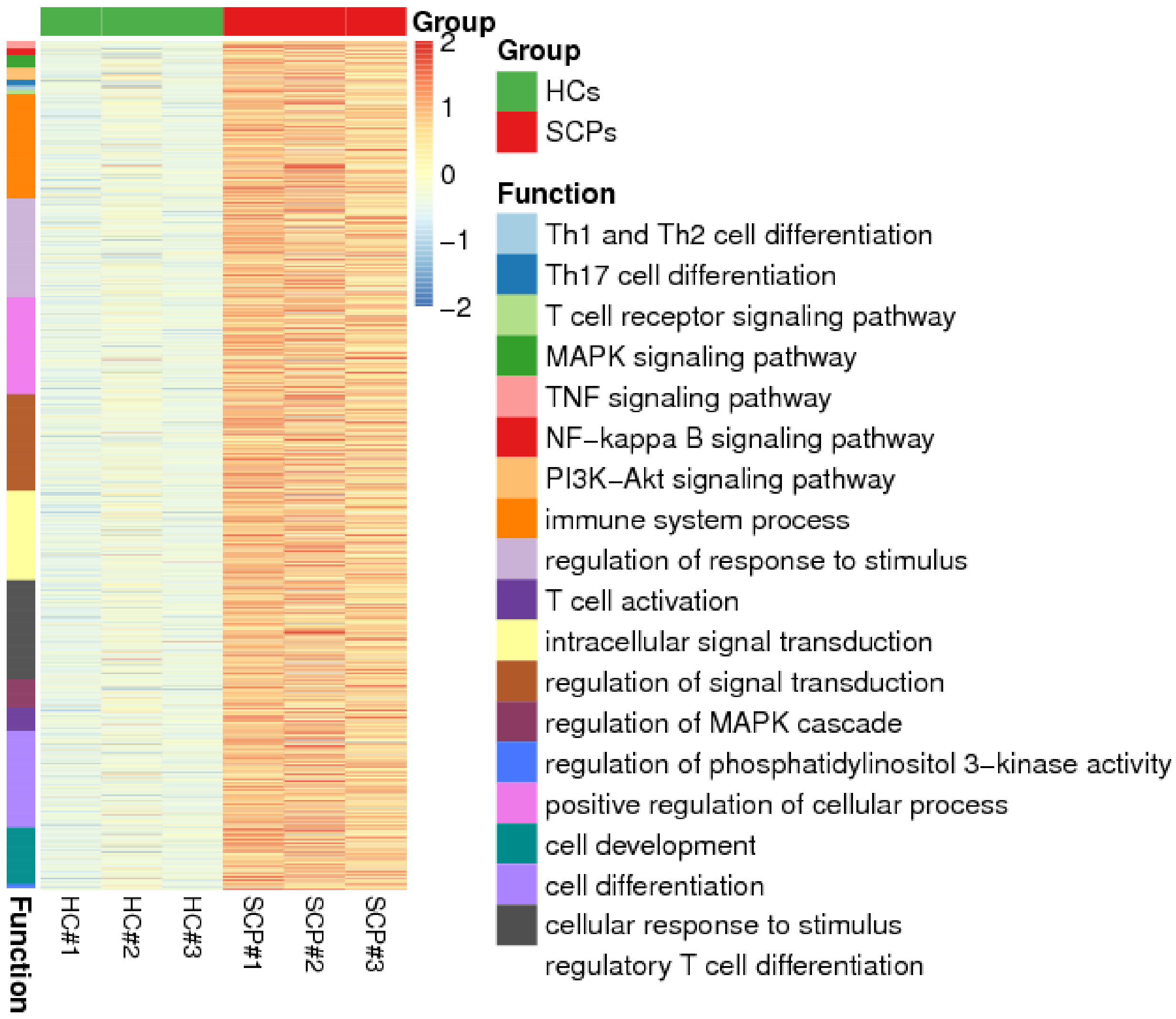

FIGURE S7 (D). The heatmap of up-regulated GO and KEGG enrichments for the CD4$^+$ T cells of HCs vs. SCPs by scATAC-seq.

CD4$^+$ T cells

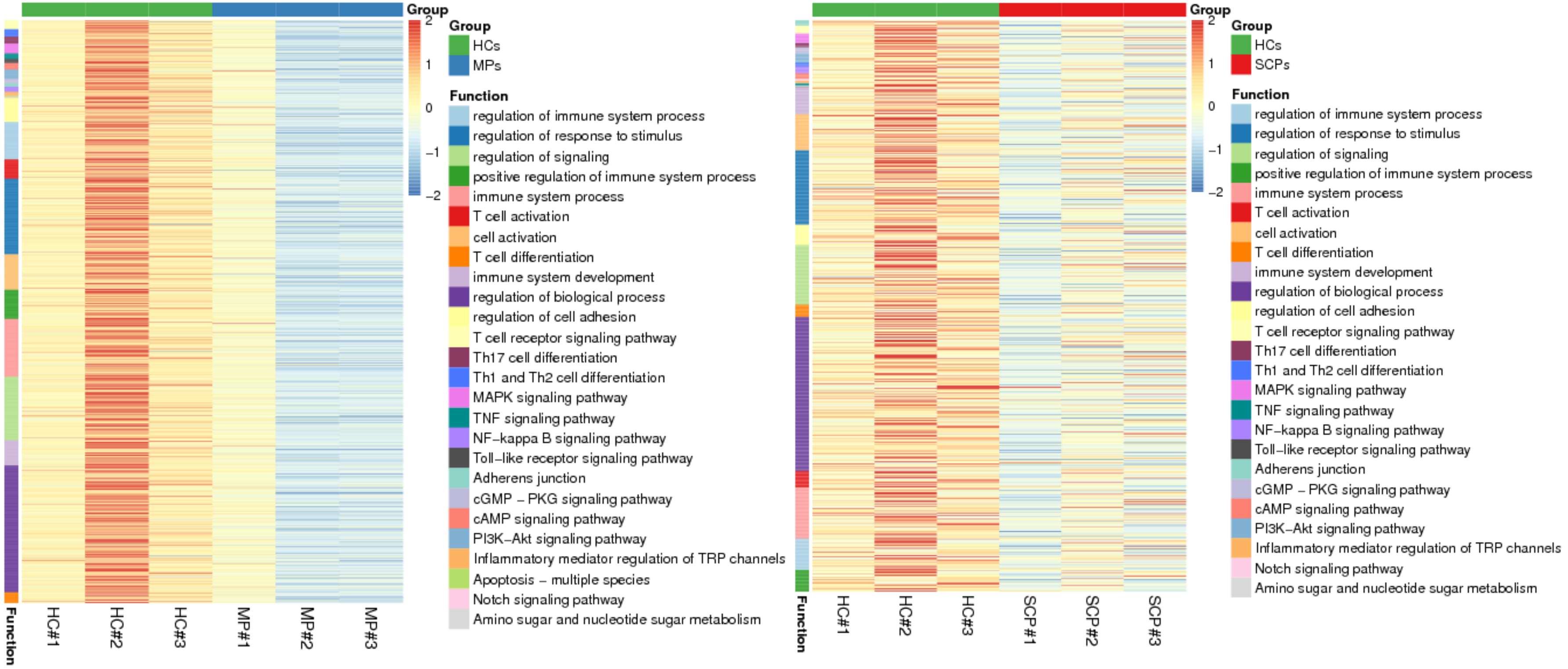

FIGURE S7 (E). The heatmap of down-regulated GO and KEGG enrichments for the CD4$^+$ T cells of HCs vs. MPs and HCs vs. SCPs by scATAC-seq.

CD4$^+$ T cells

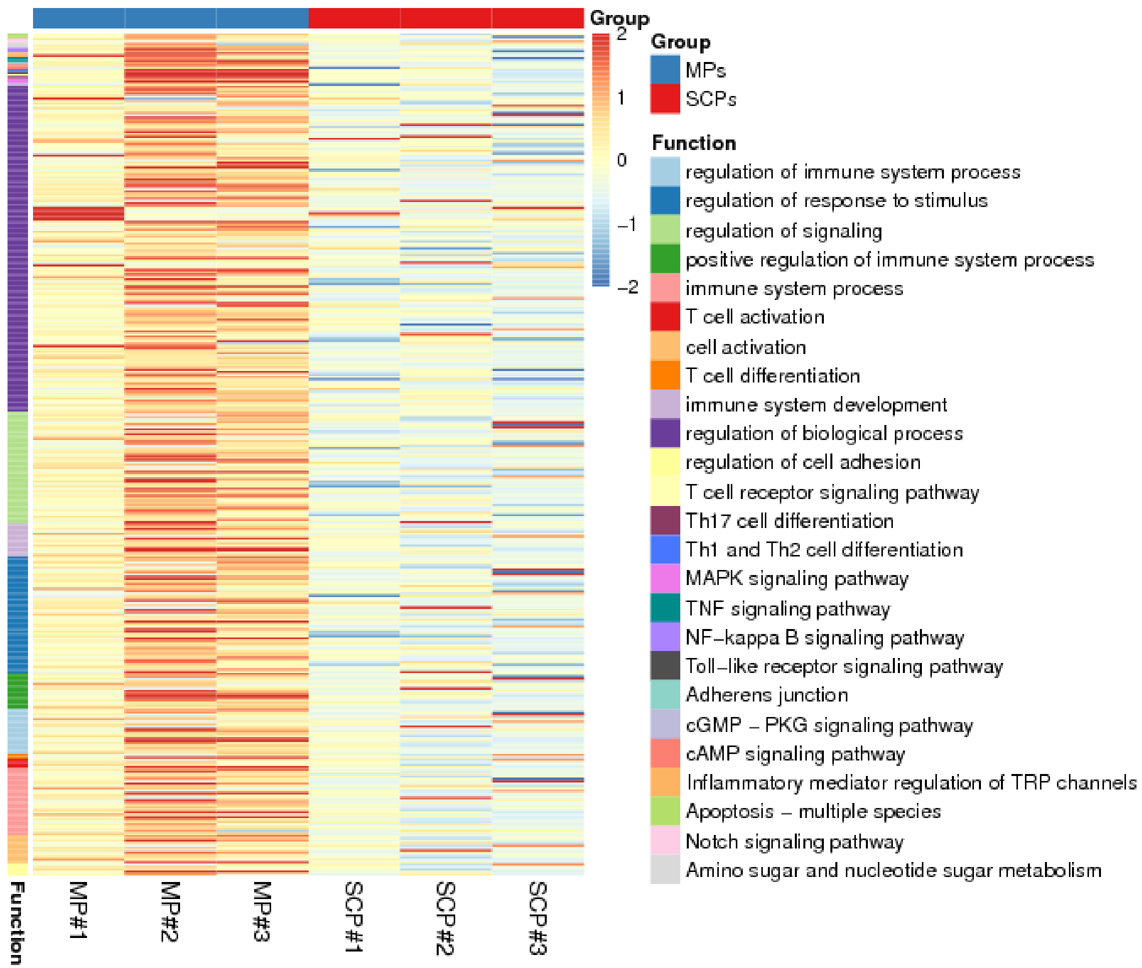

FIGURE S7 (F). The heatmap of down-regulated GO and KEGG enrichments for the CD4$^+$ T cells of MPs vs. SCPs by scATAC-seq.

CD8+ T cells

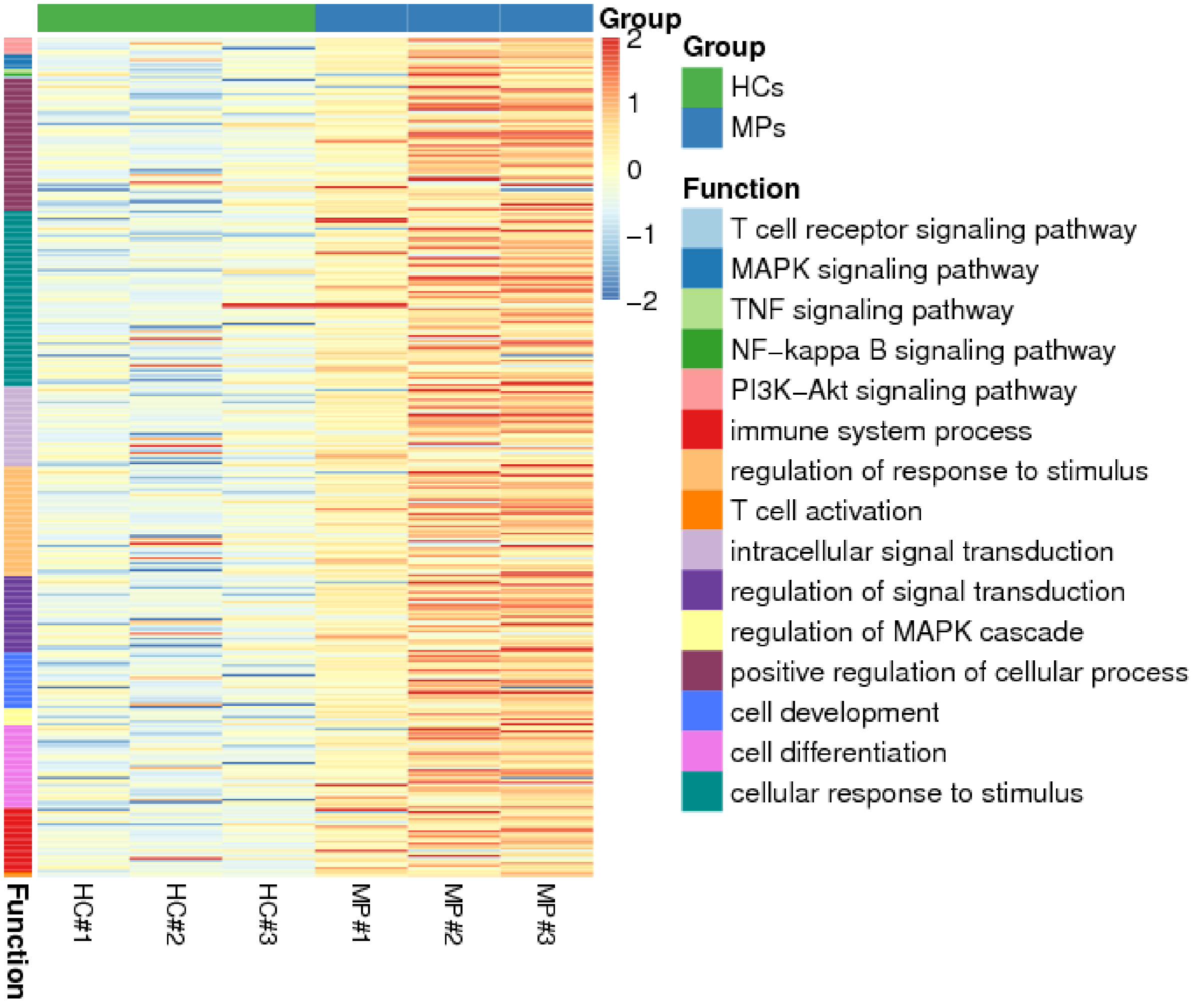

FIGURE S7 (G). The heatmap of up-regulated GO and KEGG enrichments for the CD8+ T cells of HCs vs. MPs by scATAC-seq.

CD8^{+} T cells

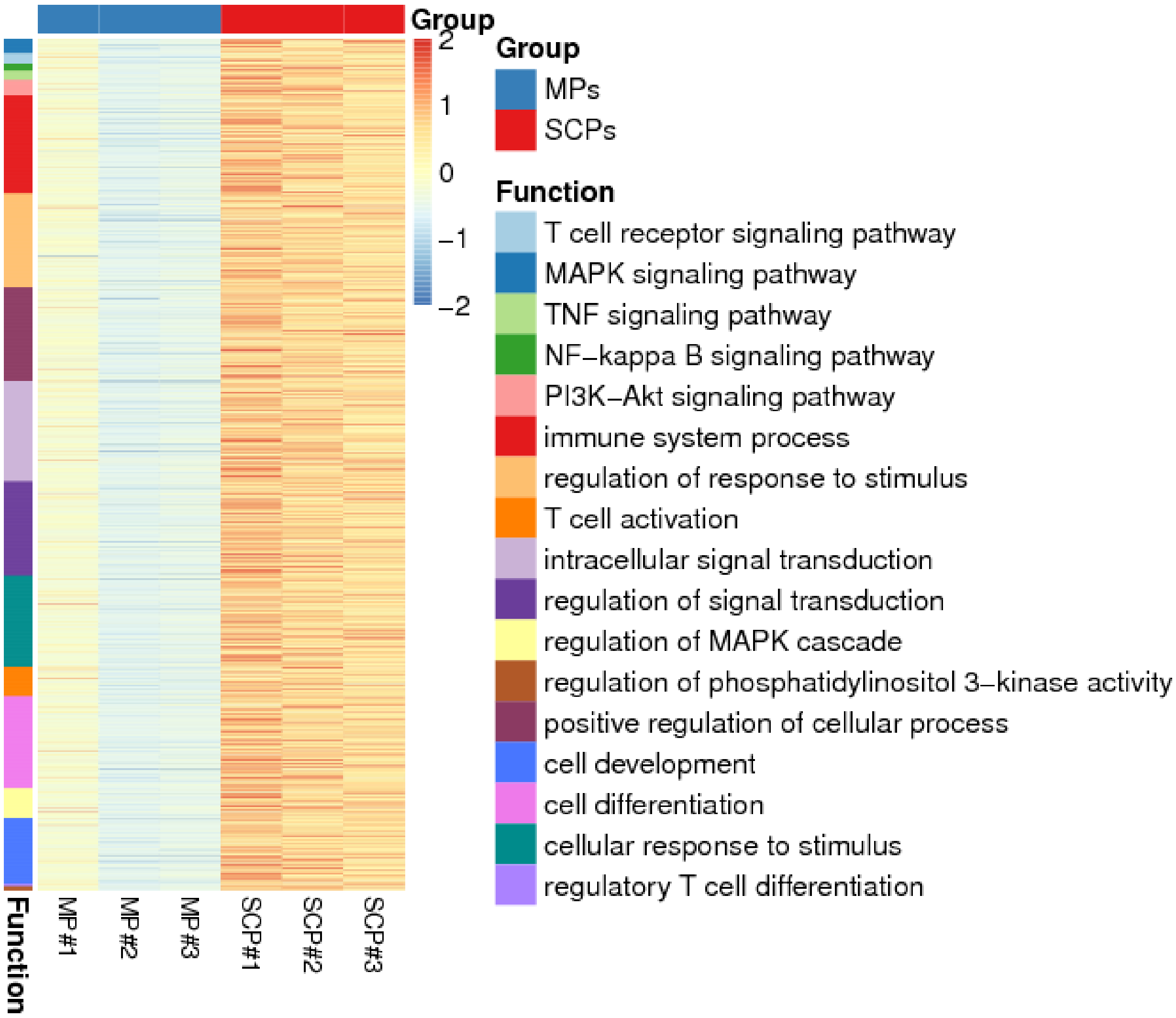

FIGURE S7 (H). The heatmap of up-regulated GO and KEGG enrichments for the CD8^{+} T cells of MPs vs. SCPs by scATAC-seq.

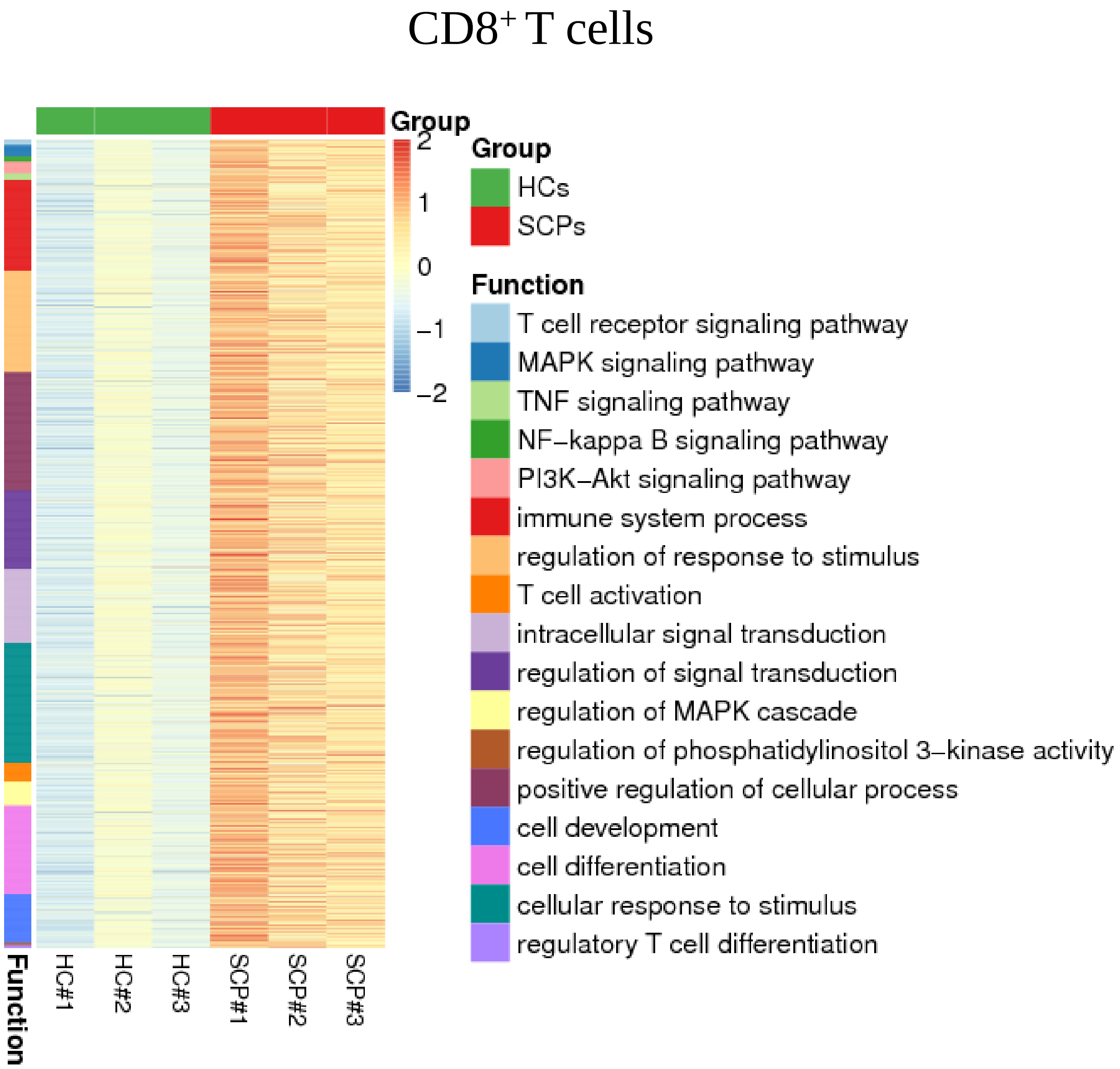

FIGURE S7 (I). The heatmap of up-regulated GO and KEGG enrichments for the CD8+ T cells of HCs vs. SCPs by scATAC-seq.

CD8+ T cells

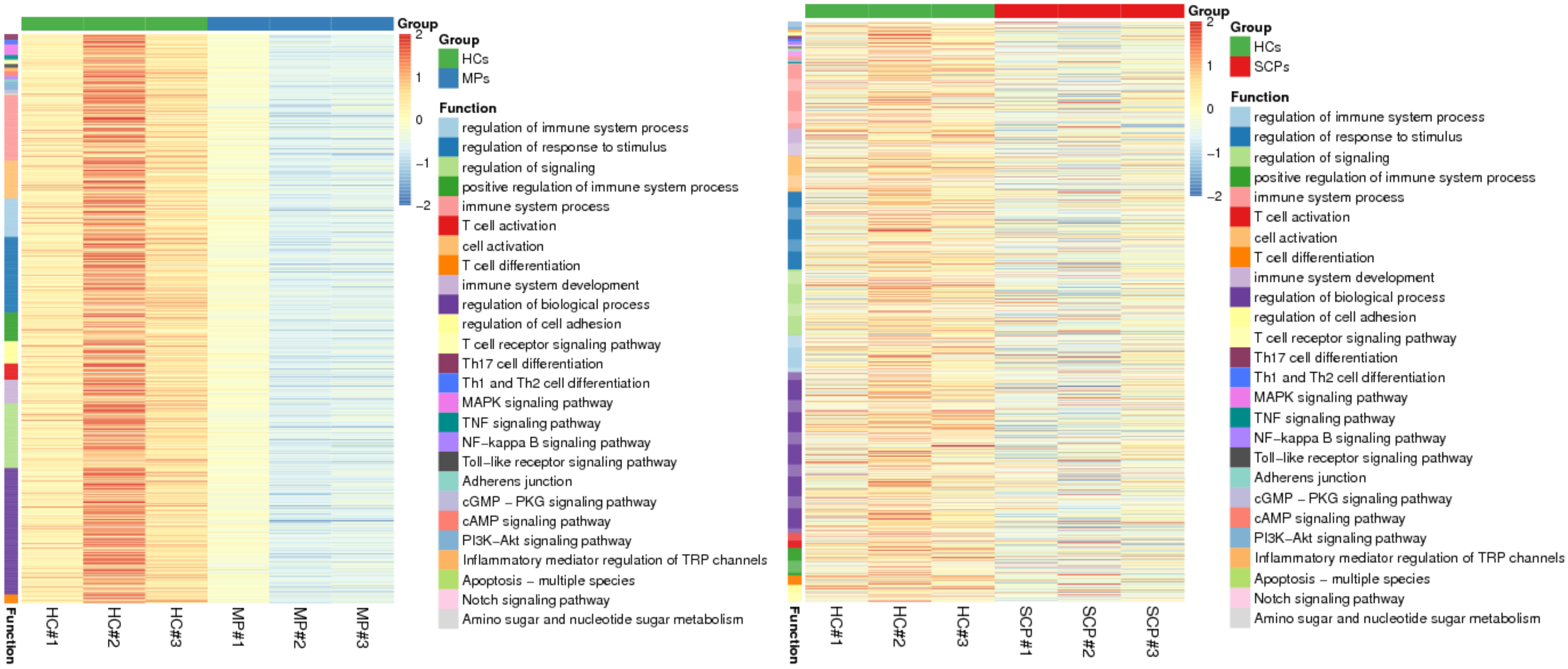

FIGURE S7 (J). The heatmap of down-regulated GO and KEGG enrichments for the CD8+ T cells of HCs vs. MPs and HCs vs. SCPs by scATAC-seq.

CD8^{+} T cells

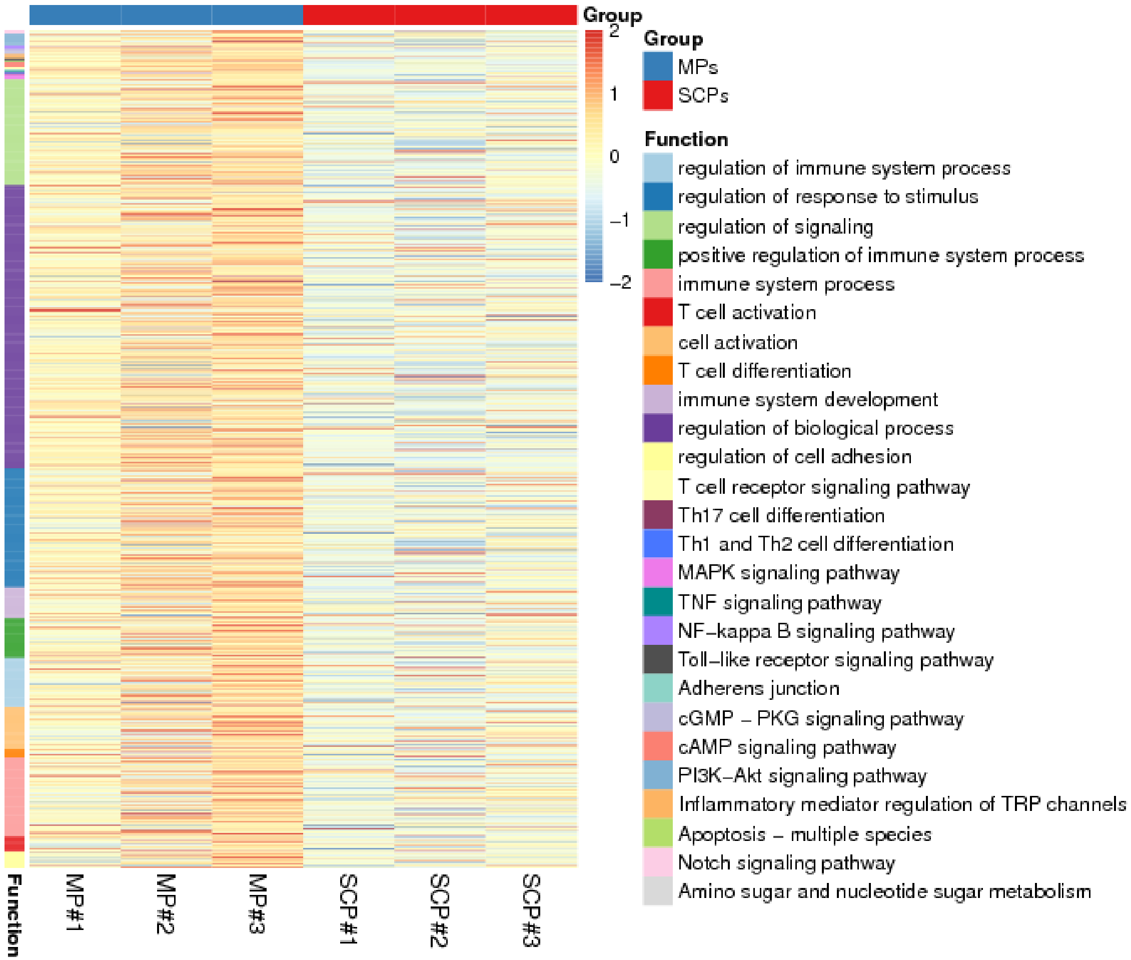

FIGURE S7 (K). The heatmap of down-regulated GO and KEGG enrichments for the CD8^{+} T cells of MPs vs. SCPs by scATAC-seq.

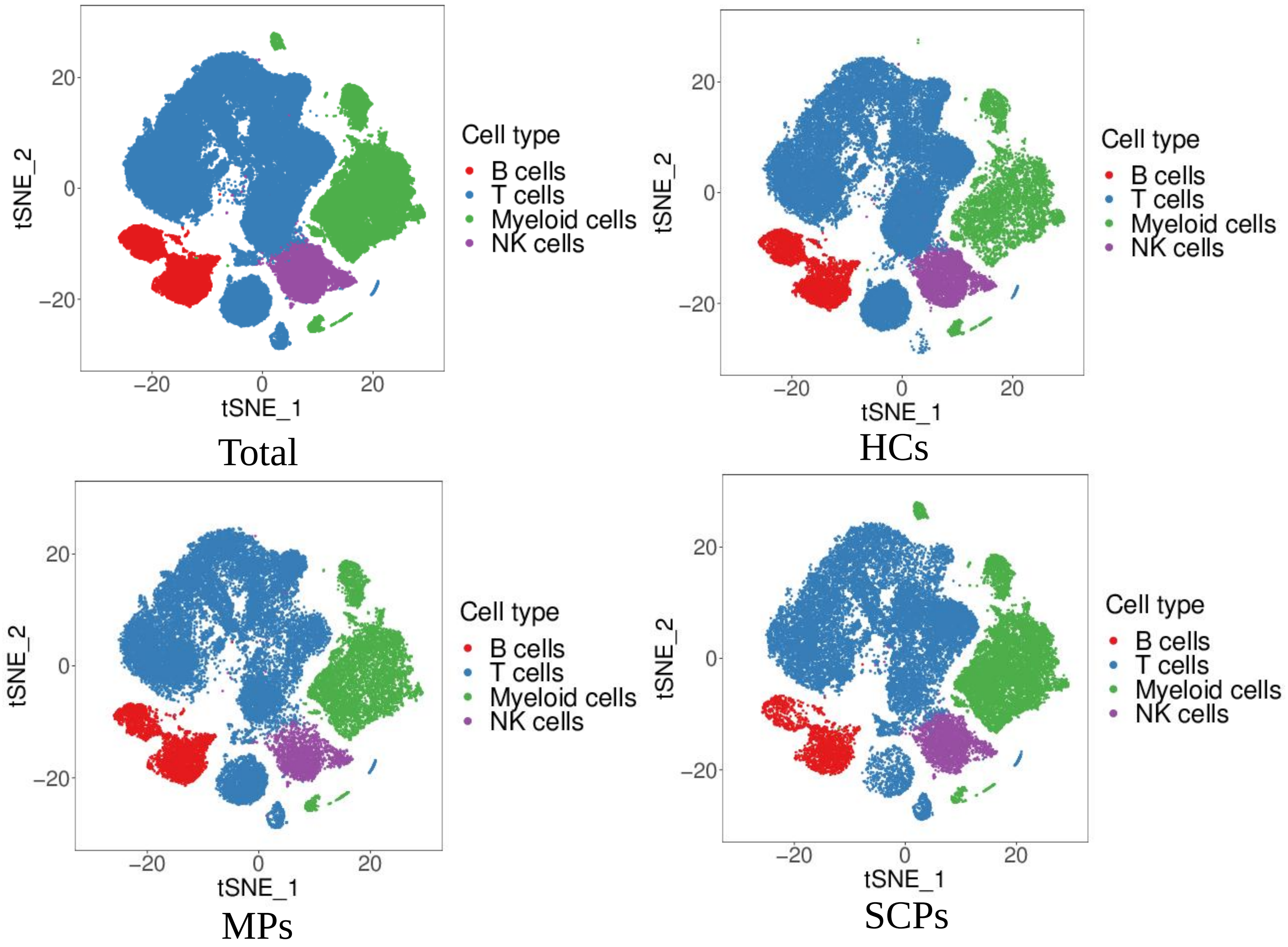

FIGURE S8 (A) An overview of T, B, NK and Myeloid cells in the PBMC of COVID-19 patients. The t-SNE pot shows a comparison of the clustering distribution of each groups.

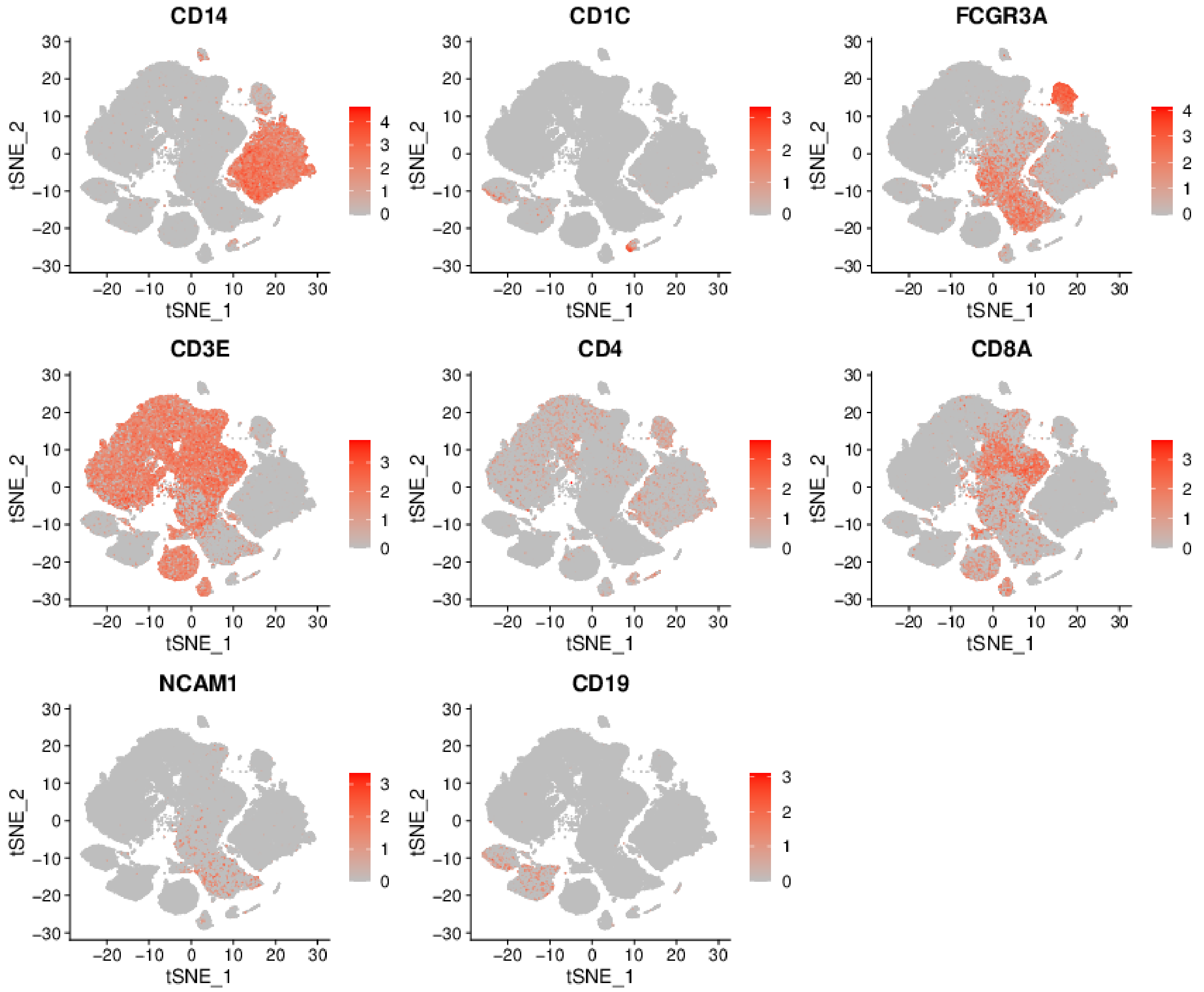

FIGURE S8. (B) t-SNE projection of canonical markers, including CD14, CD1C and FCGR3A for myeloid cells;CD3E, CD4 and CD8A for T cells; NCAM1 for NK cells; and CD19 for B cells.

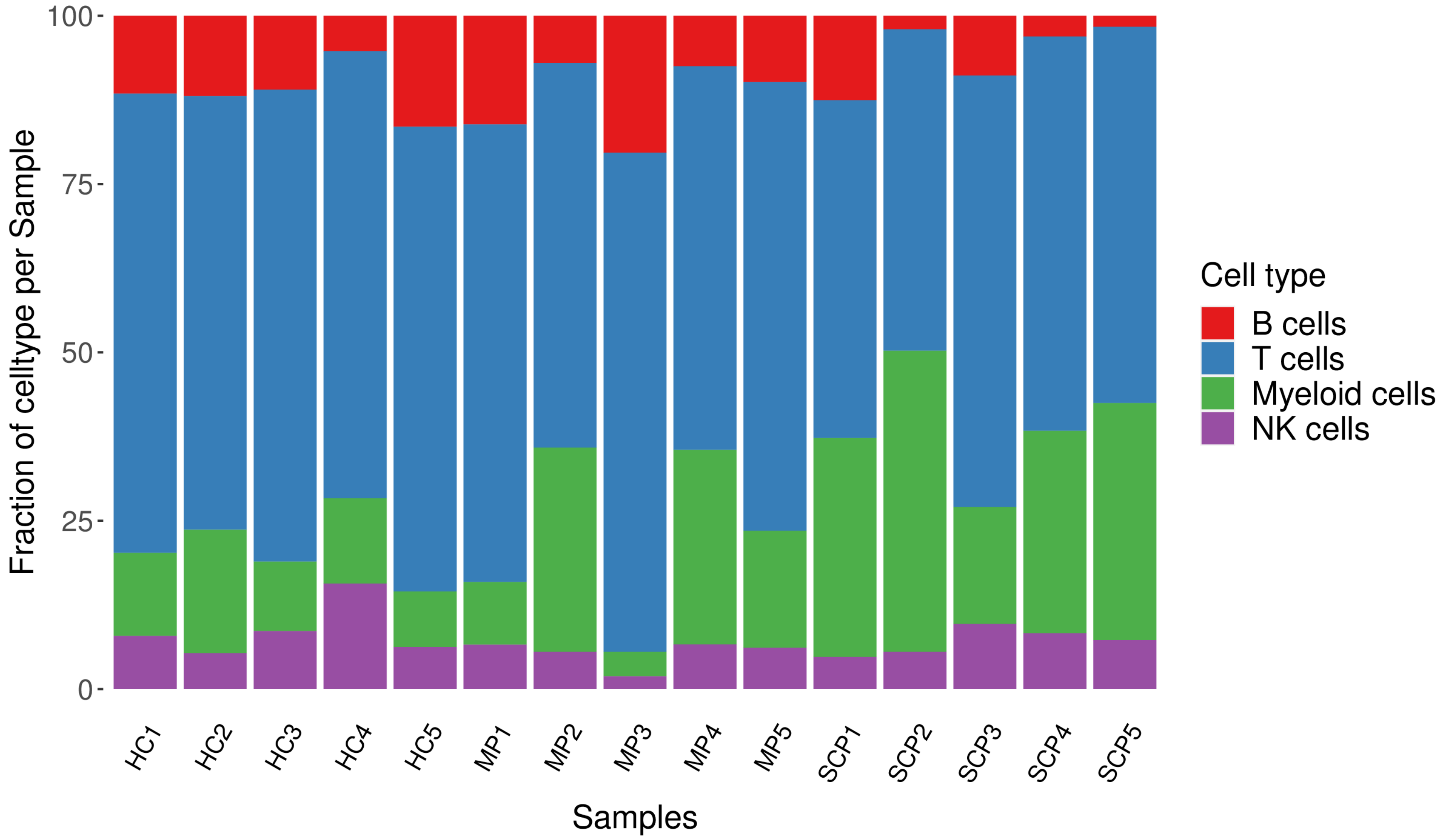

FIGURE S8. (C) The bar plot shows the relative contributions of B, T, Myeloid and NK cells by individual samples, including five health volunteers, five MPs and five severe/critical cases.

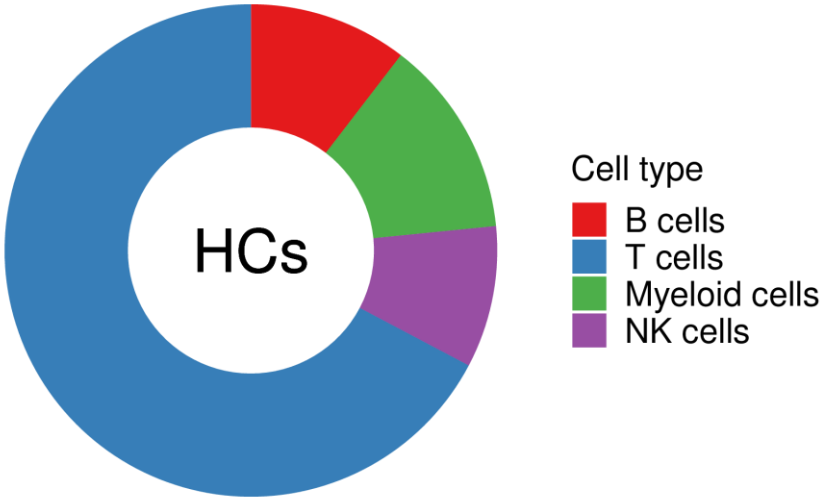

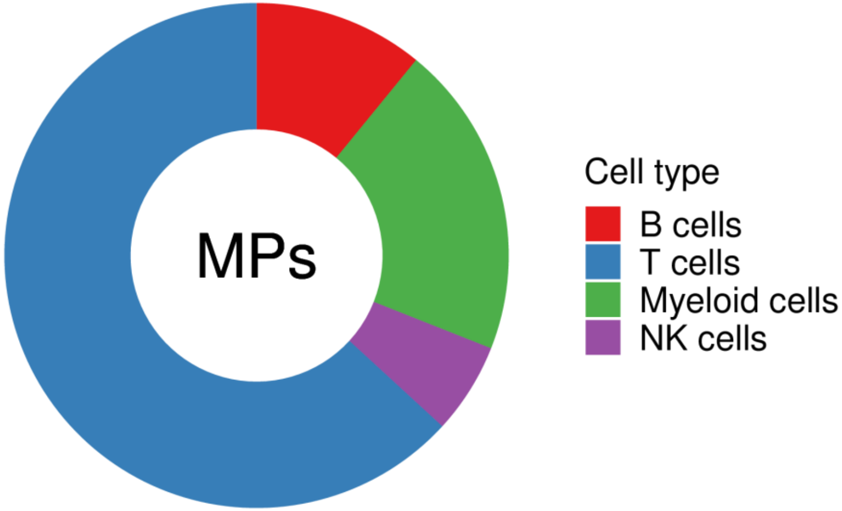

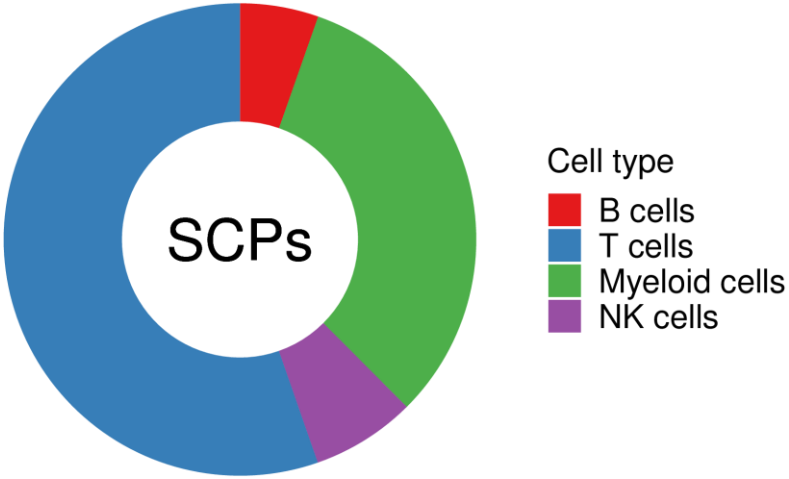

FIGURE S8. (D) The pie chats show the percentage of B, T, Myeloid and NK cells from the group of HCs, MPs and SCPs.

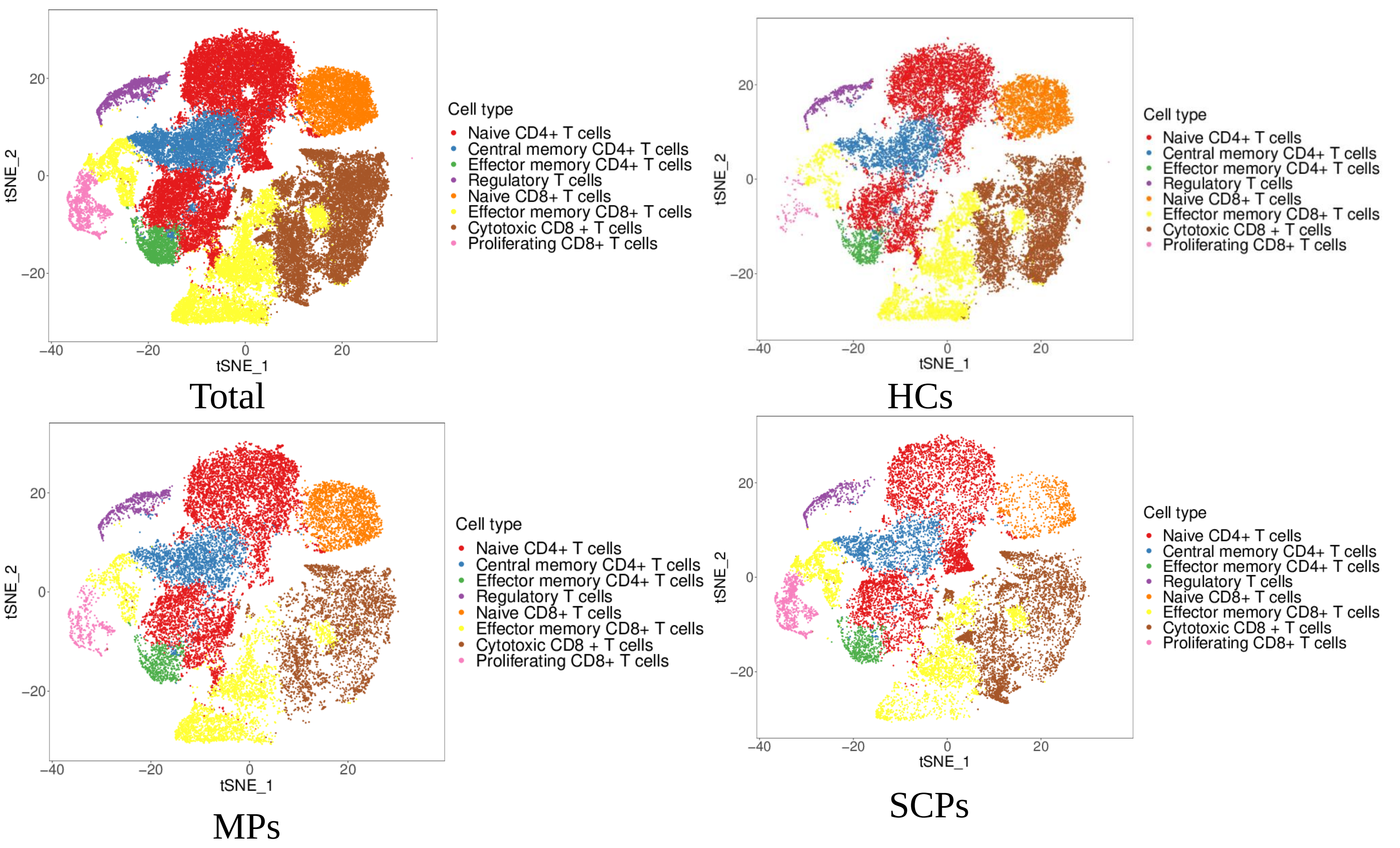

FIGURE S8. (E) Characterization of T cells in the PBMC of COVID-19 patients. The t-SNE plot shows the clustering of T cells.

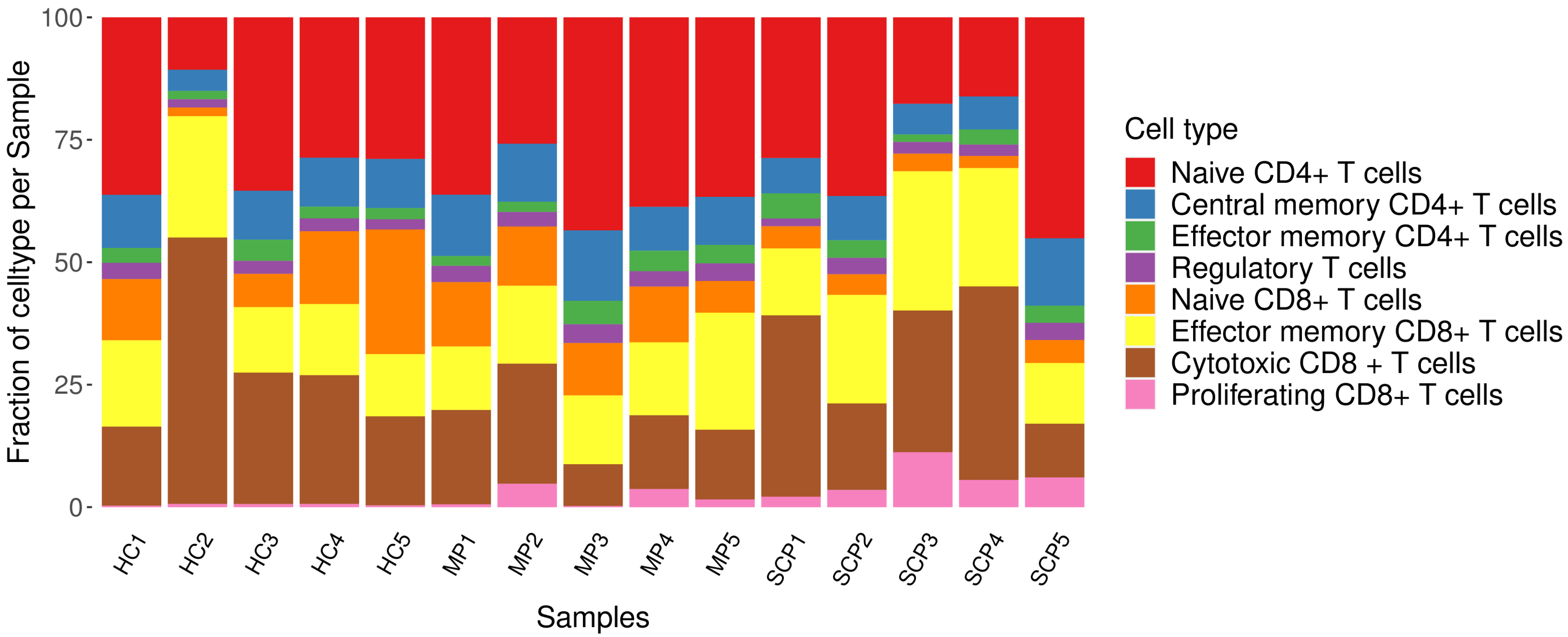

FIGURE S8. (F) The bar plot shows the relative contributions of na ïve $CD4^+$ T cells, central memory $CD4^+$ T cells, effector/effector memory $CD4^+$ T cells, regulatory T cells, na ïve $CD8^+$ T cells, effector memory $CD8^+$ T cells, effector/cytotoxic $CD8^+$ T cells and proliferating T cells by individual samples, including five health volunteers, five MPs and five severe/critical cases.

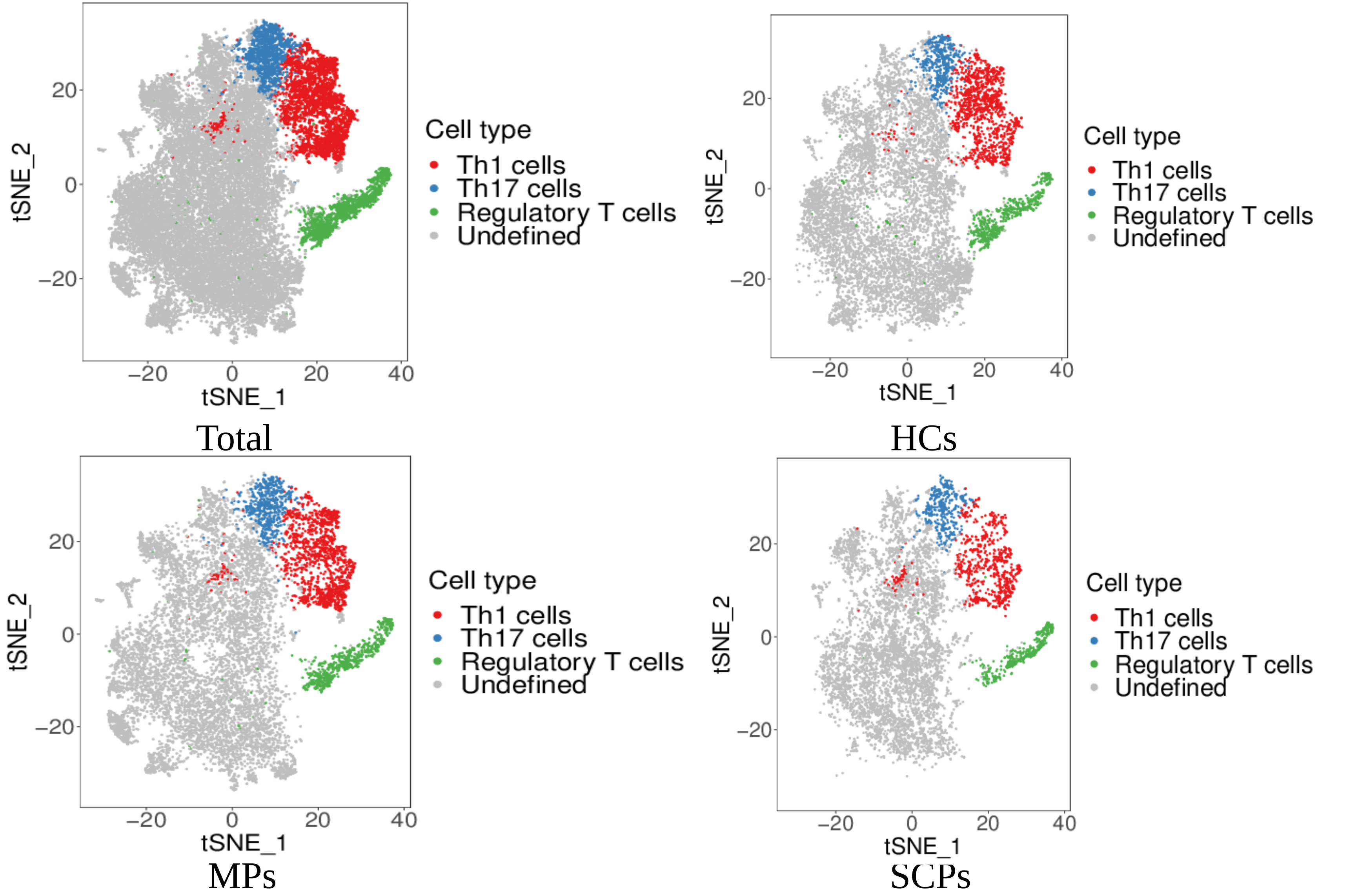

FIGURE S8. (G) Characterization of CD4^{+} T cells in the PBMC of COVID-19 patients. The t-SNE plot show the clustering of Th1, Th17 and Tfh cells.

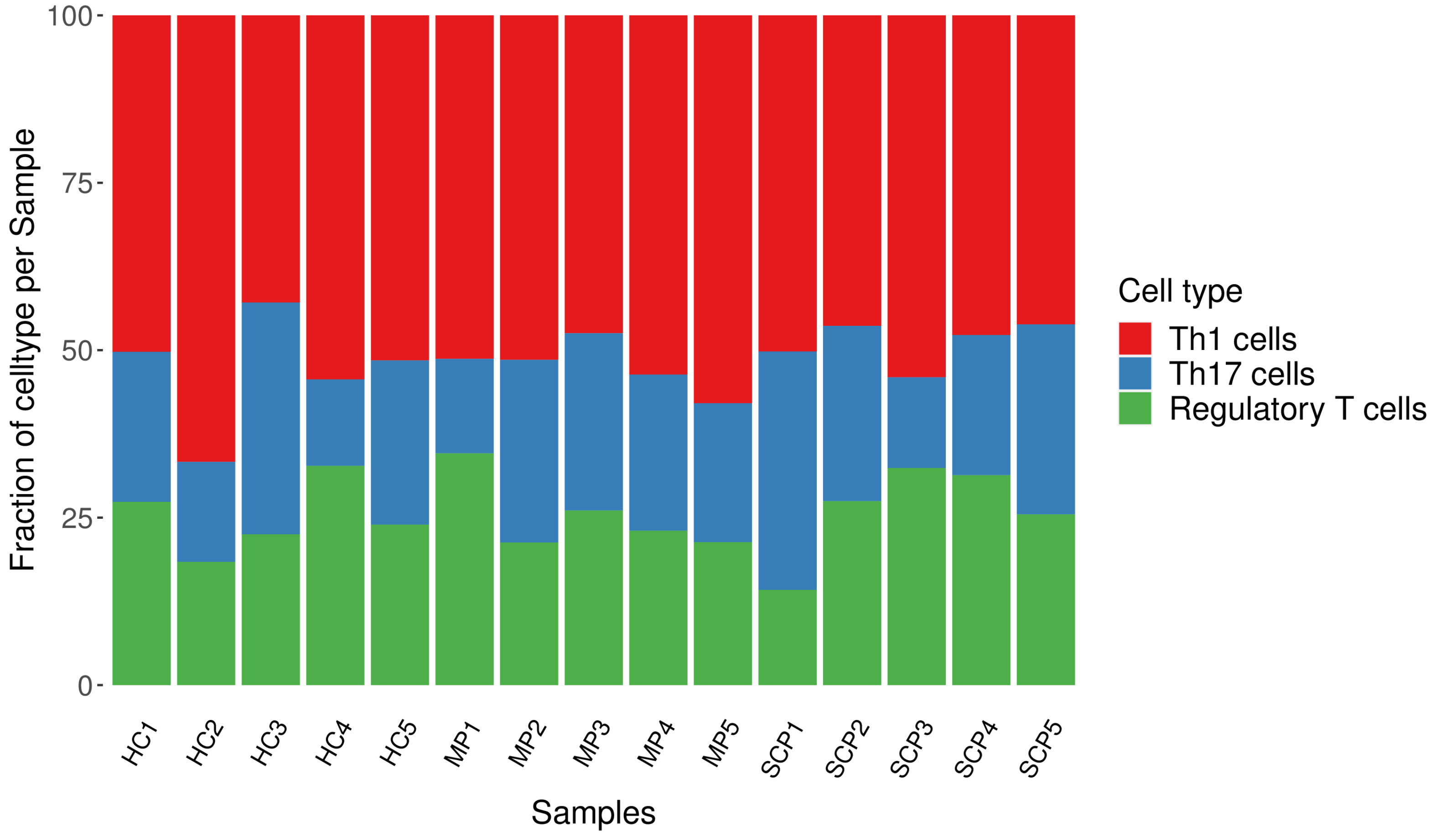

FIGURE S8. (H) The bar plot shows the relative contributions of Th1, Th17 and regulatory T cells by individual samples, including five health volunteers, five MPs and five severe/critical cases.

T cells

HCs vs. SCPs

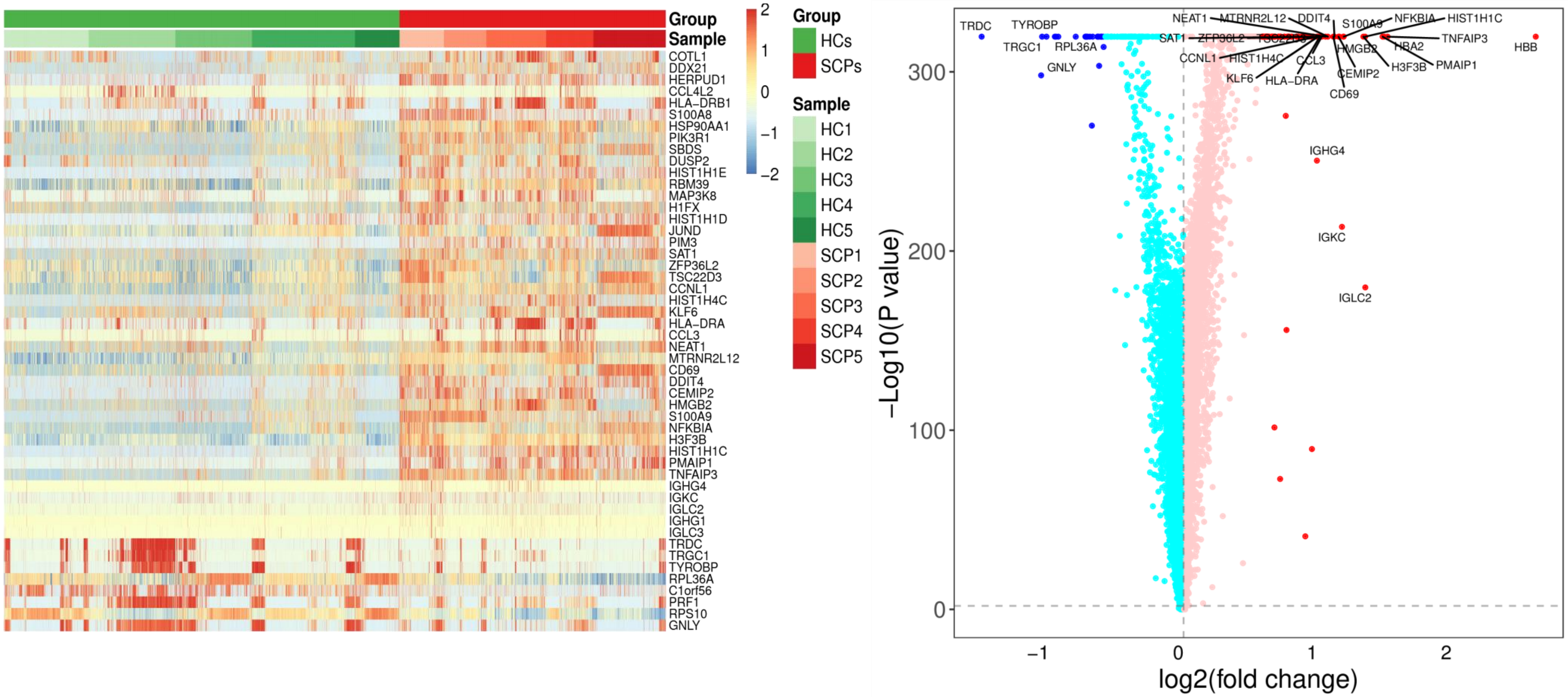

FIGURE S9. (A) The heatmap shows the top DEGs between SCPs and HCs in T cells. Volcano plot of fold change between MPs and SCPs in T cells. Findmarker in Seurat v3.1.2 was used to perform differential analysis and the bimod likelihood ratio statistical test was used. Significantly differential expression genes were defined by both p-value (less than and equal to 0.05) and fold change (greater than and equal to 1.5).

CD4^{+} T cells

HCs vs. SCPs

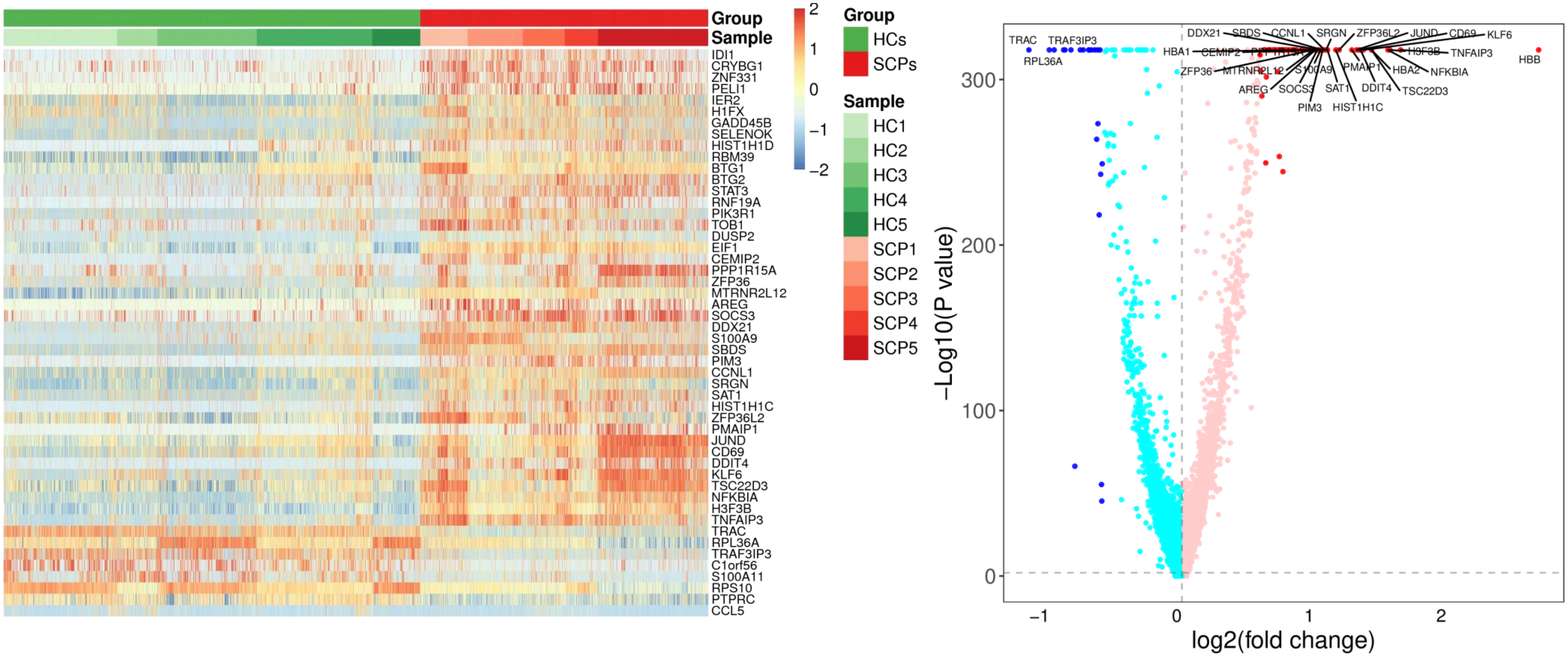

FIGURE S9. (B) The heatmap shows the top DEGs between SCPs and HCs in CD4^{+} T cells. Volcano plot of fold change between MPs and SCPs in CD4^{+} T cells. Findmarker in Seurat v3.1.2 was used to perform differential analysis and the bimod likelihood ratio statistical test was used. Significantly differential expression genes were defined by both p-value (less than and equal to 0.05) and fold change (greater than and equal to 1.5).

CD8$^+$ T cells

HCs vs. MPs

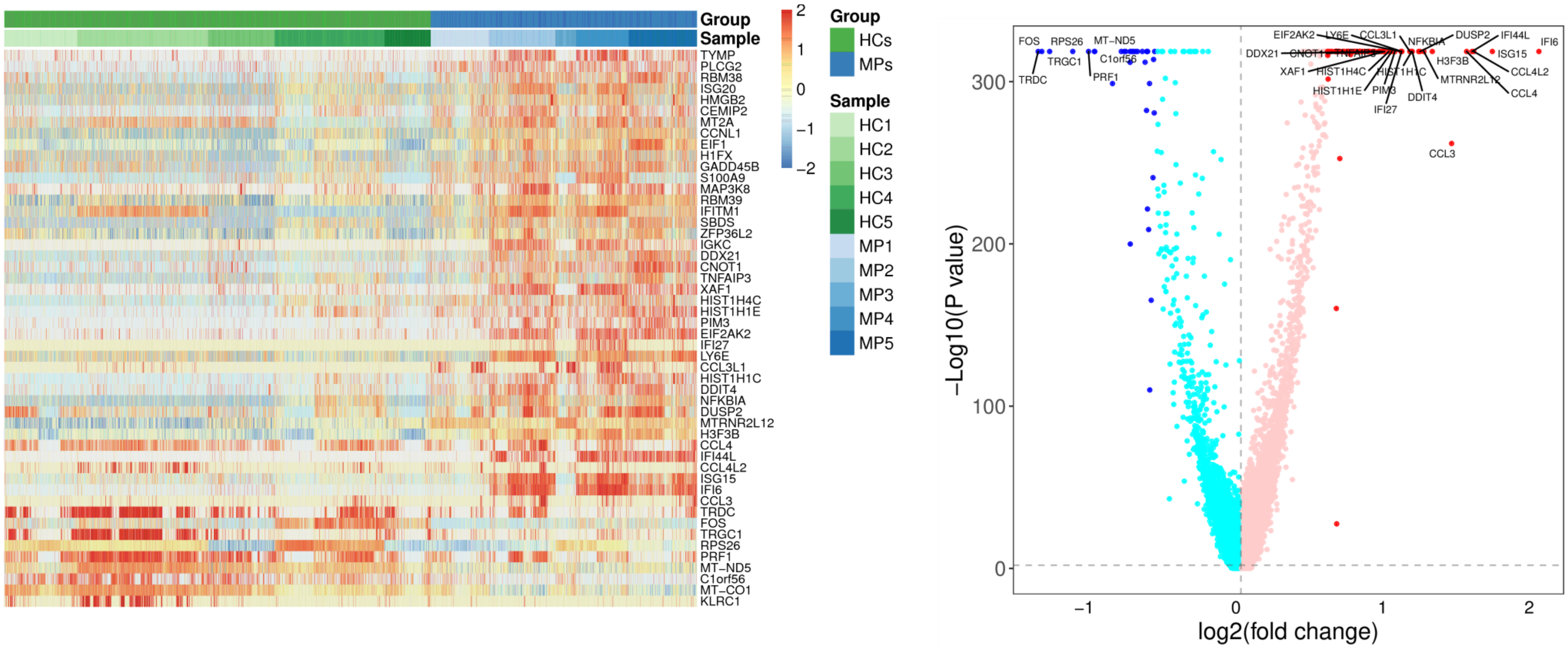

FIGURE S9. (C) The heatmap shows the top DEGs between HCs and MPs in CD8$^+$ T cells. Volcano plot of fold change between HCs and MPs in CD8$^+$ T cells. Findmarker in Seurat v3.1.2 was used to perform differential analysis and the bimod likelihood ratio statistical test was used. Significantly differential expression genes were defined by both p-value (less than and equal to 0.05) and fold change (greater than and equal to 1.5).

CD8^{+} T cells

MPs vs. SCPs

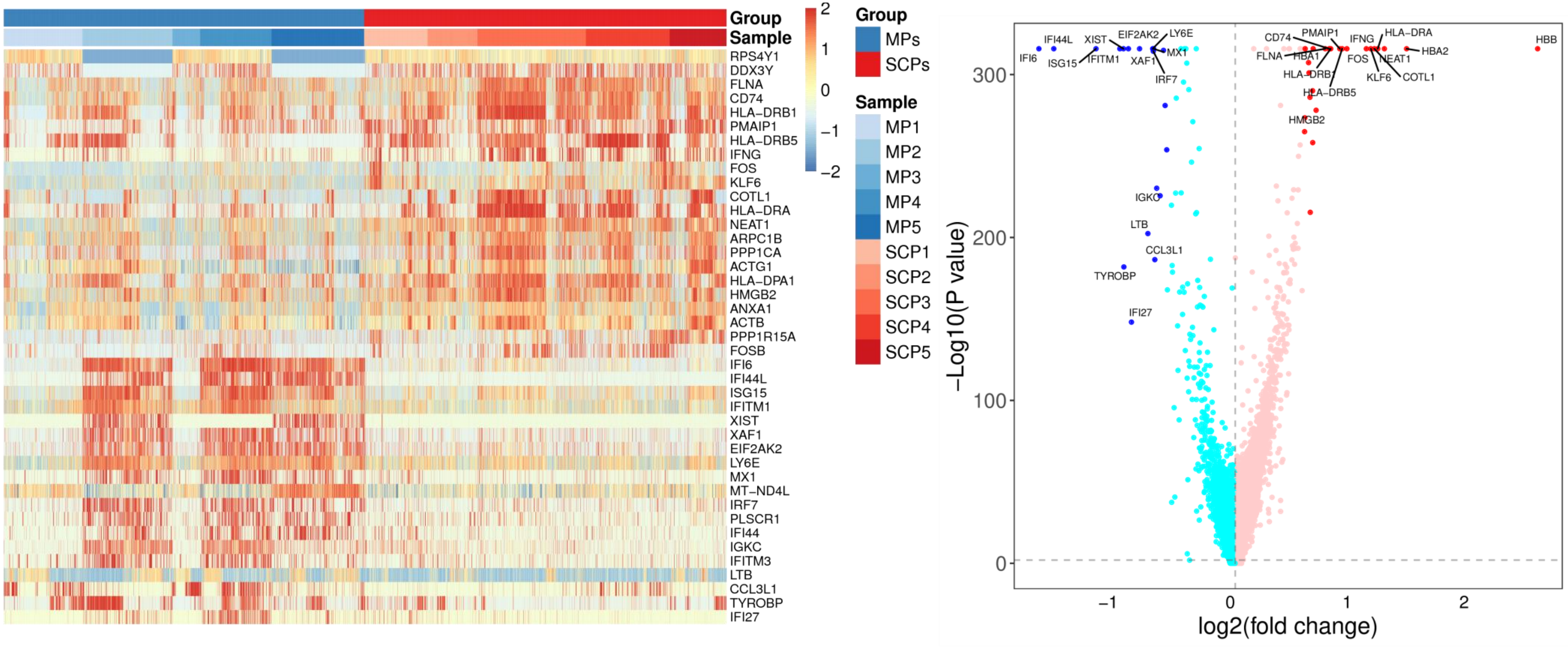

FIGURE S9. (D) The heatmap shows the top DEGs between MPs and SCPs in CD8^{+} T cells. Volcano plot of fold change between MPs and SCPs in CD8^{+} T cells. Findmarker in Seurat v3.1.2 was used to perform differential analysis and the bimod likelihood ratio statistical test was used. Significantly differential expression genes were defined by both p-value (less than and equal to 0.05) and fold change (greater than and equal to 1.5).

CD8^{+} T cells

HCs vs. SCPs

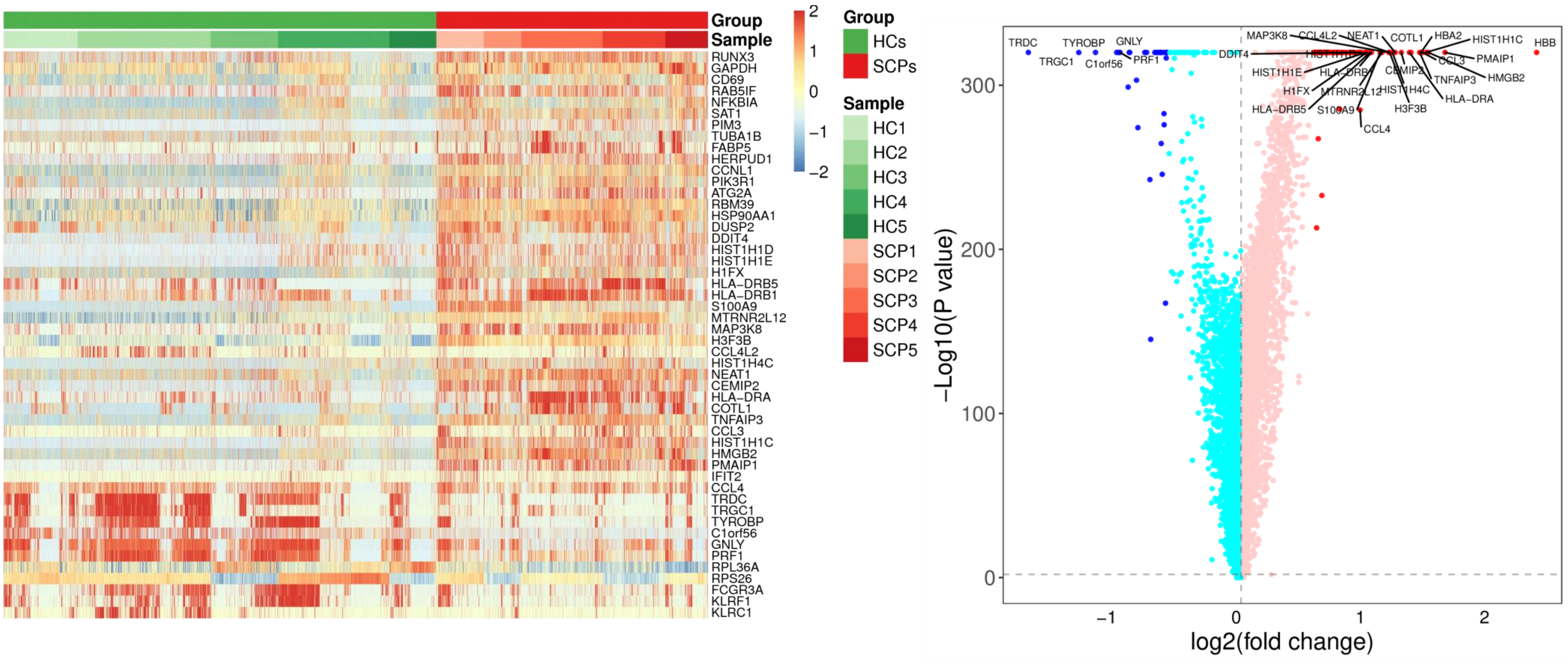

FIGURE S9. (E) The heatmap shows the top DEGs between MPs and SCPs in CD8^{+} T cells. Volcano plot of fold change between MPs and SCPs in CD8^{+} T cells. Findmarker in Seurat v3.1.2 was used to perform differential analysis and the bimod likelihood ratio statistical test was used. Significantly differential expression genes were defined by both p-value (less than and equal to 0.05) and fold change (greater than and equal to 1.5).

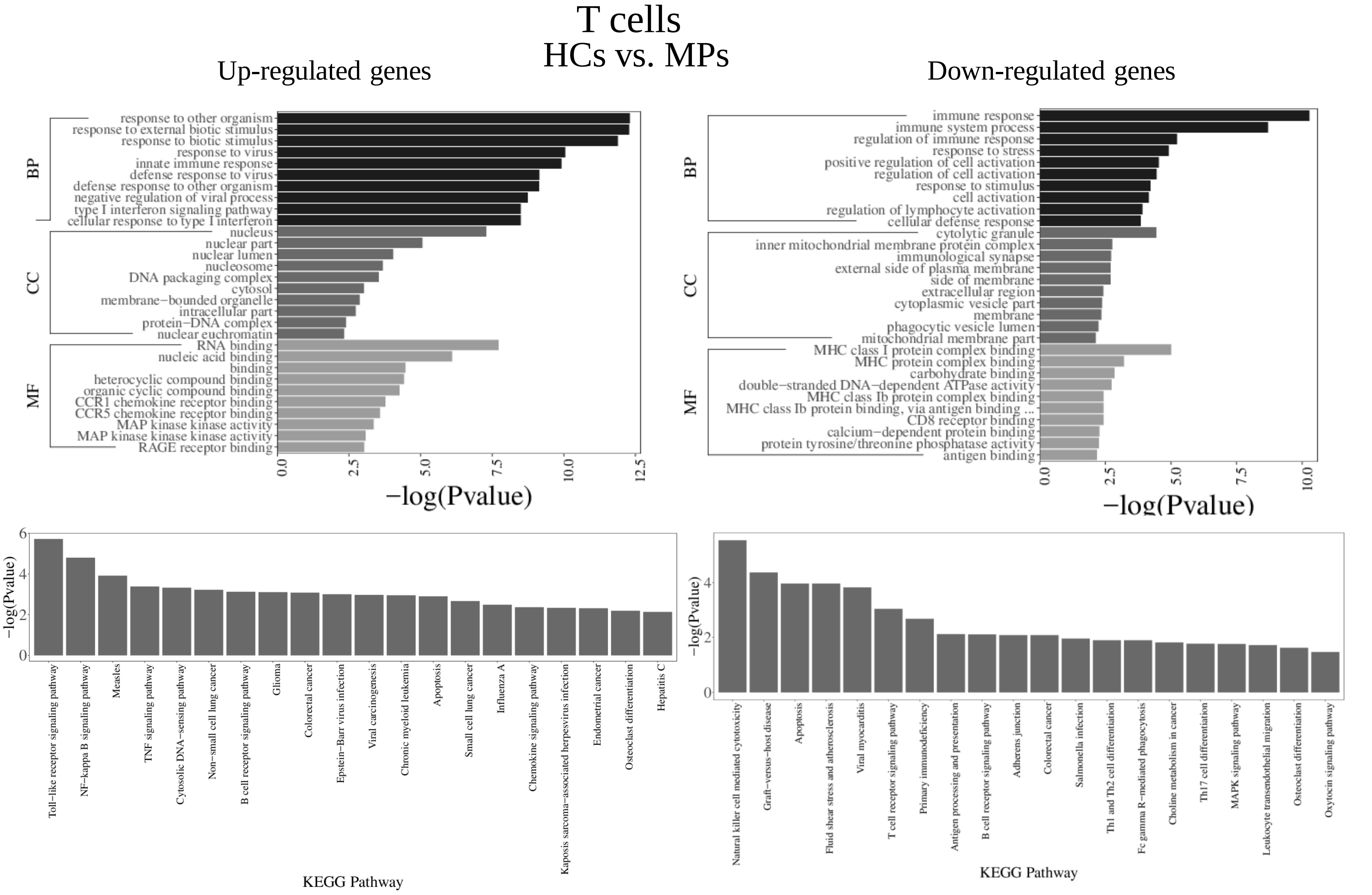

FIGURE S10. (A) The GO and KEGG enrichments of up-regulated DEGs for the T, $CD4^+$ T cells and $CD8^+$ T cells between the group of HCs and MPs by scRNA-seq.

T cells
MPs vs. SCPs

Up-regulated genes

Down-regulated genes

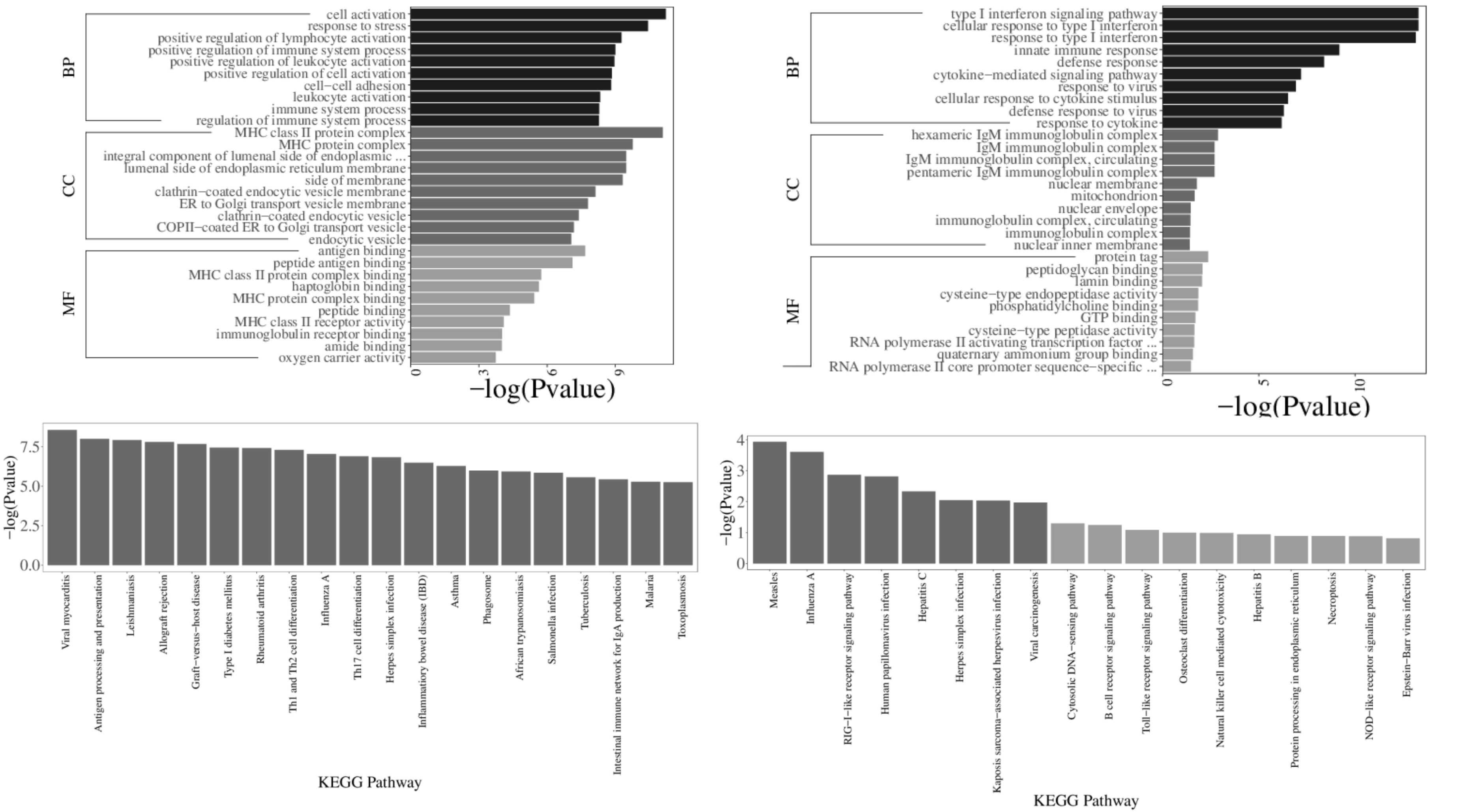

FIGURE S10. (B) The GO and KEGG enrichments of up-regulated DEGs for the T, $CD4^{+}$ T cells and $CD8^{+}$ T cells between the group of MPs and SCPs by scRNA-seq.

T cells
HCs vs. SCPs

Up-regulated genes

Down-regulated genes

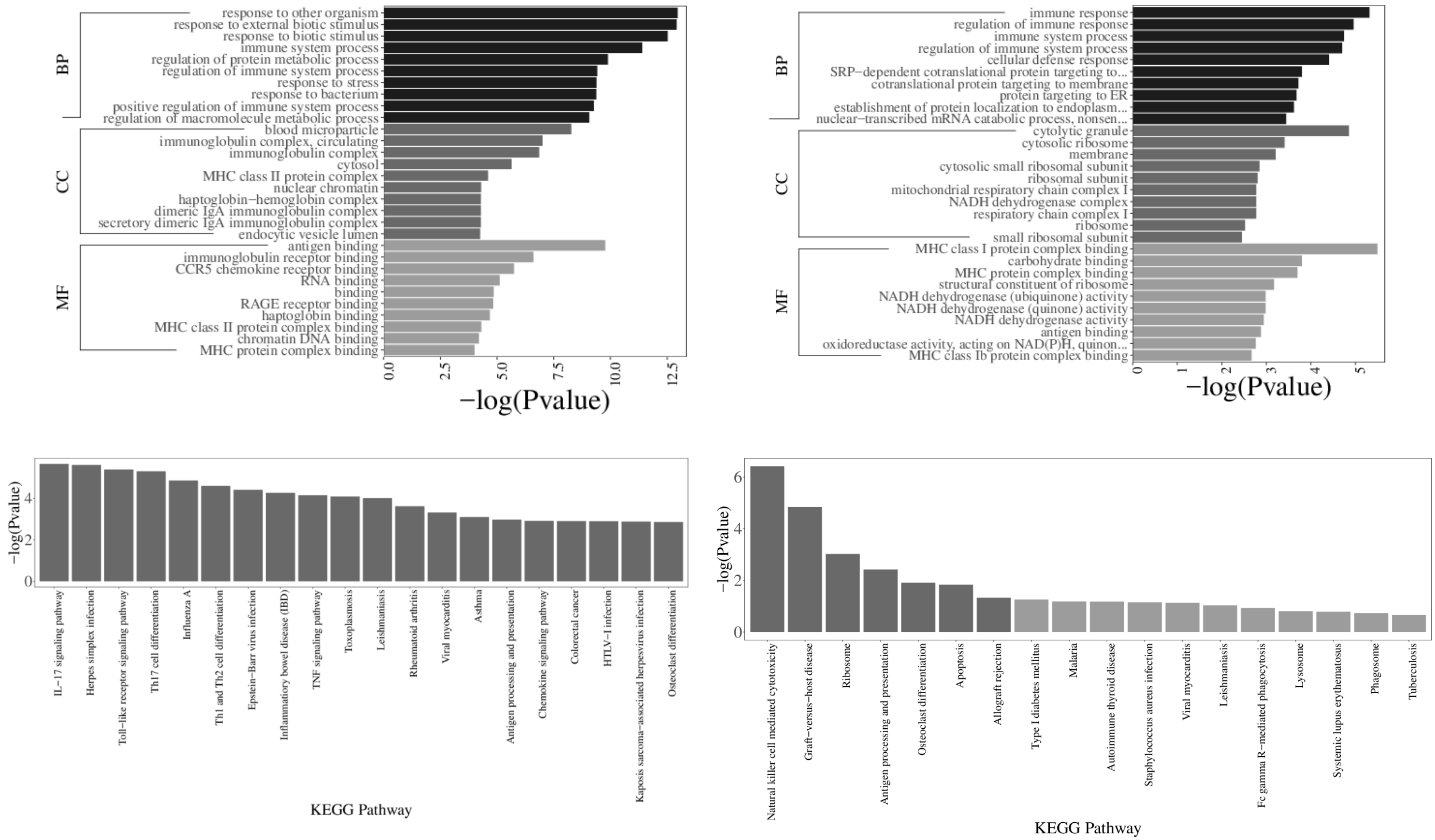

FIGURE S10. (C) The GO and KEGG enrichments of up-regulated DEGs for the T, $CD4^+$ T cells and $CD8^+$ T cells between the group of SCPs and HCs by scRNA-seq.

CD4+ T cells
HCs vs. MPs

Up-regulated genes | Down-regulated genes

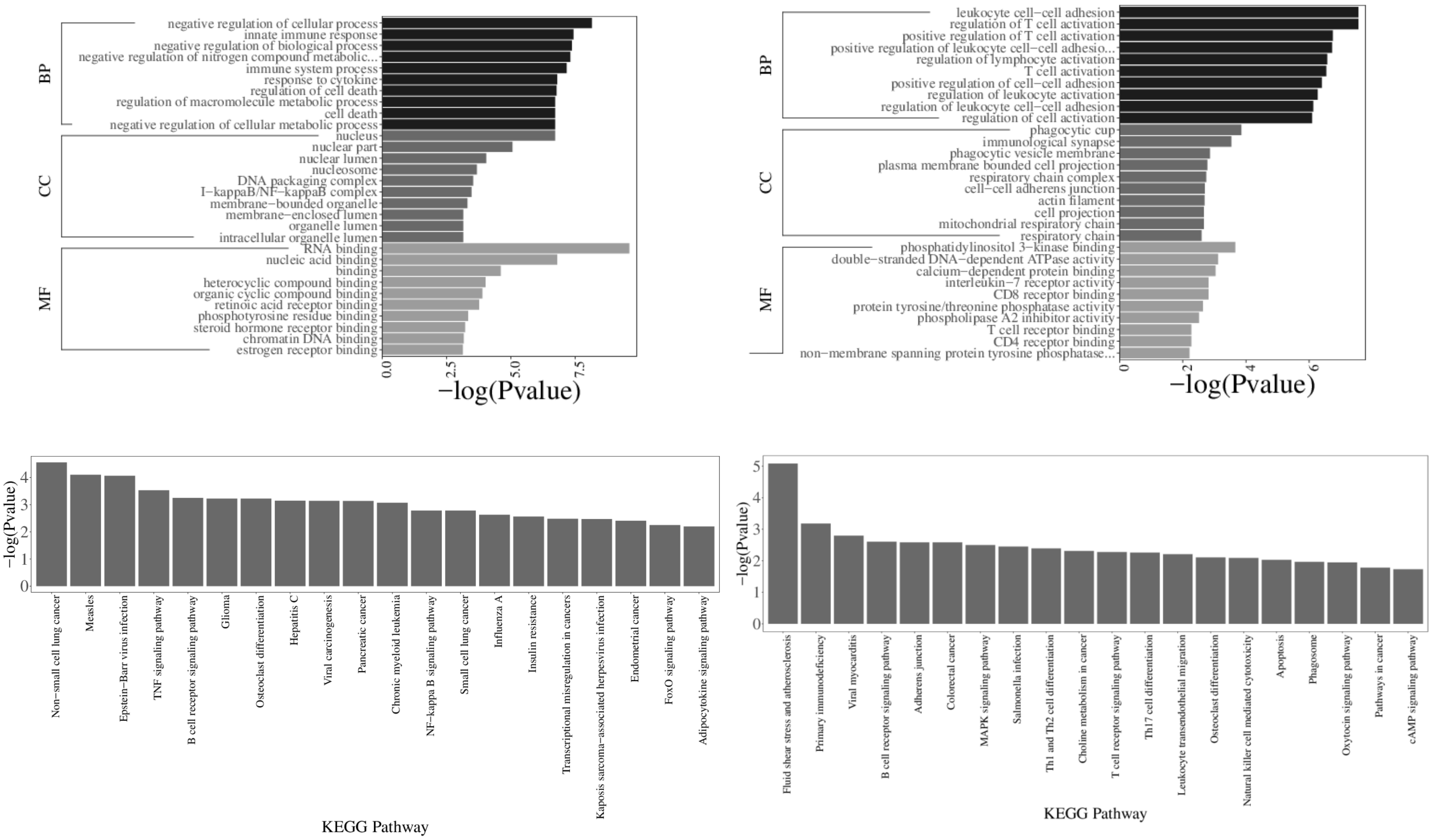

FIGURE S10. (D) The GO and KEGG enrichments of up-regulated DEGs for the T, CD4+ T cells and CD8+ T cells between the group of HCs and MPs by scRNA-seq.

CD4+ T cells
MPs vs. SCPs

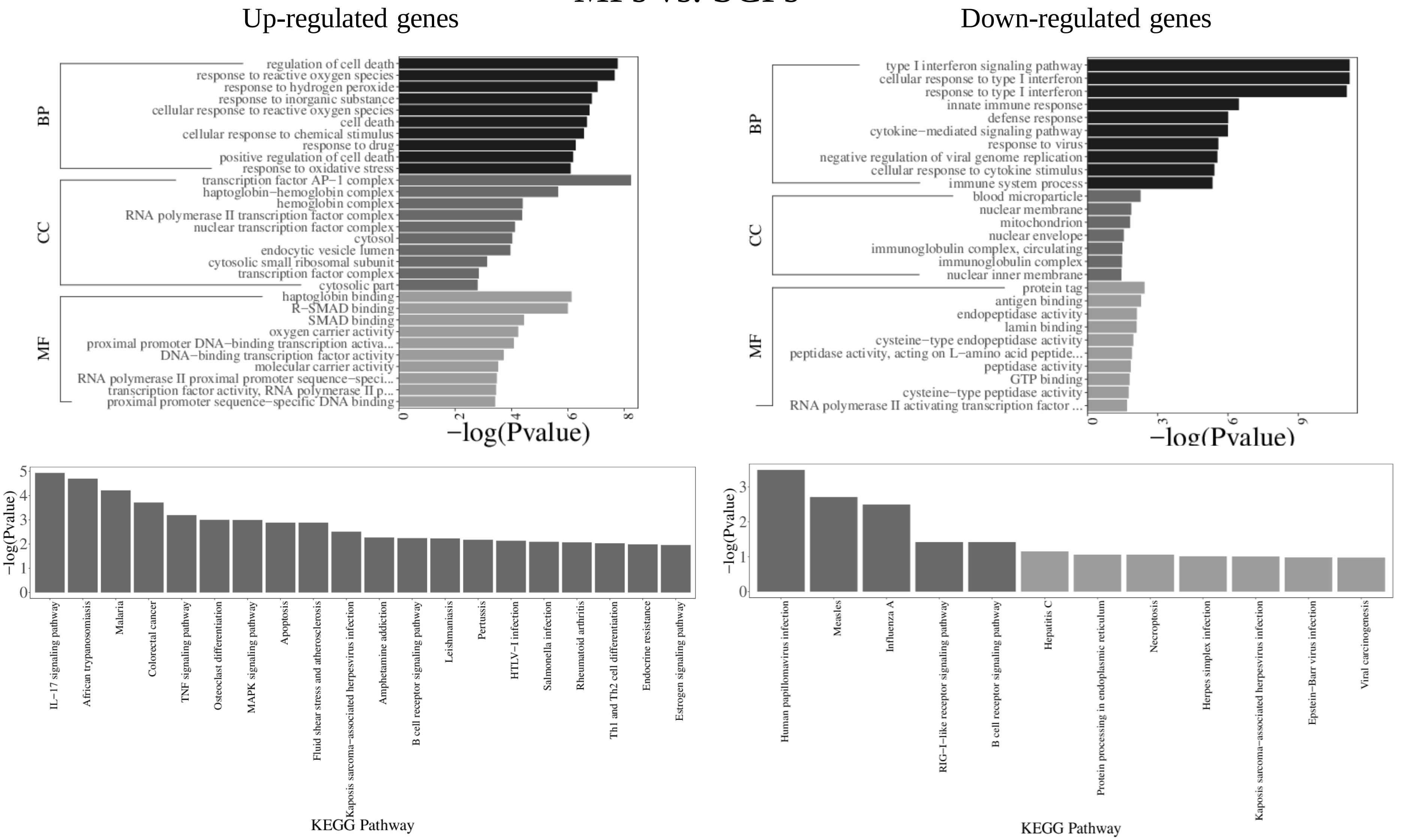

FIGURE S10. (E) The GO and KEGG enrichments of up-regulated DEGs for the T, CD4+ T cells and CD8+ T cells between the group of MPs and SCPs by scRNA-seq.

CD4+ T cells

HCs vs. SCPs

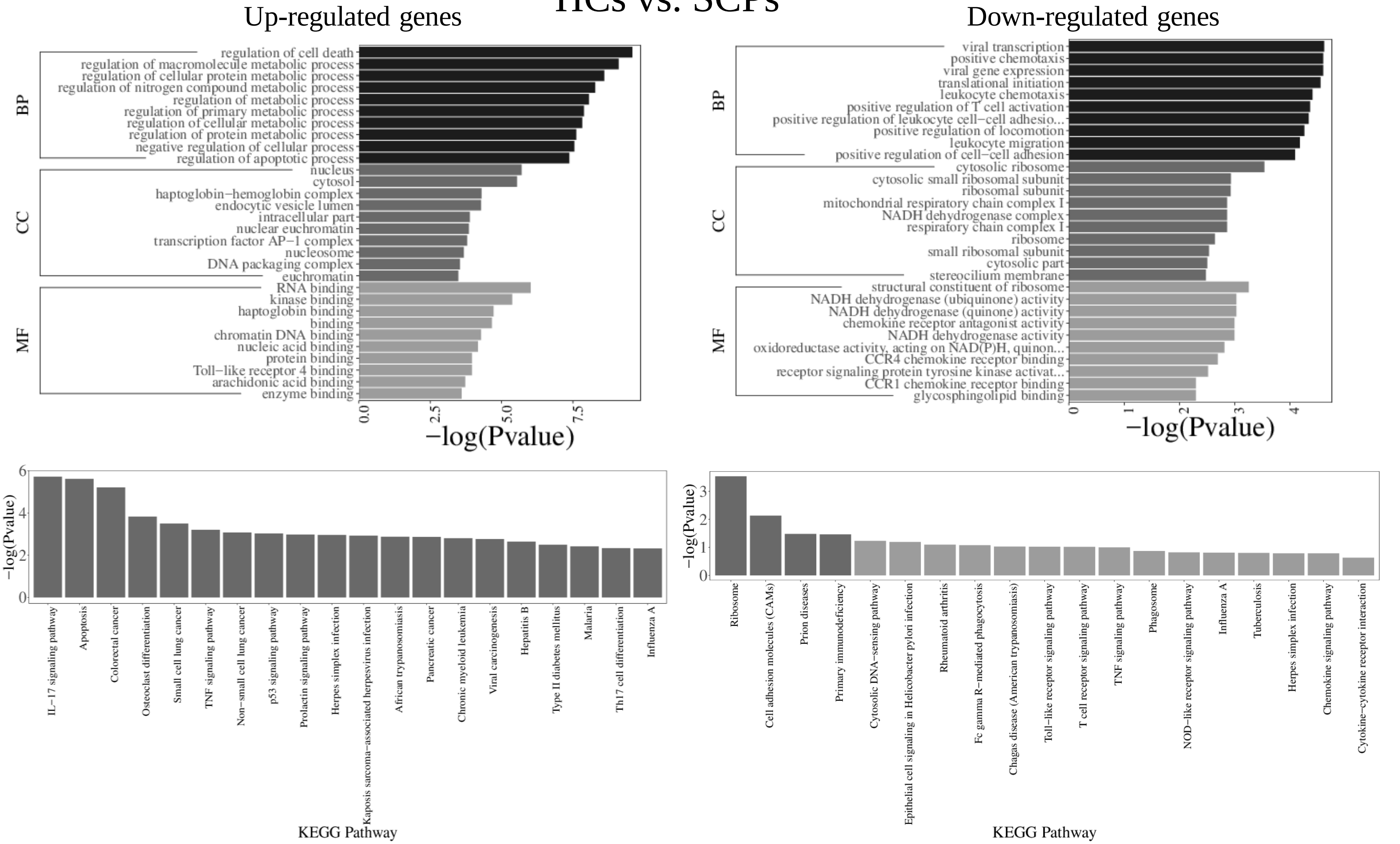

FIGURE S10. (F) The GO and KEGG enrichments of up-regulated DEGs for the T, CD4+ T cells and CD8+ T cells between the group of SCPs and HCs by scRNA-seq.

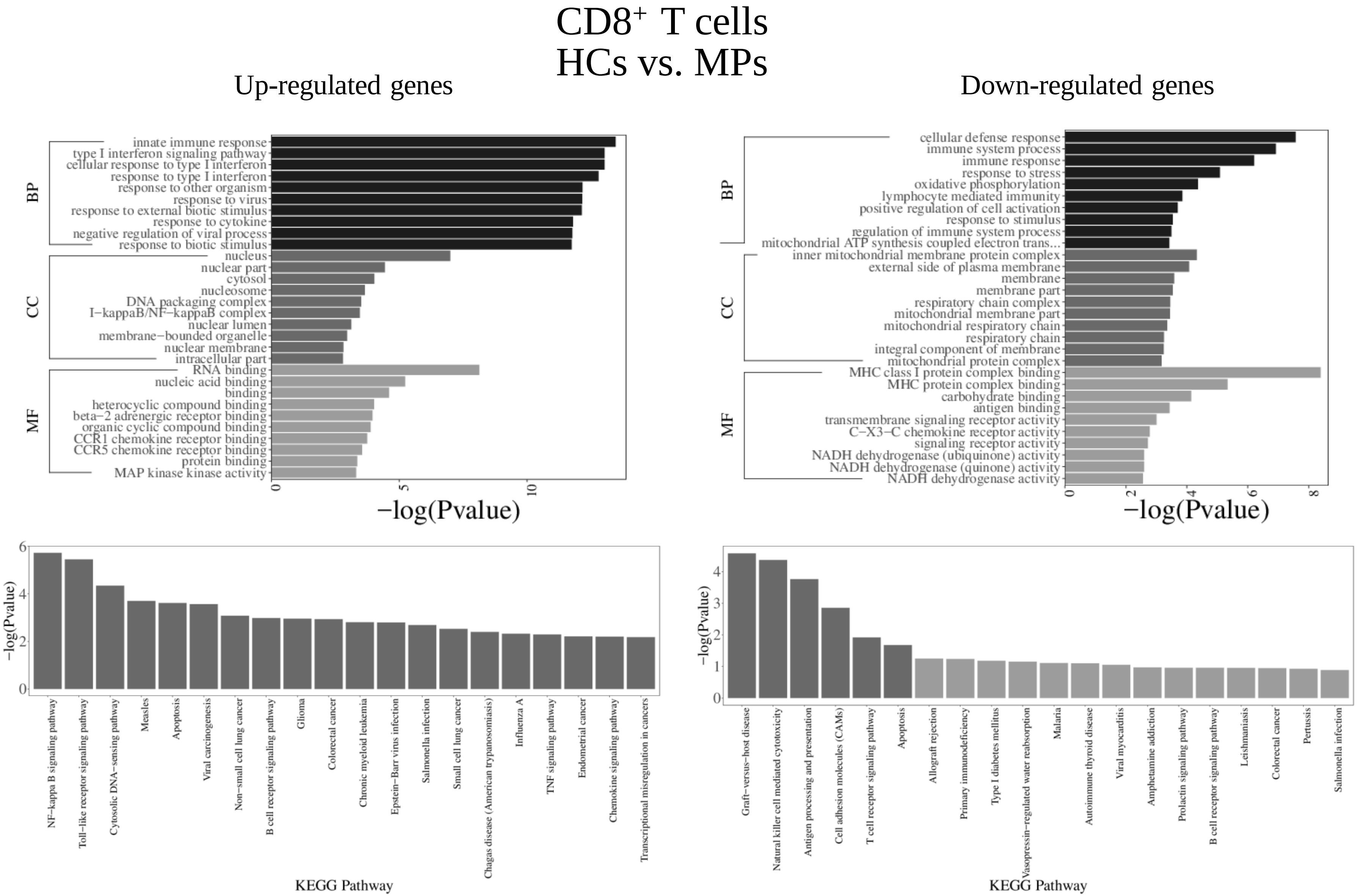

FIGURE S10. (G) The GO and KEGG enrichments of up-regulated DEGs for the T, CD4+ T cells and CD8+ T cells between the group of HCs and MPs by scRNA-seq.

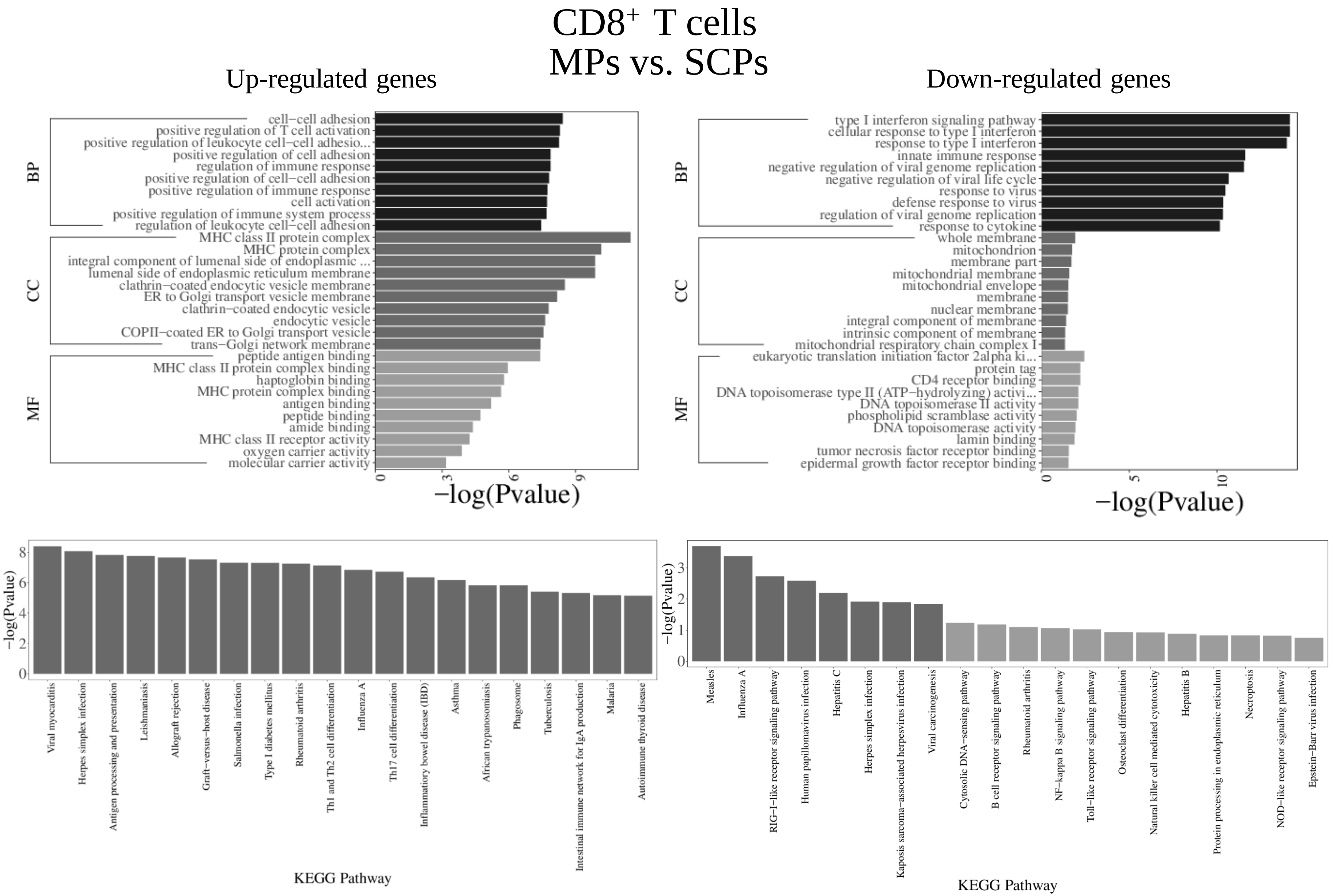

FIGURE S10. (H) The GO and KEGG enrichments of up-regulated DEGs for the T, CD4+ T cells and CD8+ T cells between the group of MPs and SCPs by scRNA-seq.

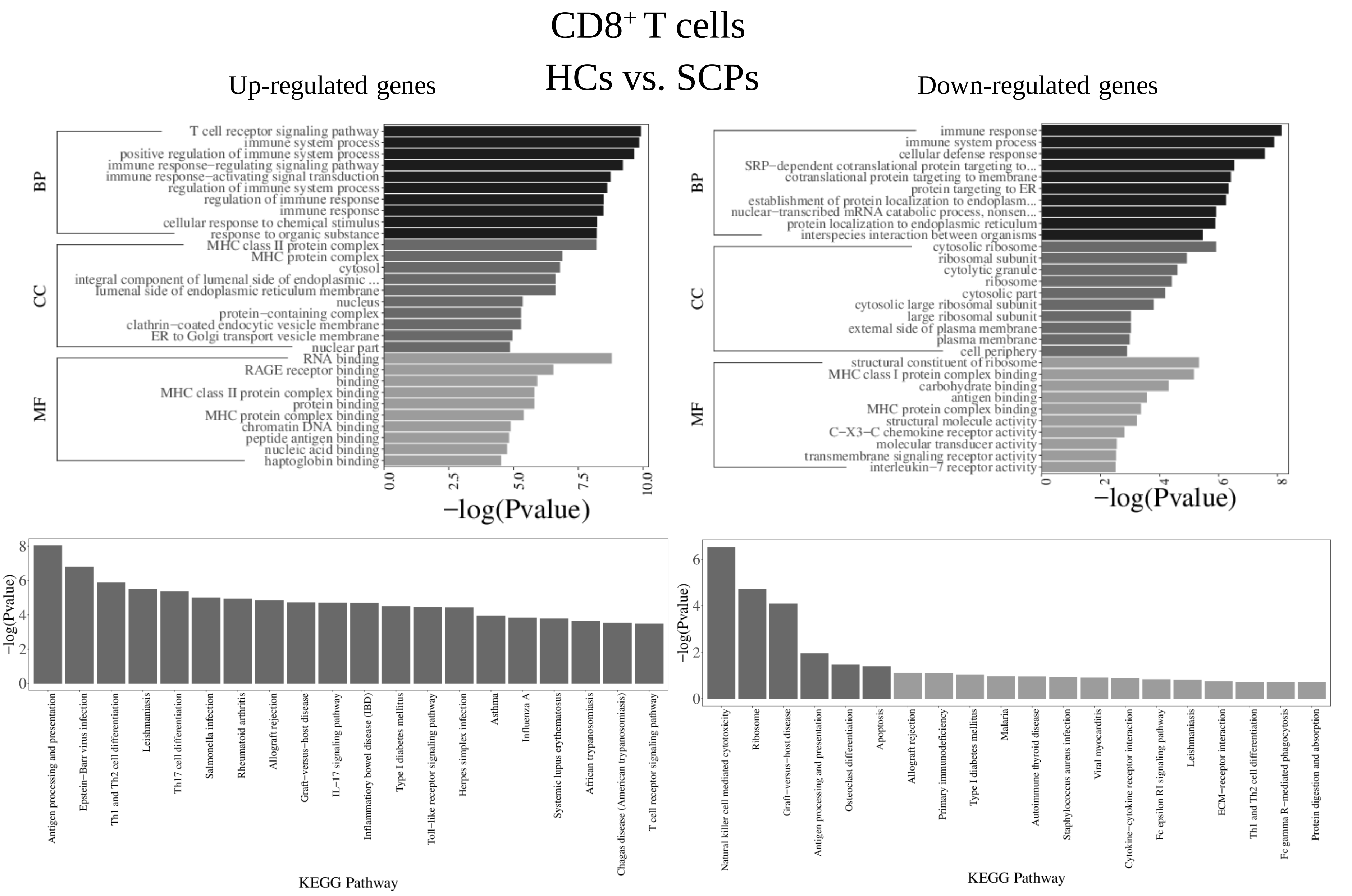

FIGURE S10. (I) The GO and KEGG enrichments of up-regulated DEGs for the T, CD4+ T cells and CD8+ T cells between the group of SCPs and HCs by scRNA-seq.

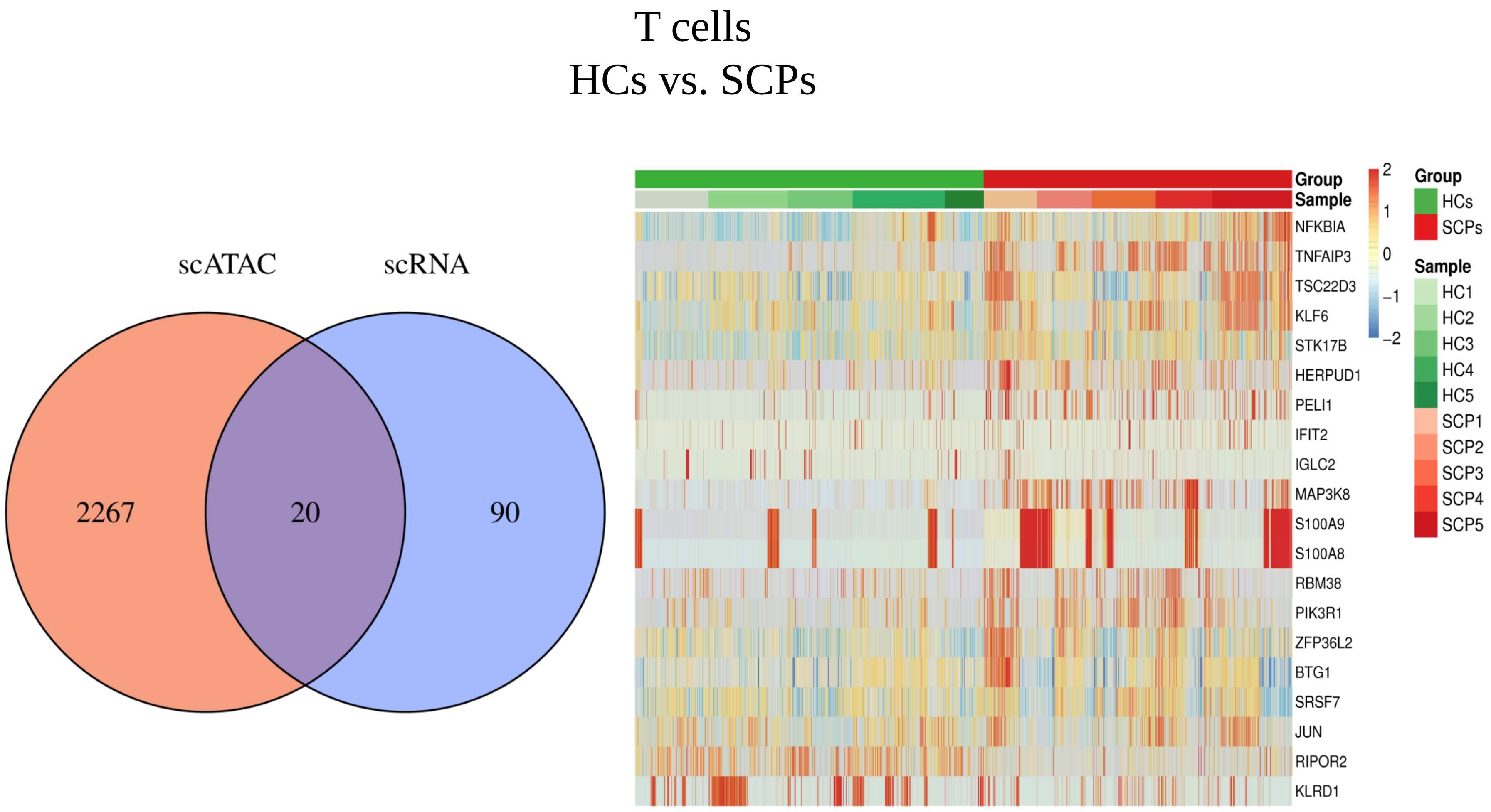

FIGURE S11. (A) Venn diagram and heatmap of all overlapping differential genes for scATAC-seq and scRNA-seq in total T cells from the group of SCPs vs. HCs.

CD4⁺ T cells
HCs vs. SCPs

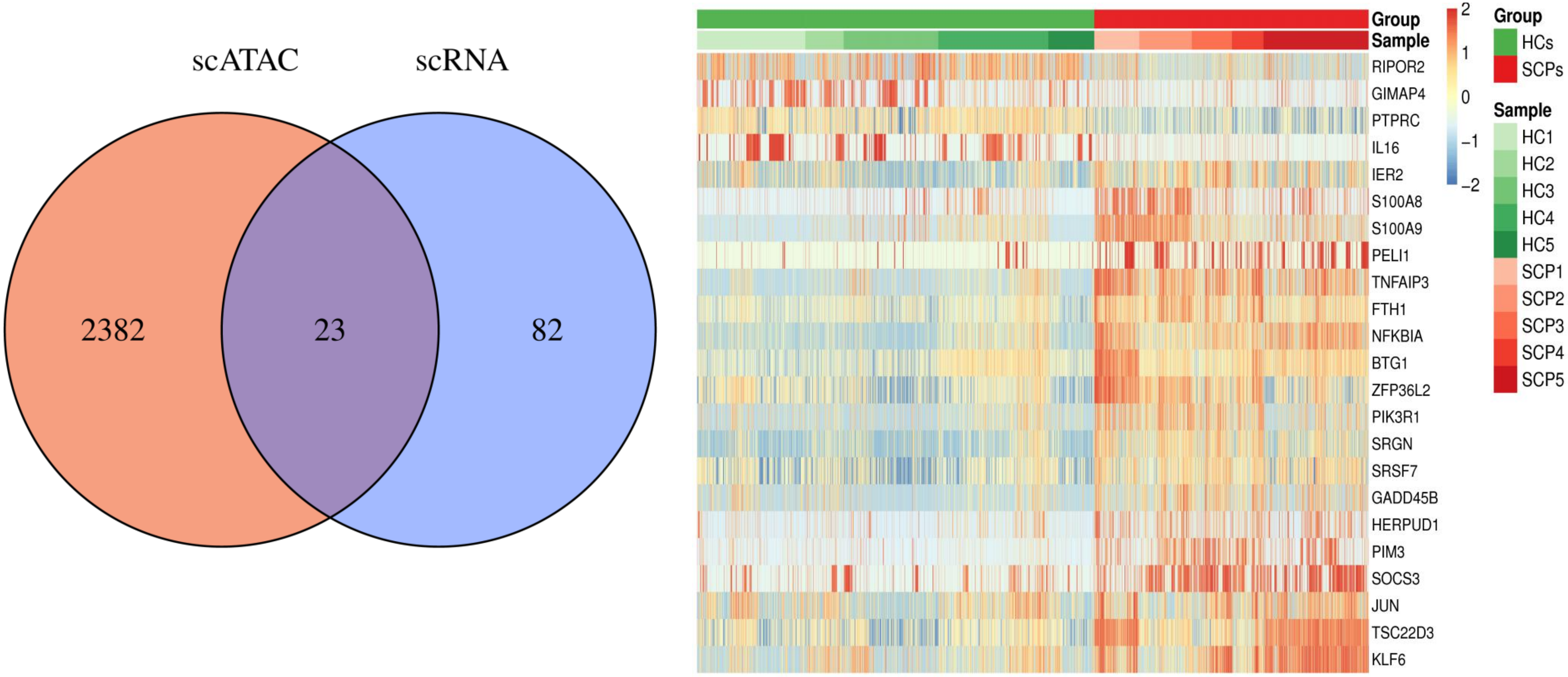

FIGURE S11. (B) Venn diagram and heatmap of all overlapping differential genes for scATAC-seq and scRNA-seq in CD4⁺ T cells from the group of SCPs vs. HCs.

CD8^{+} T cells
HCs vs. MPs

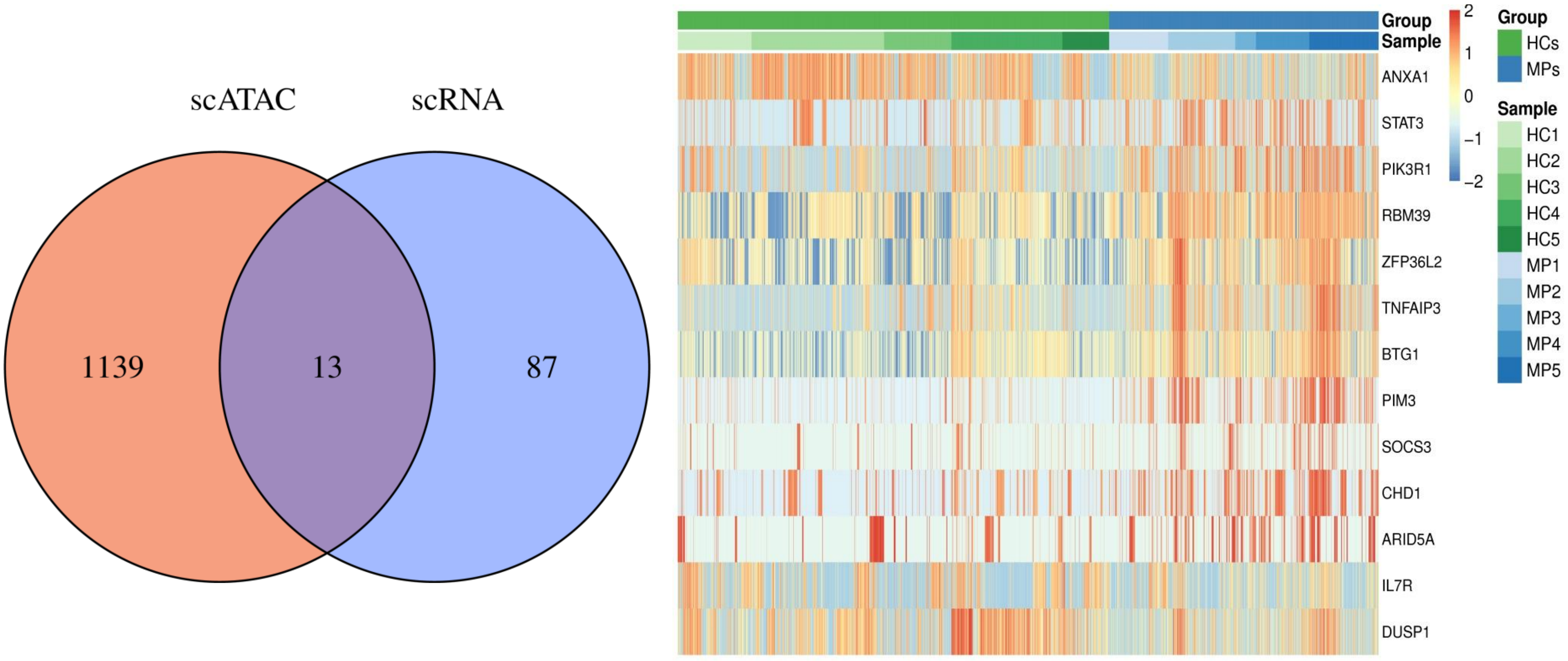

FIGURE S11. (C) Venn diagram and heatmap of all overlapping differential genes for scATAC-seq and scRNA-seq in CD8^{+} T cells from the group of HCs vs. MPs.

CD8+ T cells
MPs vs. SCPs

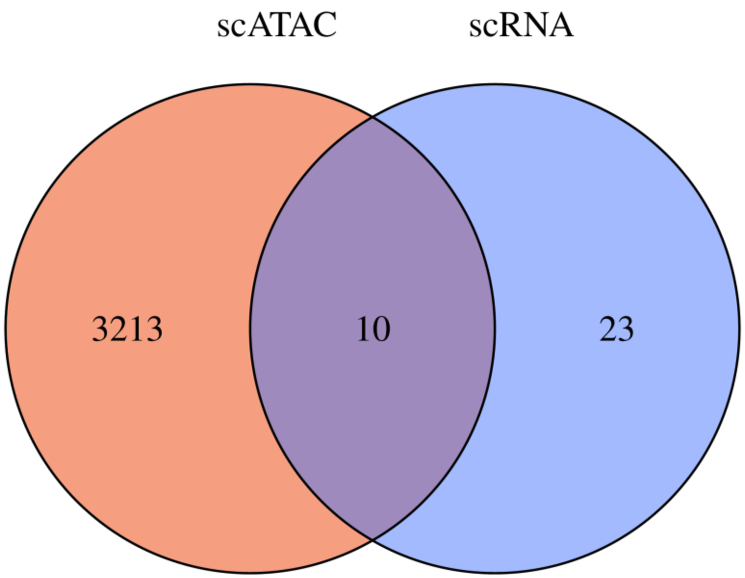

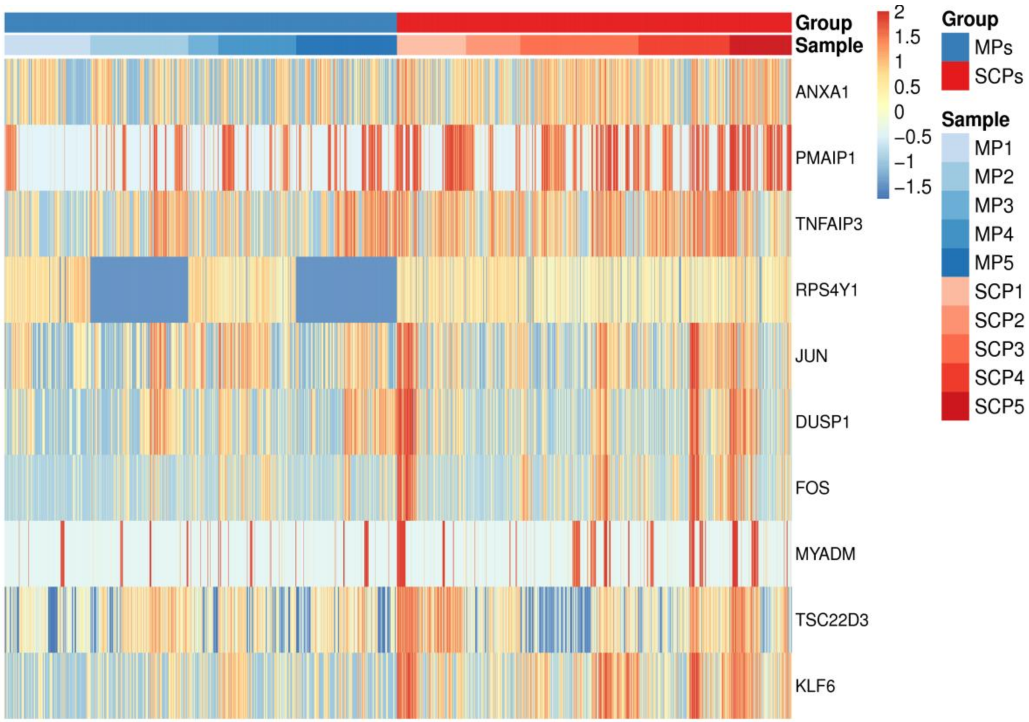

FIGURE S11. (D) Venn diagram and heatmap of all overlapping differential genes for scATAC-seq and scRNA-seq in CD8+ T cells from the group of MPs vs. SCPs.

CD8$^+$ T cells
HCs vs. SCPs

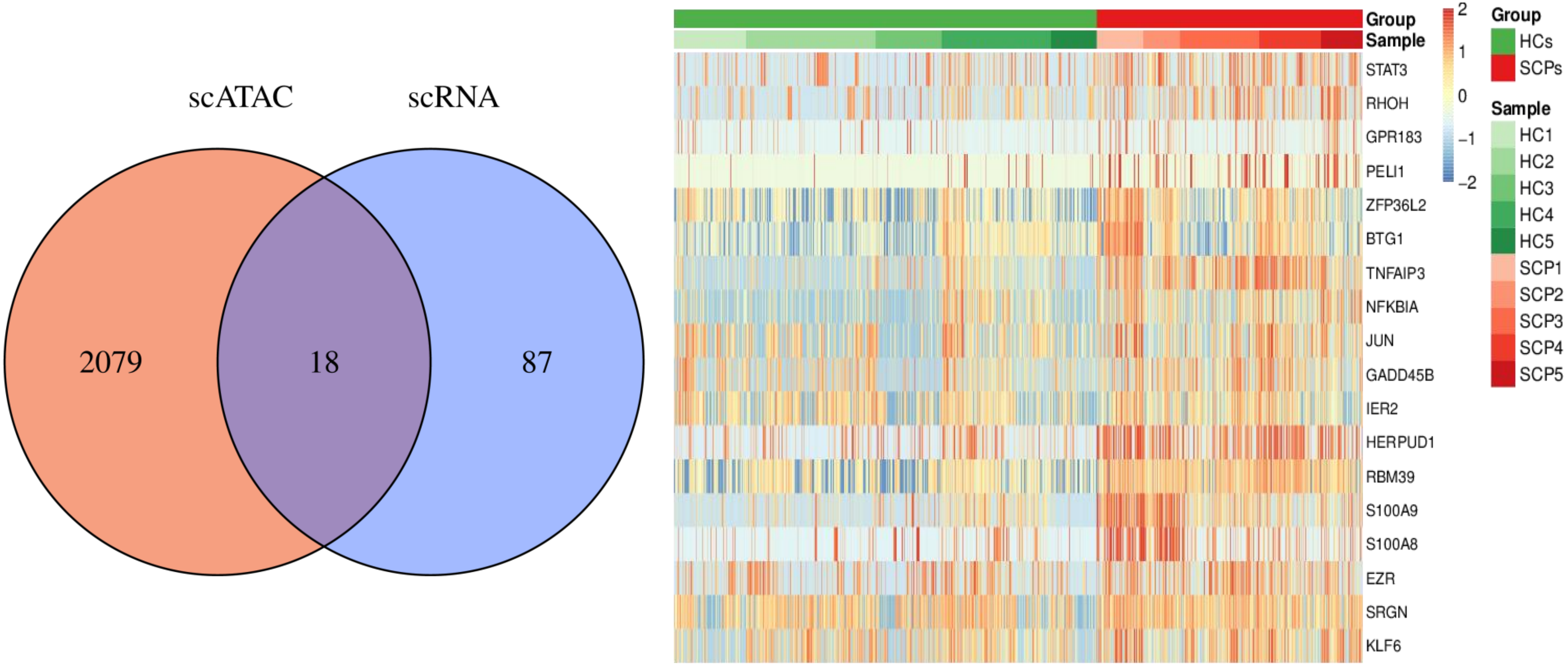

FIGURE S11. (E) Venn diagram and heatmap of all overlapping differential genes for scATAC-seq and scRNA-seq in CD8$^+$ T cells from the group of SCPs vs. HCs.
