# Supplementary material for: Epigenetic Landscapes of Single-Cell Chromatin Accessibility and Transcriptomic Immune Profiles of T Cells in COVID-19 Patients
Source: Front Immunol. 2021 Feb 24;12:625881. doi: 10.3389/fimmu.2021.625881 (PMC7943924; doi:10.3389/fimmu.2021.625881)

10x GENOMICS Cell Ranger ATAC

CT_ZMM_ATAC

Alerts

The analysis detected ⚠ 1 warning.

	Alert	Value	Detail
⚠	Too many detected cells	14,168	Estimated number of cells is expected to be under 10,000. A high value might indicate an overloading of cells, a problem during library preparation, or unexpected behavior in the cell calling algorithm.

For guidance, please consult "Interpreting Cell Ranger ATAC Web Summary Files" or contact 10x Genomics Support (support@10xgenomics.com)

14,168
Estimated number of cells

7,788
Median fragments per cell

72.8%
Fraction of fragments overlapping any targeted region

53.9%
Fraction of transposition events in peaks in cell barcodes

Sample

Sample ID	CT_ZMM_ATAC
Sample description	
FASTQ path	…200508/ATAC/CT_ZMM_ATAC
Pipeline version	1.2.0
Reference path	…abase/ATAC/Homo_sapiens
Organism	Homo_sapiens
Assembly	custom
Annotation	custom

Sequencing

Total number of read pairs	333,085,962
Fraction of read pairs with a valid barcode	97.6%
Q30 bases in Read 1	92.1%
Q30 bases in Read 2	92.2%
Q30 bases in Barcode	90.9%
Q30 bases in Sample Index	92.5%

Cells

Estimated number of cells	14,168
Lower threshold on the number of fragments overlapping peaks per barcode to annotate barcode as cell	1,445.00
Median fragments per cell	7,788
Median fragments per non-cell barcode	2

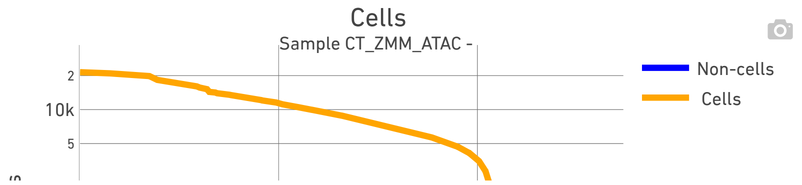

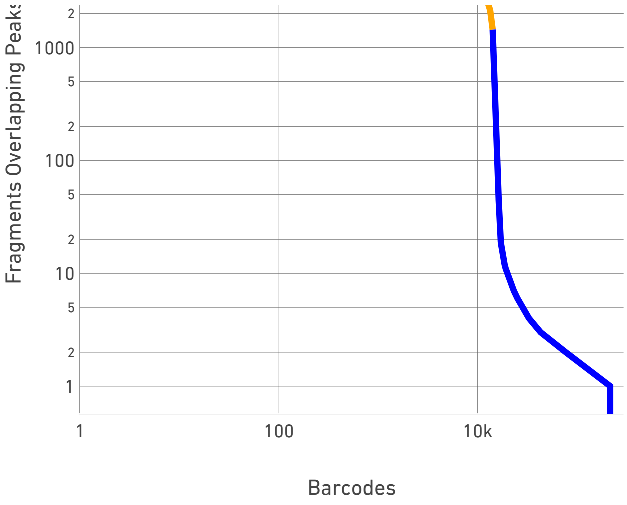

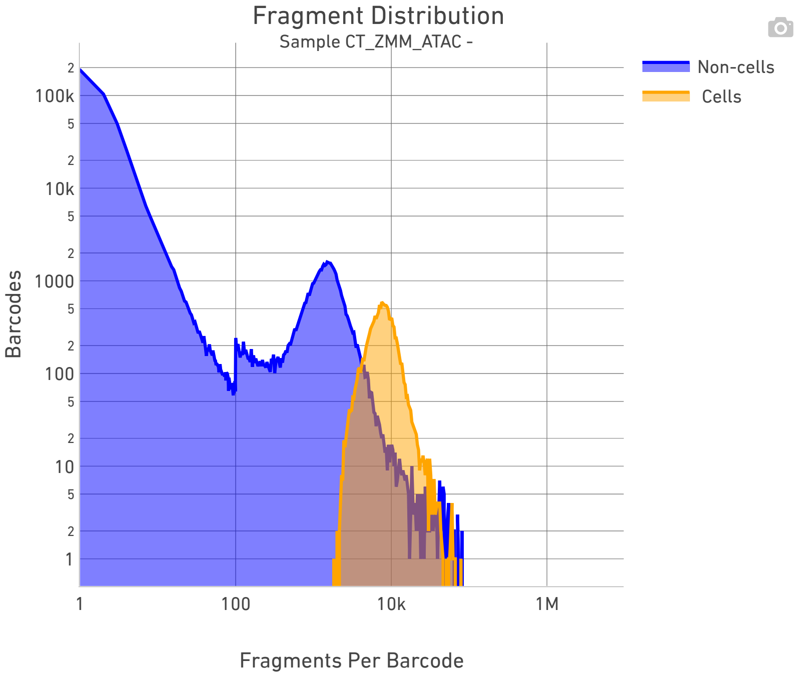

Cell Clustering

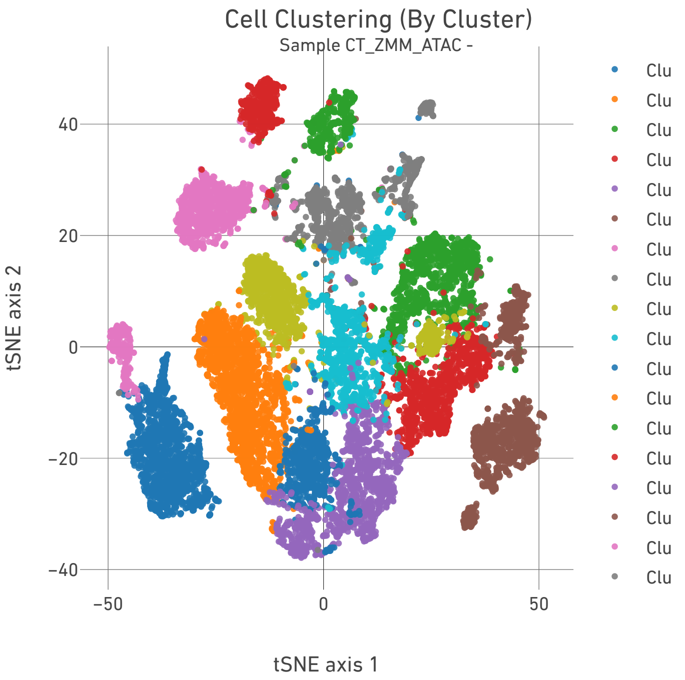

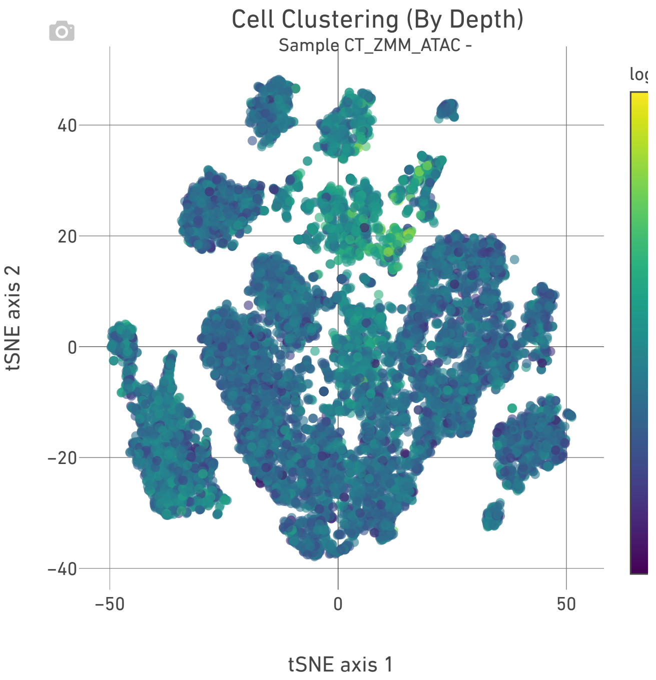

Insert Sizes

Fragments in nucleosome-free regions	42.9%
Fragments flanking a single nucleosome	47.3%

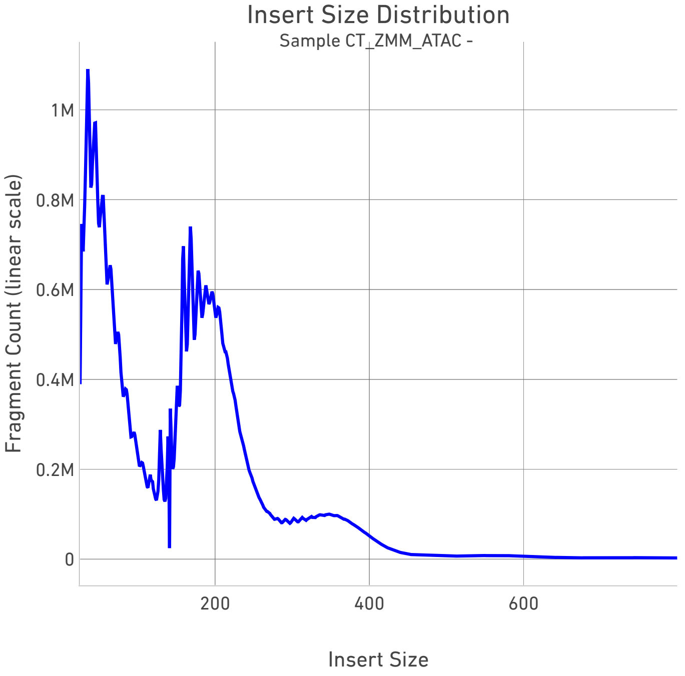

Targeting

Enrichment score of transcription start sites	6.80
Fraction of fragments overlapping TSS	42.0%
Fraction of fragments overlapping called peaks	55.6%
Fraction of transposition events in peaks in cell barcodes	53.9%
Fraction of fragments overlapping any targeted region	72.8%

Fraction of total read pairs mapped confidently to genome (>30 mapq)	84.2%
Fraction of total read pairs that are unmapped and in cell barcodes	0.6%
Fraction of total read pairs in mitochondria and in cell barcodes	0.7%

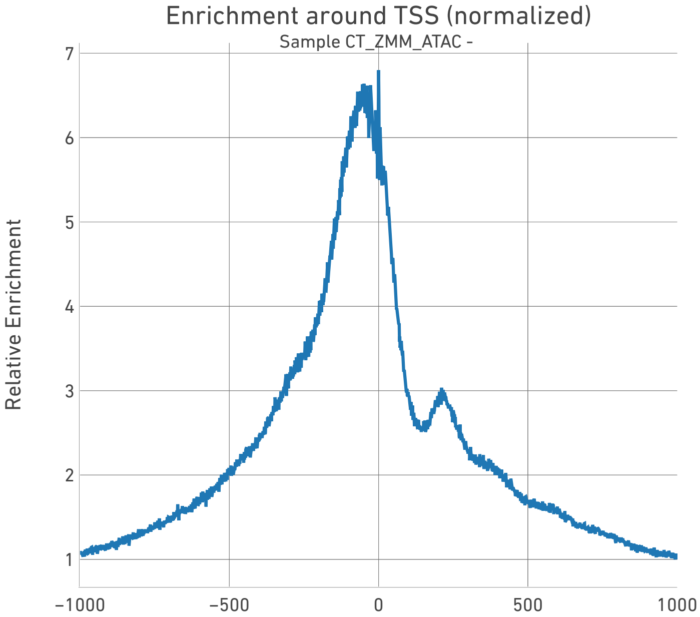

Relative Position (bp from TSS)

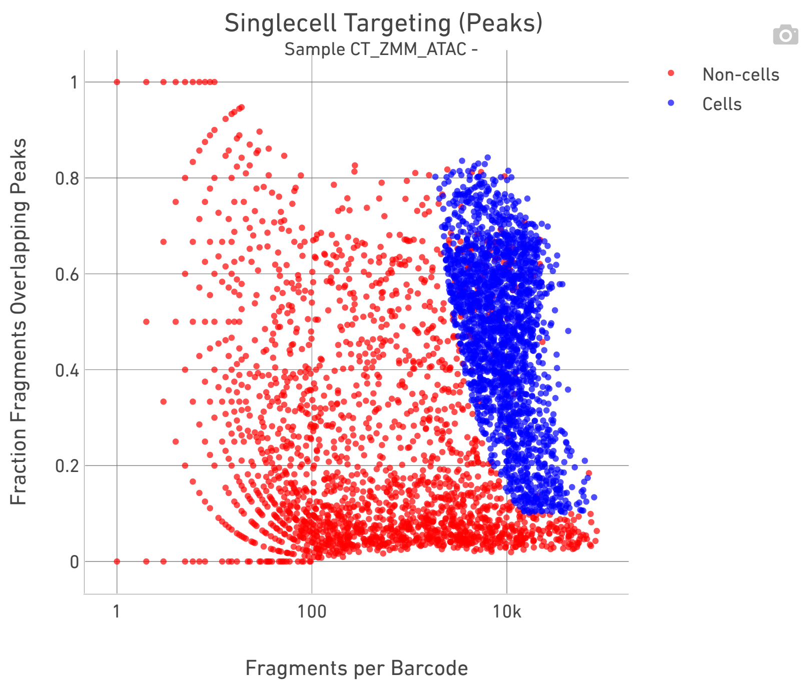

Library Complexity

Percent duplicates	12.5%
Sequencing saturation	27.7%
Estimated bulk library complexity	767,280,701

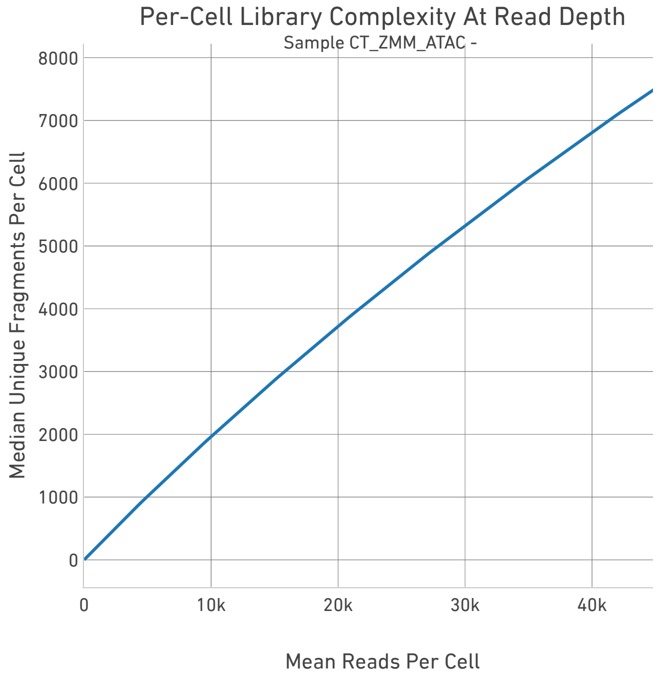

Supplement: Supplementary file 16 [file Data_Sheet_2.PDF]
