# Supplementary material for: Epigenetic Landscapes of Single-Cell Chromatin Accessibility and Transcriptomic Immune Profiles of T Cells in COVID-19 Patients
Source: Front Immunol. 2021 Feb 24;12:625881. doi: 10.3389/fimmu.2021.625881 (PMC7943924; doi:10.3389/fimmu.2021.625881)

10x GENOMICS Cell Ranger ATAC

CT_MGZ_ATAC

For guidance, please consult "Interpreting Cell Ranger ATAC Web Summary Files" or contact 10x Genomics Support (support@10xgenomics.com)

9,701
Estimated number of cells

7,610
Median fragments per cell

72.2%
Fraction of fragments overlapping any targeted region

54.0%
Fraction of transposition events in peaks in cell barcodes

Sample

Sample ID	CT_MGZ_ATAC
Sample description	
FASTQ path	…200508/ATAC/CT_MGZ_ATAC
Pipeline version	1.2.0
Reference path	…abase/ATAC/Homo_sapiens
Organism	Homo_sapiens
Assembly	custom
Annotation	custom

Sequencing

Total number of read pairs	334,236,700
Fraction of read pairs with a valid barcode	97.6%
Q30 bases in Read 1	92.0%
Q30 bases in Read 2	92.2%
Q30 bases in Barcode	90.9%
Q30 bases in Sample Index	93.4%

Cells

Estimated number of cells	9,701
Lower threshold on the number of fragments overlapping peaks per barcode to annotate barcode as cell	121.00
Median fragments per cell	7,610
Median fragments per non-cell barcode	1

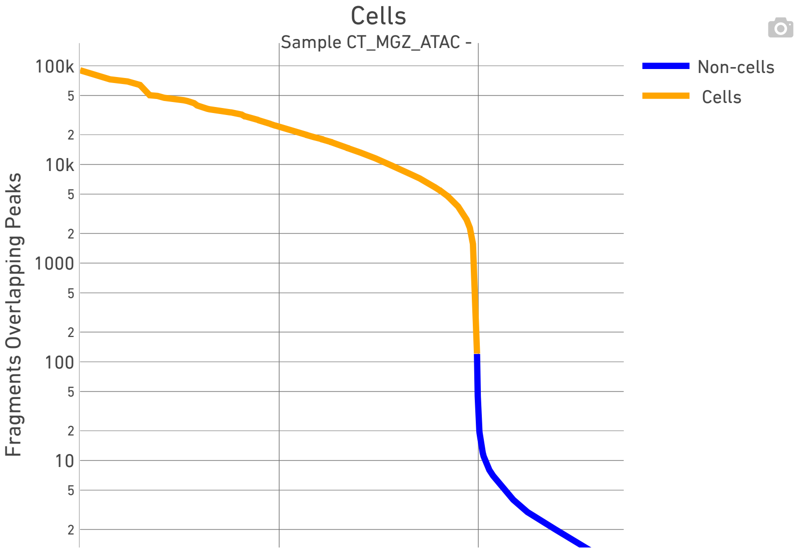

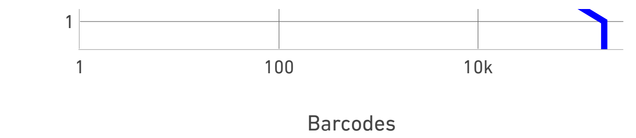

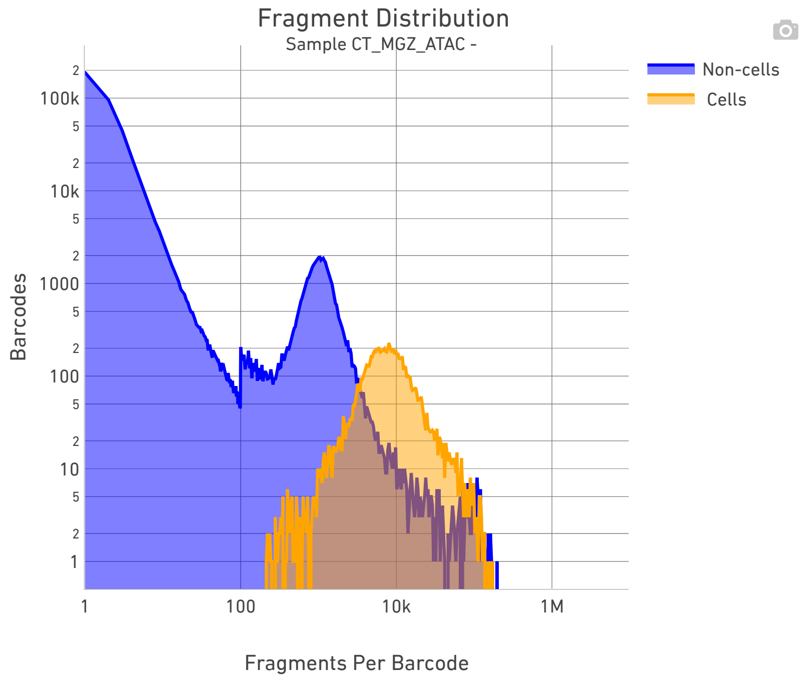

Cell Clustering

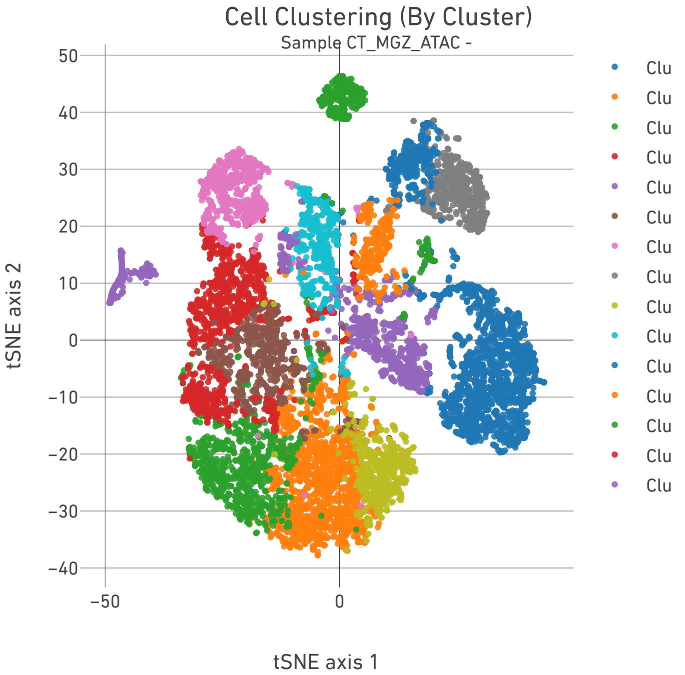

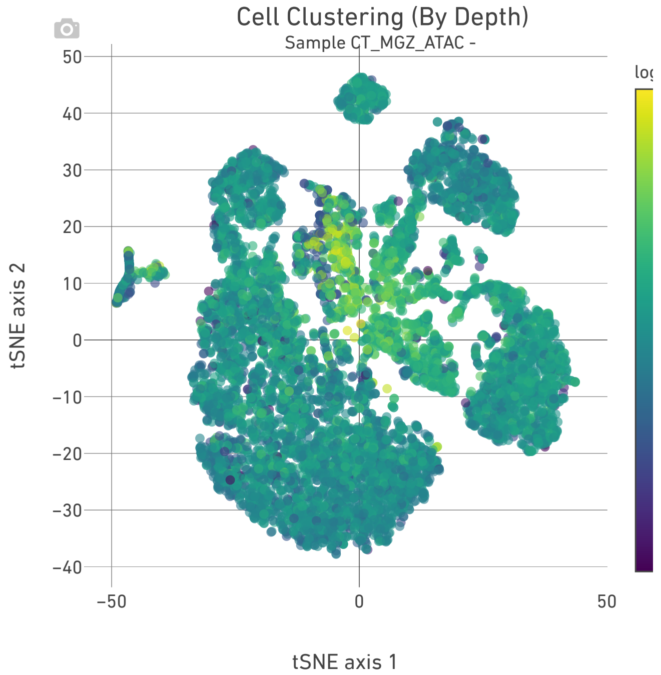

Insert Sizes

Fragments in nucleosome-free regions	57.6%
Fragments flanking a single nucleosome	36.6%

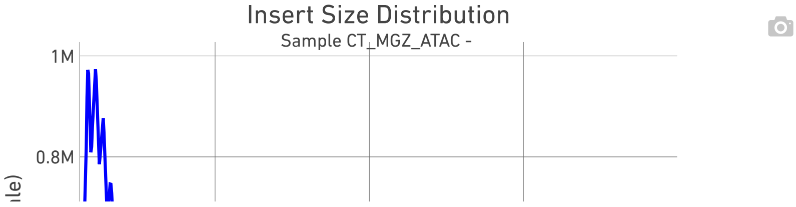

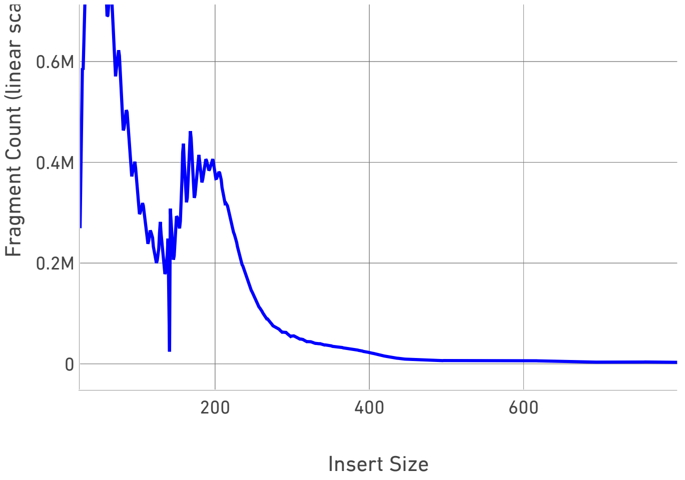

Targeting

Enrichment score of transcription start sites	7.97
Fraction of fragments overlapping TSS	41.3%
Fraction of fragments overlapping called peaks	55.4%
Fraction of transposition events in peaks in cell barcodes	54.0%
Fraction of fragments overlapping any targeted region	72.2%

Fraction of total read pairs mapped confidently to genome (>30 mapq)	80.2%
Fraction of total read pairs that are unmapped and in cell barcodes	1.2%
Fraction of total read pairs in mitochondria and in cell barcodes	0.7%

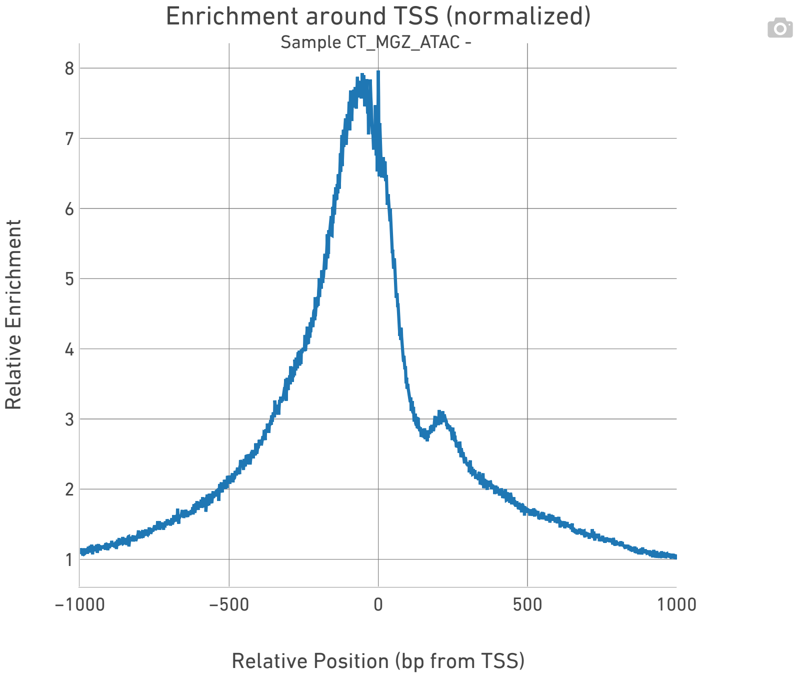

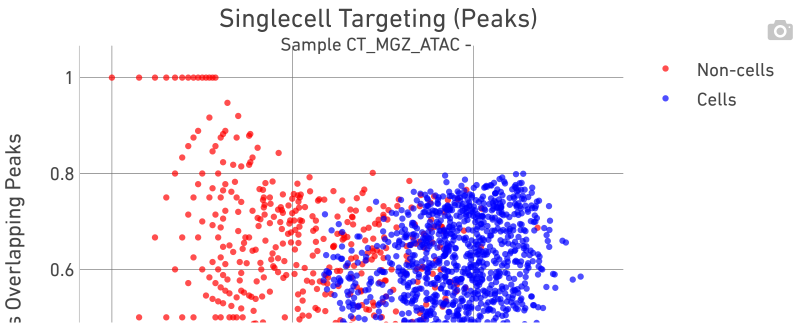

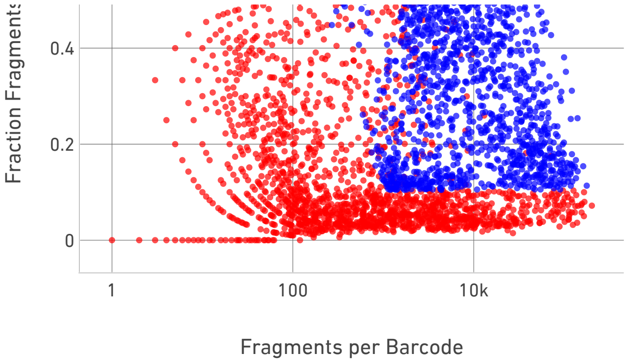

Library Complexity

Percent duplicates	15.2%
Sequencing saturation	37.9%
Estimated bulk library complexity	486,933,264

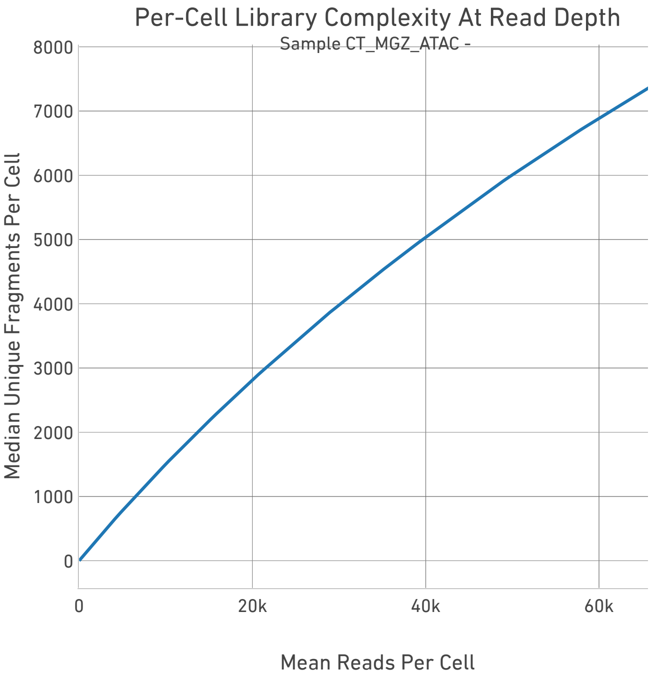

Supplement: Supplementary file 17 [file Data_Sheet_3.PDF]
