# Supplementary material for: Epigenetic Landscapes of Single-Cell Chromatin Accessibility and Transcriptomic Immune Profiles of T Cells in COVID-19 Patients
Source: Front Immunol. 2021 Feb 24;12:625881. doi: 10.3389/fimmu.2021.625881 (PMC7943924; doi:10.3389/fimmu.2021.625881)

10x GENOMICS Cell Ranger ATAC

LWY_ATAC

Alerts

The analysis detected ⚠ 1 warning.

	Alert	Value	Detail
⚠	Too many detected cells	14,292	Estimated number of cells is expected to be under 10,000. A high value might indicate an overloading of cells, a problem during library preparation, or unexpected behavior in the cell calling algorithm.

For guidance, please consult "Interpreting Cell Ranger ATAC Web Summary Files" or contact 10x Genomics Support (support@10xgenomics.com)

14,292
Estimated number of cells

6,784
Median fragments per cell

66.2%
Fraction of fragments overlapping any targeted region

44.0%
Fraction of transposition events in peaks in cell barcodes

Sample

Sample ID	LWY_ATAC
Sample description	
FASTQ path	…_20200508/ATAC/LWY_ATAC
Pipeline version	1.2.0
Reference path	…abase/ATAC/Homo_sapiens
Organism	Homo_sapiens
Assembly	custom
Annotation	custom

Sequencing

Total number of read pairs	372,389,741
Fraction of read pairs with a valid barcode	98.5%
Q30 bases in Read 1	94.1%
Q30 bases in Read 2	94.2%
Q30 bases in Barcode	93.1%
Q30 bases in Sample Index	93.3%

Cells

Estimated number of cells	14,292
Lower threshold on the number of fragments overlapping peaks per barcode to annotate barcode as cell	181.00
Median fragments per cell	6,784
Median fragments per non-cell barcode	2

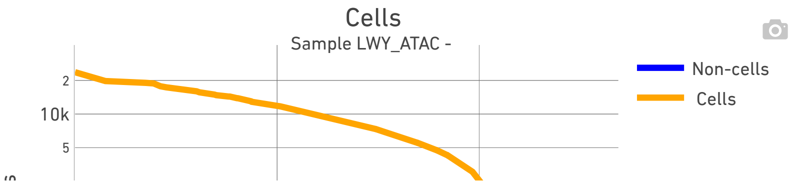

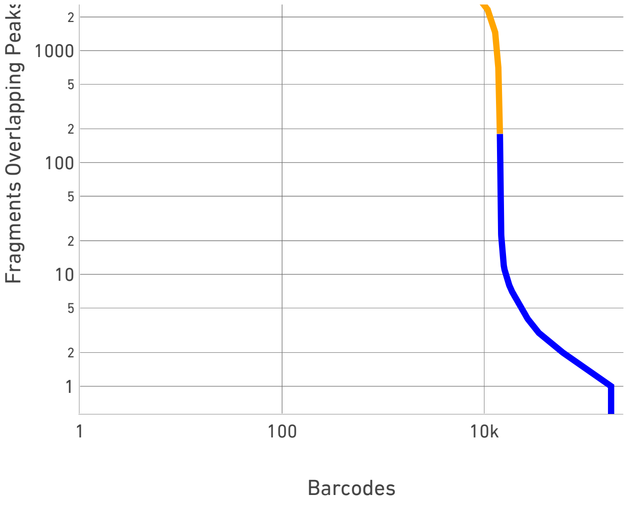

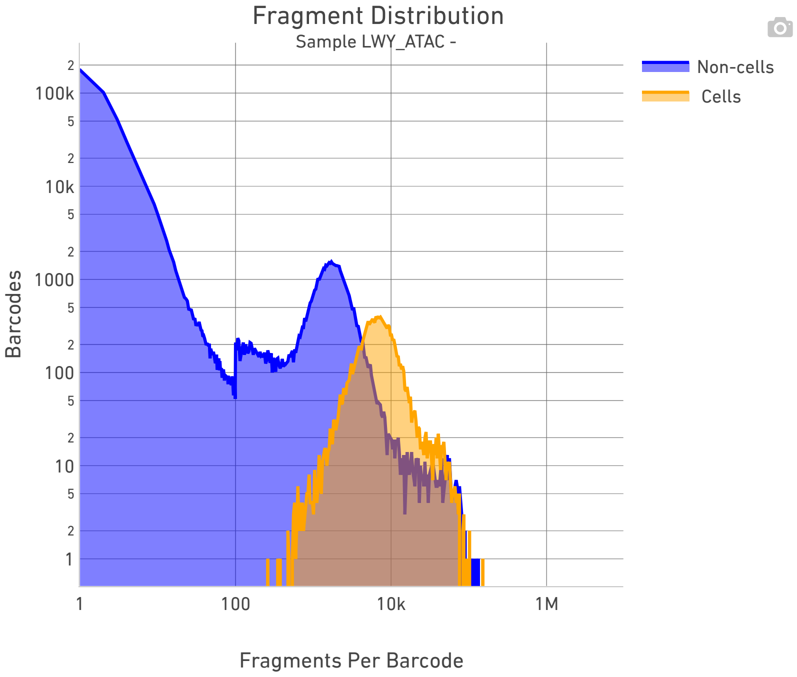

Cell Clustering

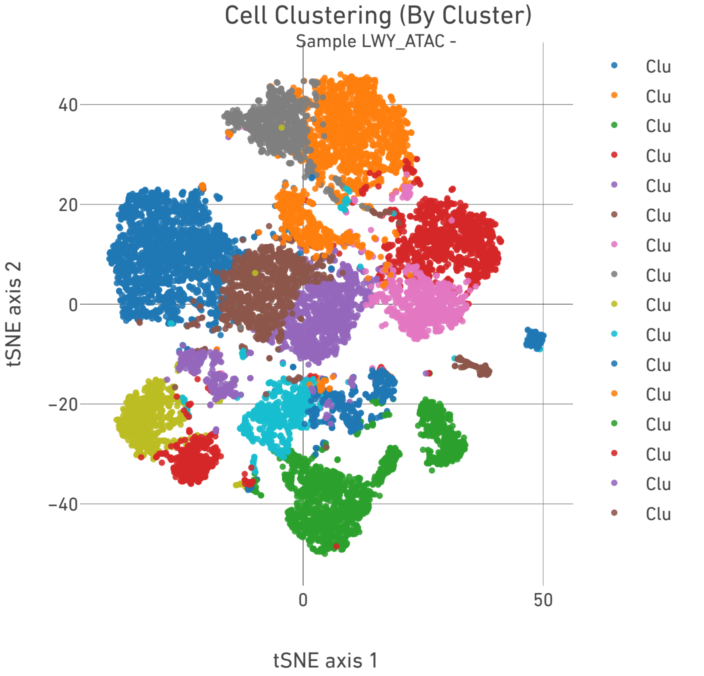

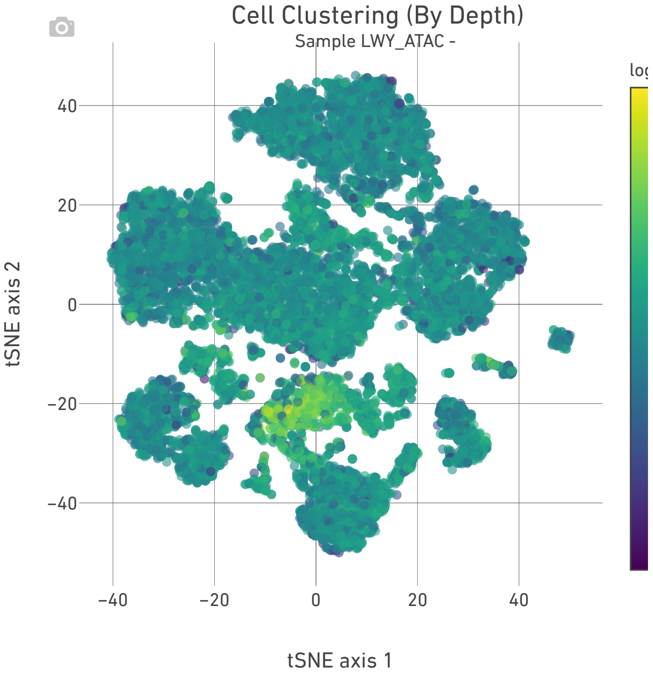

Insert Sizes

Fragments in nucleosome-free regions	40.1%
Fragments flanking a single nucleosome	52.1%

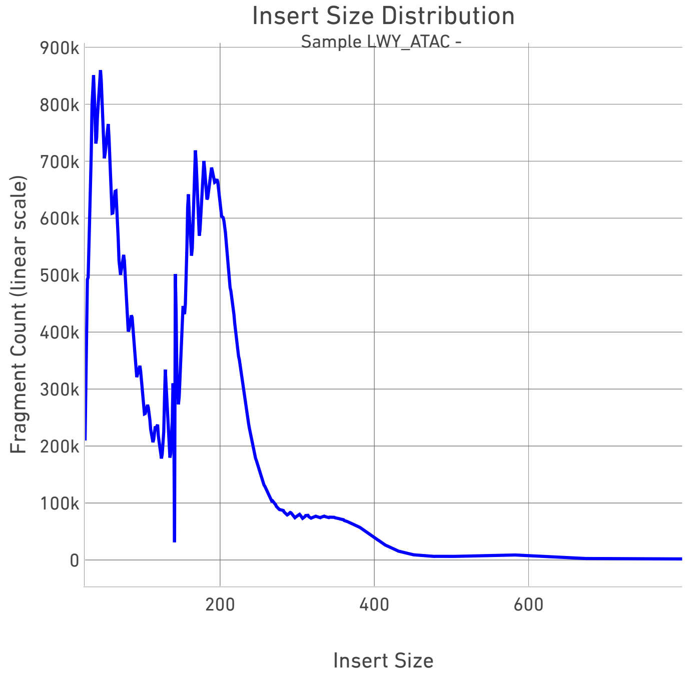

Targeting

Enrichment score of transcription start sites	7.04
Fraction of fragments overlapping TSS	36.5%
Fraction of fragments overlapping called peaks	45.4%
Fraction of transposition events in peaks in cell barcodes	44.0%
Fraction of fragments overlapping any targeted region	66.2%
Fraction of total read pairs mapped confidently to genome (>30 mapq)	87.3%
Fraction of total read pairs that are unmapped and in cell barcodes	0.4%
Fraction of total read pairs in mitochondria and in cell barcodes	0.4%

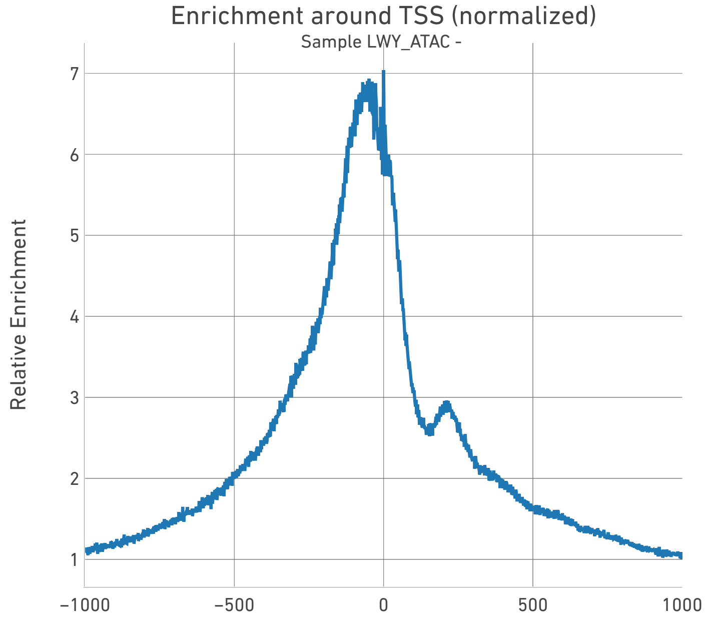

Relative Position (bp from TSS)

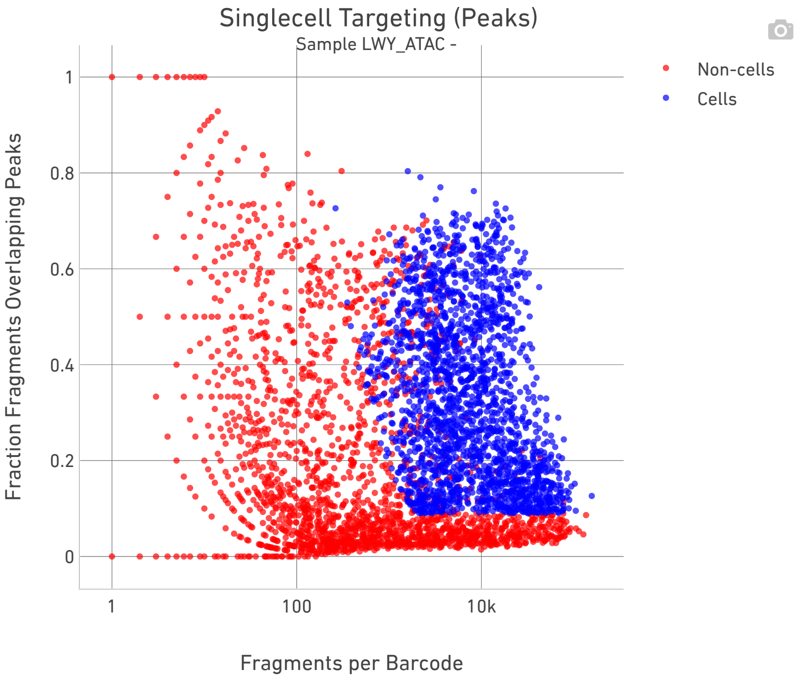

Library Complexity

Percent duplicates	12.5%
Sequencing saturation	29.3%
Estimated bulk library complexity	824,548,746

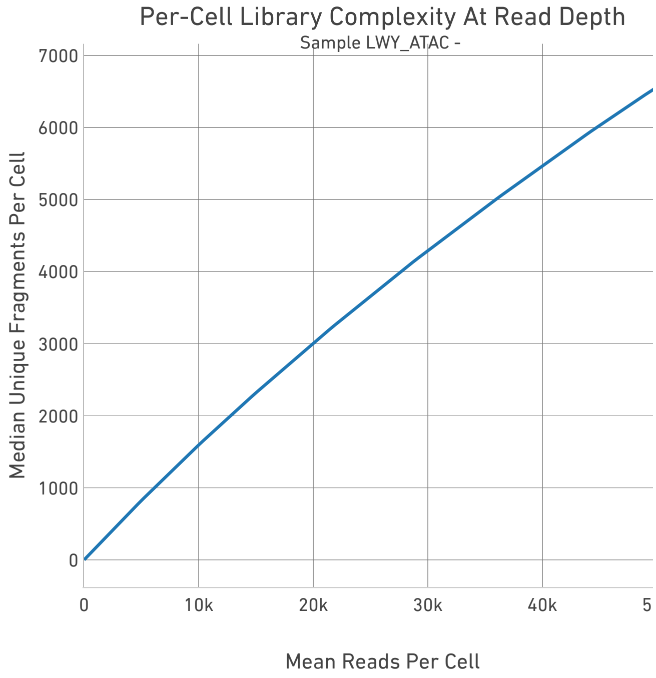

Supplement: Supplementary file 18 [file Data_Sheet_4.PDF]
