# Supplementary material for: Epigenetic Landscapes of Single-Cell Chromatin Accessibility and Transcriptomic Immune Profiles of T Cells in COVID-19 Patients
Source: Front Immunol. 2021 Feb 24;12:625881. doi: 10.3389/fimmu.2021.625881 (PMC7943924; doi:10.3389/fimmu.2021.625881)

10x GENOMICS Cell Ranger ATAC

CCF3_17_ATAC

Alerts

The analysis detected ⚠ 1 warning.

	Alert	Value	Detail
⚠	Too many detected cells	15,481	Estimated number of cells is expected to be under 10,000. A high value might indicate an overloading of cells, a problem during library preparation, or unexpected behavior in the cell calling algorithm.

For guidance, please consult "Interpreting Cell Ranger ATAC Web Summary Files" or contact 10x Genomics Support (support@10xgenomics.com)

15,481
Estimated number of cells

7,548
Median fragments per cell

70.4%
Fraction of fragments overlapping any targeted region

48.3%
Fraction of transposition events in peaks in cell barcodes

Sample

Sample ID	CCF3_17_ATAC
Sample description	
FASTQ path	…00508/ATAC/CCF3_17_ATAC
Pipeline version	1.2.0
Reference path	…abase/ATAC/Homo_sapiens
Organism	Homo_sapiens
Assembly	custom
Annotation	custom

Sequencing

Total number of read pairs	318,410,692
Fraction of read pairs with a valid barcode	98.4%
Q30 bases in Read 1	94.0%
Q30 bases in Read 2	93.9%
Q30 bases in Barcode	92.5%
Q30 bases in Sample Index	93.3%

Cells

Estimated number of cells	15,481
Lower threshold on the number of fragments overlapping peaks per barcode to annotate barcode as cell	252.00
Median fragments per cell	7,548
Median fragments per non-cell barcode	2

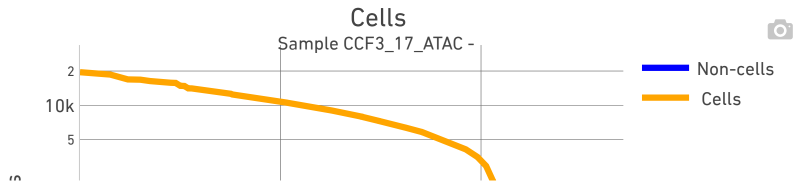

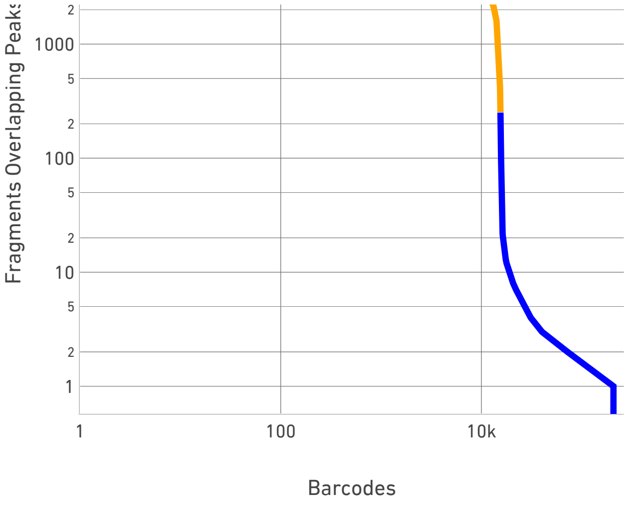

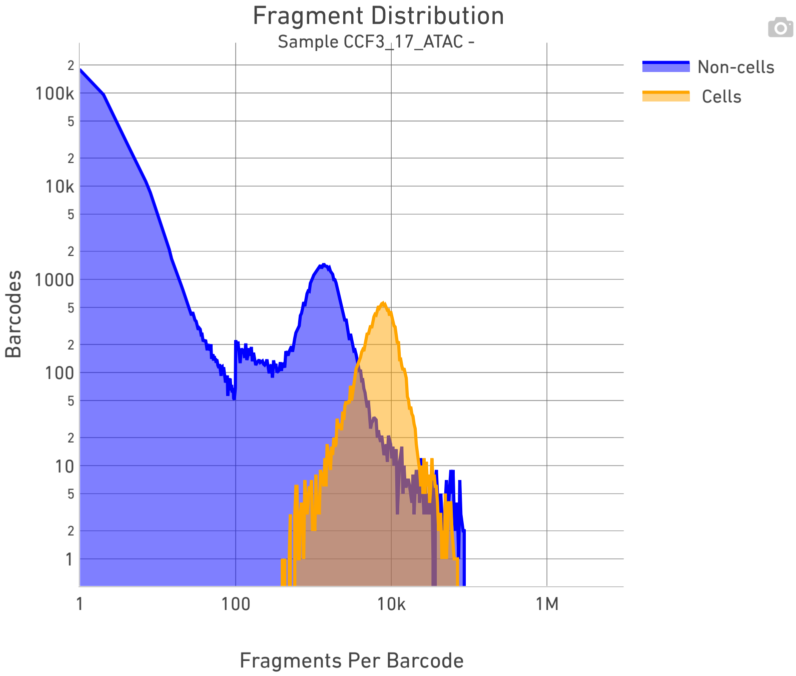

Cell Clustering

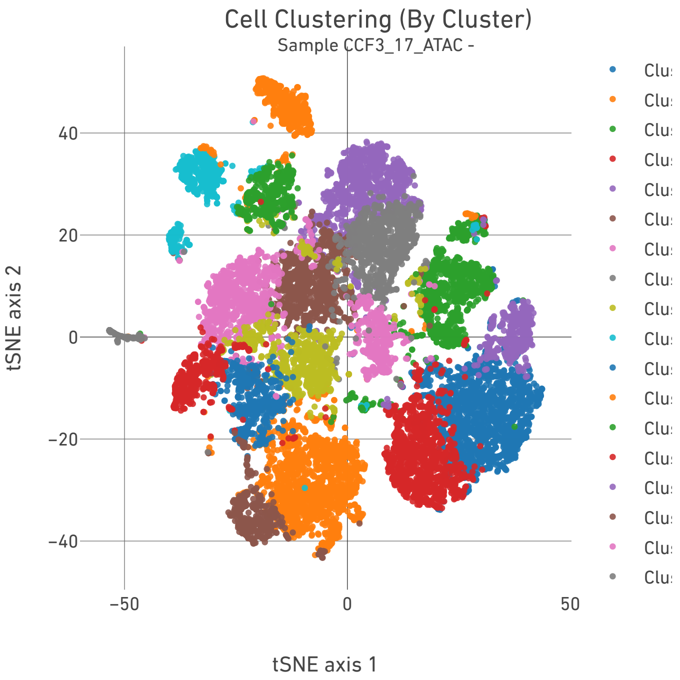

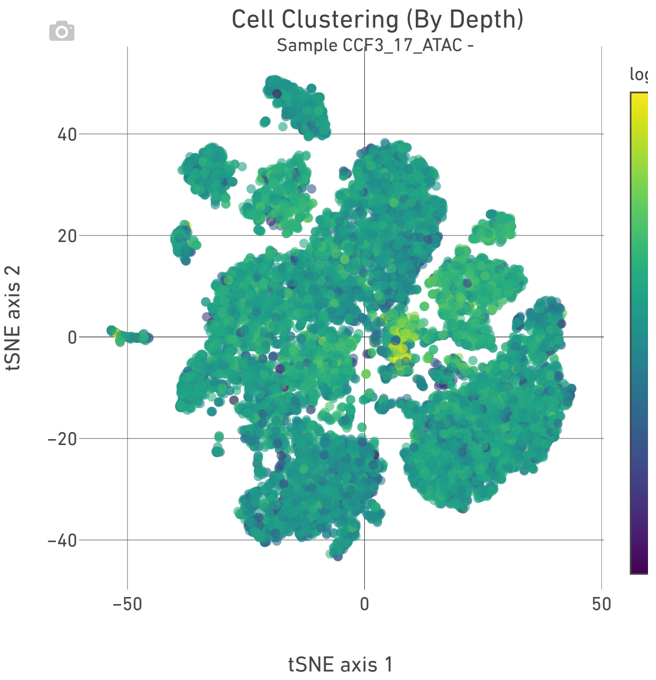

Insert Sizes

Fragments in nucleosome-free regions	35.2%
Fragments flanking a single nucleosome	53.6%

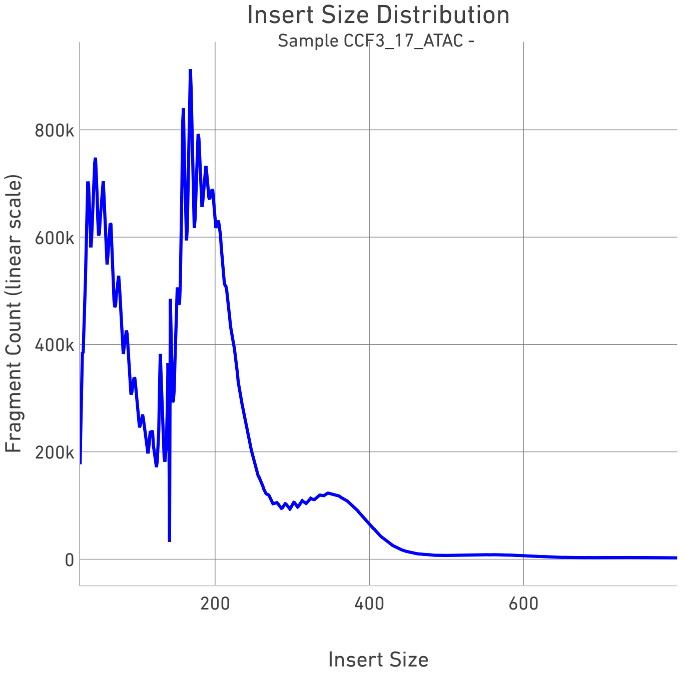

Targeting

Enrichment score of transcription start sites	6.67
Fraction of fragments overlapping TSS	38.6%
Fraction of fragments overlapping called peaks	50.2%
Fraction of transposition events in peaks in cell barcodes	48.3%
Fraction of fragments overlapping any targeted region	70.4%
Fraction of total read pairs mapped confidently to genome (>30 mapq)	88.4%
Fraction of total read pairs that are unmapped and in cell barcodes	0.4%
Fraction of total read pairs in mitochondria and in cell barcodes	0.4%

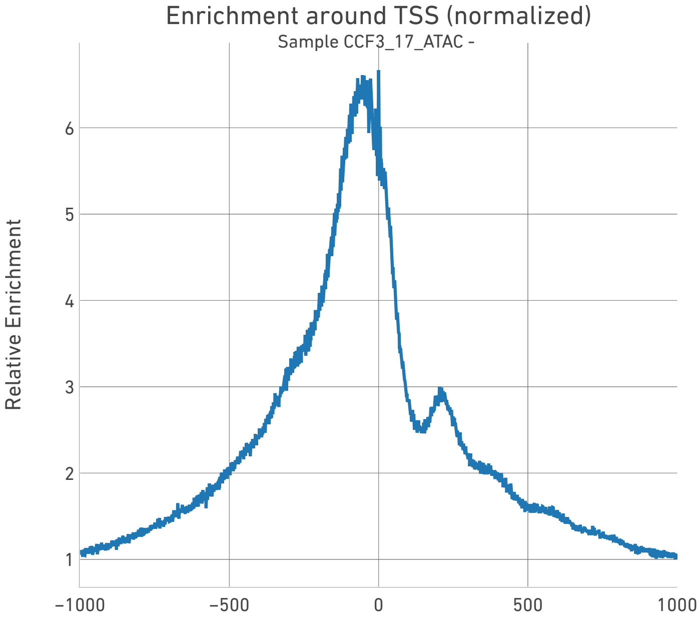

Relative Position (bp from TSS)

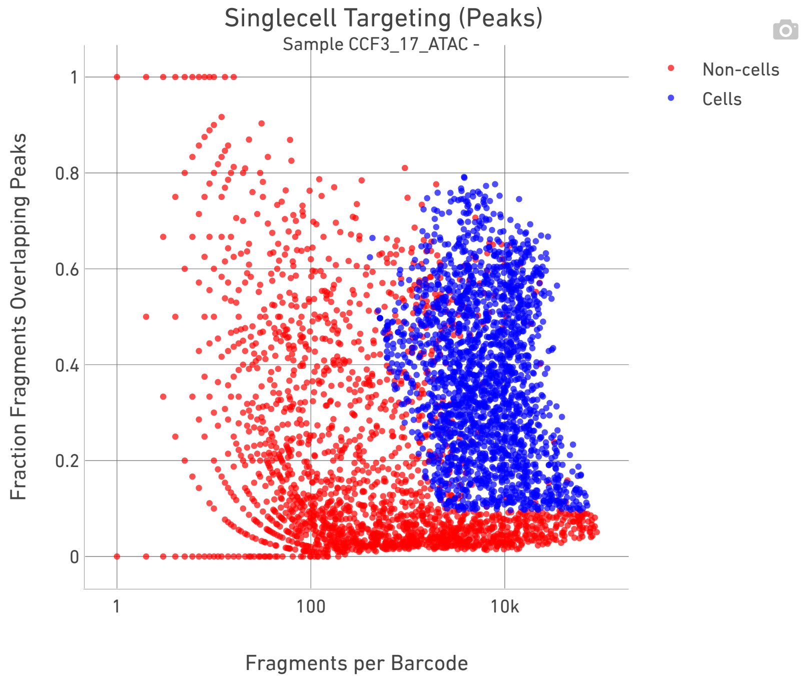

Library Complexity

Percent duplicates	14.1%
Sequencing saturation	28.4%
Estimated bulk library complexity	746,032,576

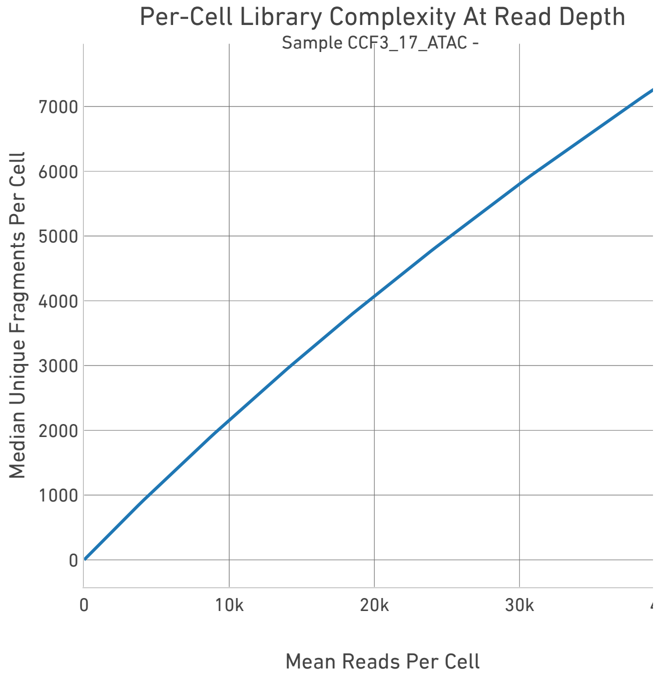

Supplement: Supplementary file 19 [file Data_Sheet_5.PDF]
