# Supplementary material for: Epigenetic Landscapes of Single-Cell Chromatin Accessibility and Transcriptomic Immune Profiles of T Cells in COVID-19 Patients
Source: Front Immunol. 2021 Feb 24;12:625881. doi: 10.3389/fimmu.2021.625881 (PMC7943924; doi:10.3389/fimmu.2021.625881)

10x GENOMICS Cell Ranger ATAC

WH313_ATAC

Alerts

The analysis detected ⚠ 1 warning.

	Alert	Value	Detail
⚠	Too many detected cells	10,396	Estimated number of cells is expected to be under 10,000. A high value might indicate an overloading of cells, a problem during library preparation, or unexpected behavior in the cell calling algorithm.

For guidance, please consult "Interpreting Cell Ranger ATAC Web Summary Files" or contact 10x Genomics Support (support@10xgenomics.com)

10,396
Estimated number of cells

6,506
Median fragments per cell

78.8%
Fraction of fragments overlapping any targeted region

61.0%
Fraction of transposition events in peaks in cell barcodes

Sample

Sample ID	WH313_ATAC
Sample description	
FASTQ path	…0200508/ATAC/WH313_ATAC
Pipeline version	1.2.0
Reference path	…abase/ATAC/Homo_sapiens
Organism	Homo_sapiens
Assembly	custom
Annotation	custom

Sequencing ⓘ

Total number of read pairs	478,917,650
Fraction of read pairs with a valid barcode	98.2%
Q30 bases in Read 1	92.8%
Q30 bases in Read 2	92.9%
Q30 bases in Barcode	90.7%
Q30 bases in Sample Index	92.3%

Cells ⓘ

Estimated number of cells	10,396
Lower threshold on the number of fragments overlapping peaks per barcode to annotate barcode as cell	2,144.00
Median fragments per cell	6,506
Median fragments per non-cell barcode	1

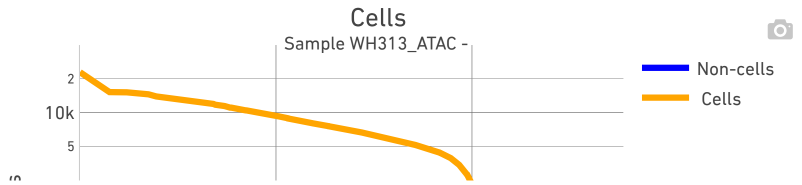

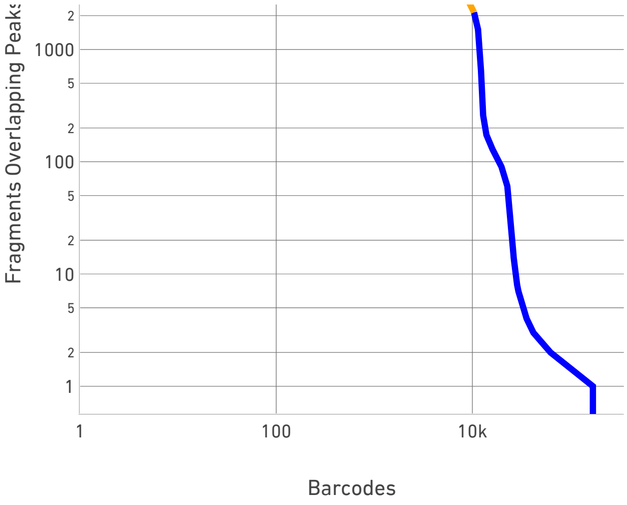

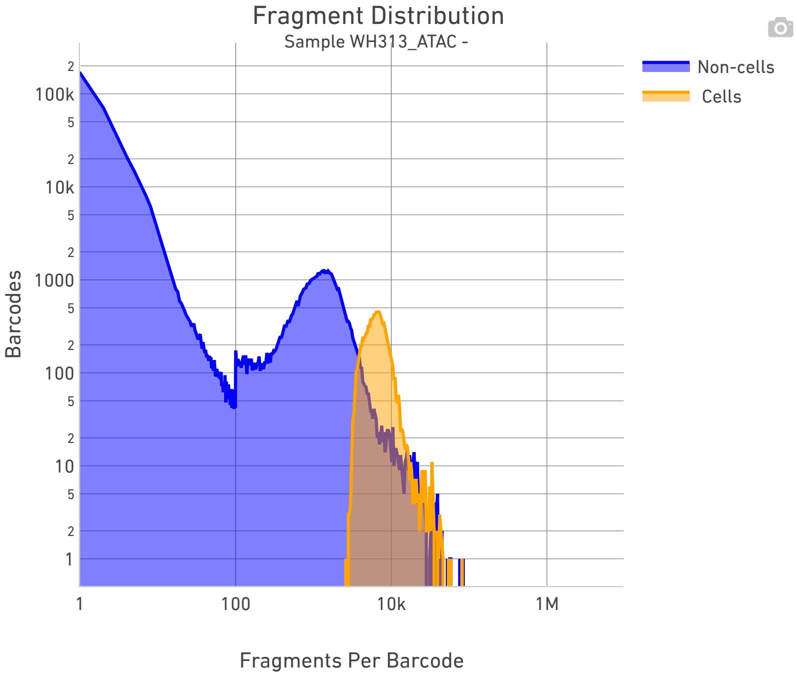

Cell Clustering

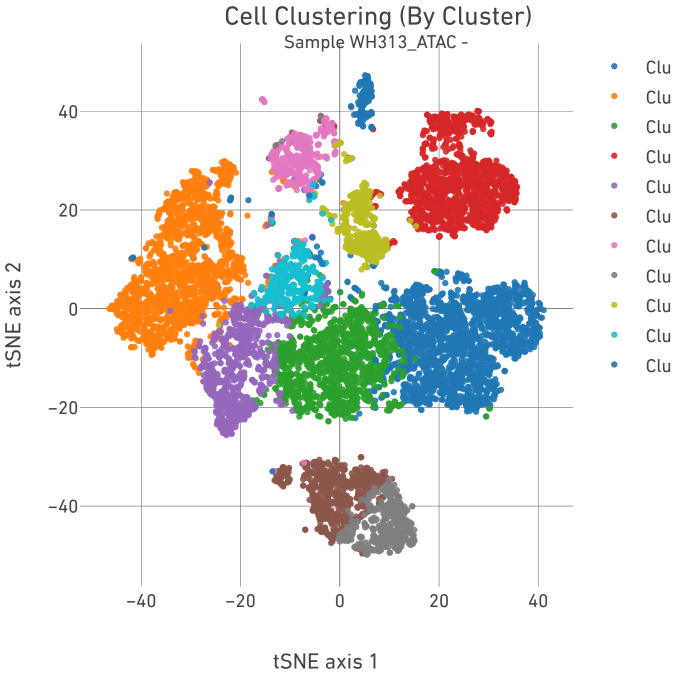

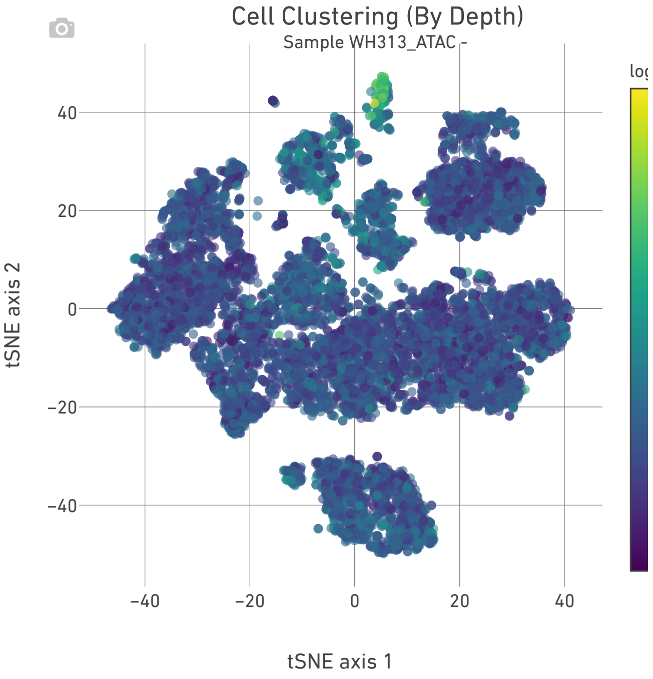

Insert Sizes

Fragments in nucleosome-free regions	54.1%
Fragments flanking a single nucleosome	43.7%

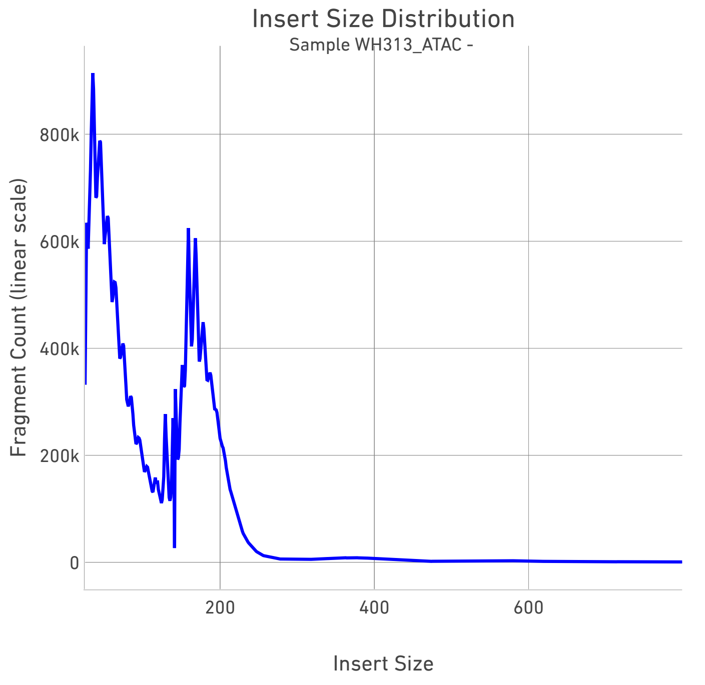

Targeting

Enrichment score of transcription start sites	9.52
Fraction of fragments overlapping TSS	53.4%
Fraction of fragments overlapping called peaks	62.1%
Fraction of transposition events in peaks in cell barcodes	61.0%
Fraction of fragments overlapping any targeted region	78.8%
Fraction of total read pairs mapped confidently to genome (>30 mapq)	83.9%
Fraction of total read pairs that are unmapped and in cell barcodes	0.3%
Fraction of total read pairs in mitochondria and in cell barcodes	0.2%

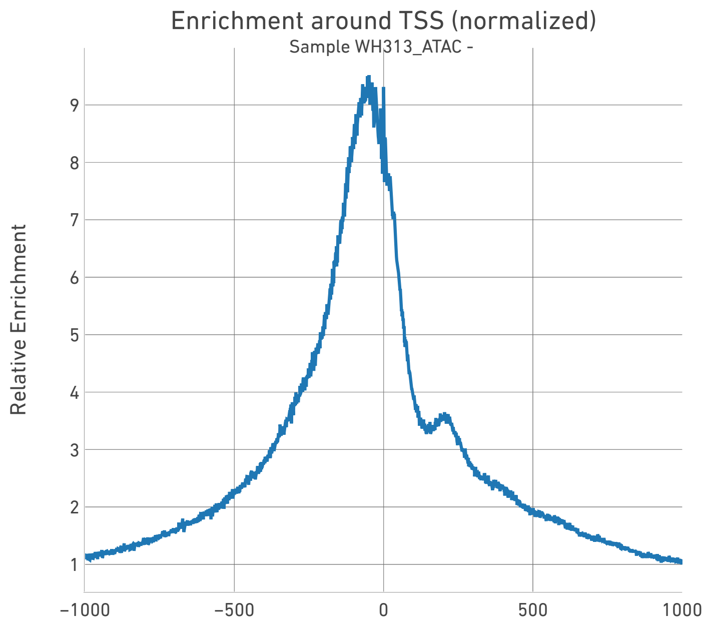

Relative Position (bp from TSS)

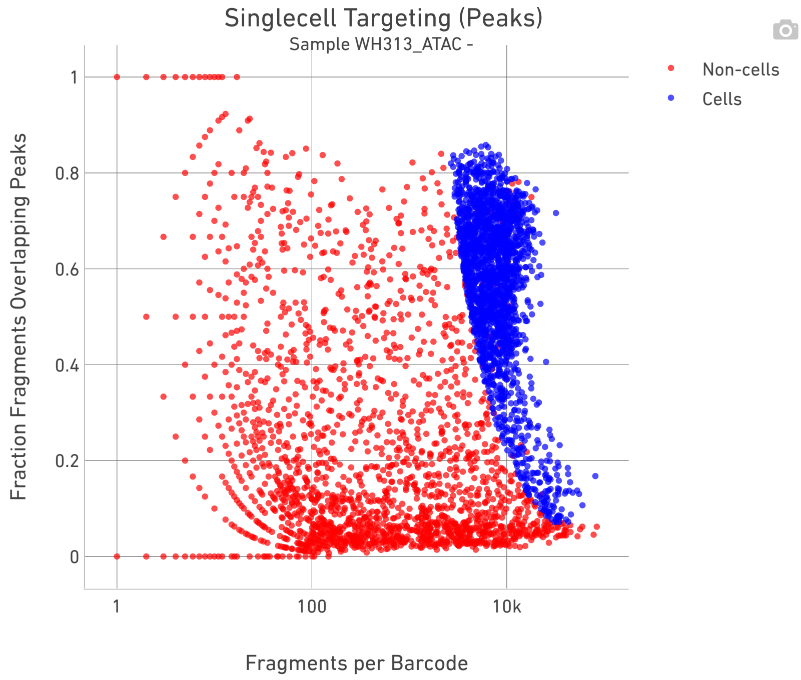

Library Complexity

Percent duplicates	16.8%
Sequencing saturation	63.6%
Estimated bulk library complexity	249,045,695

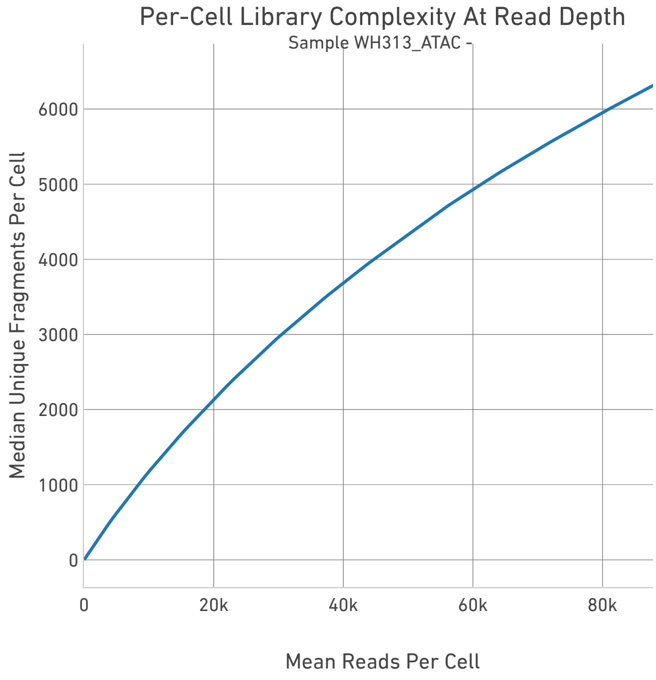

Supplement: Supplementary file 20 [file Data_Sheet_6.PDF]
