# Supplementary material for: Epigenetic Landscapes of Single-Cell Chromatin Accessibility and Transcriptomic Immune Profiles of T Cells in COVID-19 Patients
Source: Front Immunol. 2021 Feb 24;12:625881. doi: 10.3389/fimmu.2021.625881 (PMC7943924; doi:10.3389/fimmu.2021.625881)

Cell Ranger ATAC

ZXH313_ATAC

For guidance, please consult "Interpreting Cell Ranger ATAC Web Summary Files" or contact 10x Genomics Support (support@10xgenomics.com)

8,810
Estimated number of cells

6,145
Median fragments per cell

57.5%
Fraction of fragments overlapping any targeted region

31.4%
Fraction of transposition events in peaks in cell barcodes

Sample

Sample ID	ZXH313_ATAC
Sample description	
FASTQ path	…200508/ATAC/ZXH313_ATAC
Pipeline version	1.2.0
Reference path	…abase/ATAC/Homo_sapiens
Organism	Homo_sapiens
Assembly	custom
Annotation	custom

Sequencing

Total number of read pairs	251,373,469
Fraction of read pairs with a valid barcode	98.1%
Q30 bases in Read 1	91.9%
Q30 bases in Read 2	91.9%
Q30 bases in Barcode	90.0%
Q30 bases in Sample Index	92.1%

Cells

Estimated number of cells	8,810
Lower threshold on the number of fragments overlapping peaks per barcode to annotate barcode as cell	214.00
Median fragments per cell	6,145
Median fragments per non-cell barcode	2

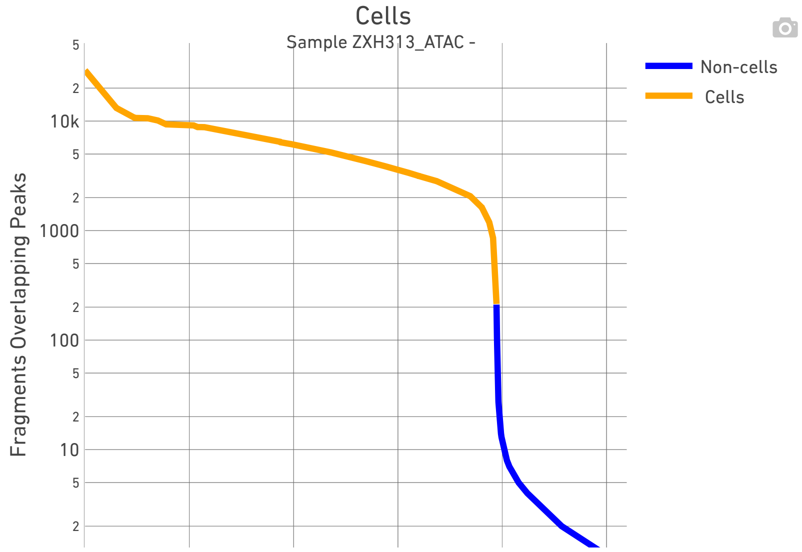

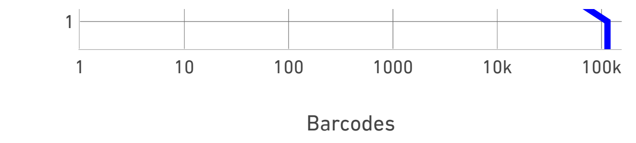

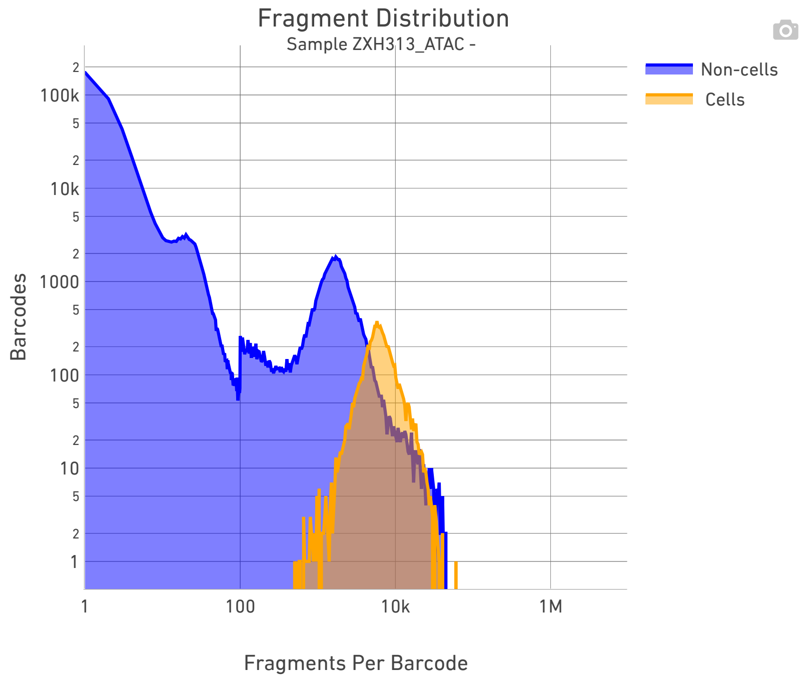

Cell Clustering

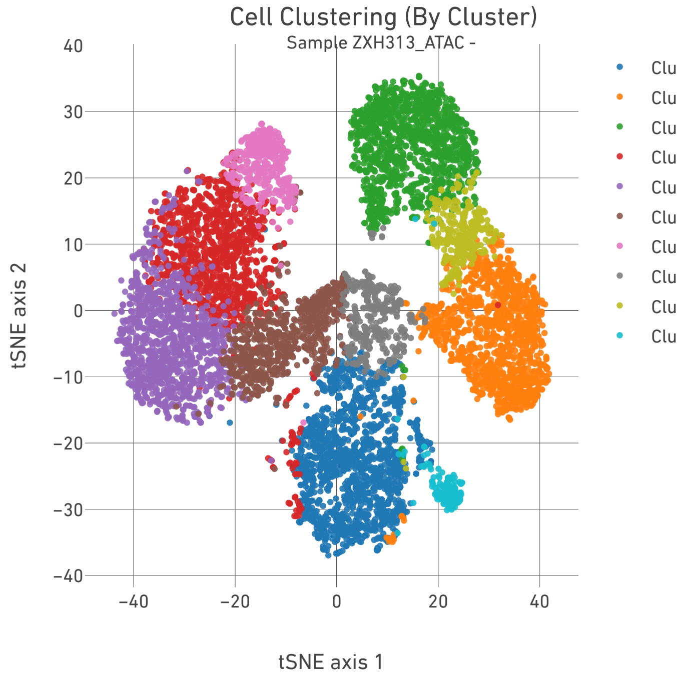

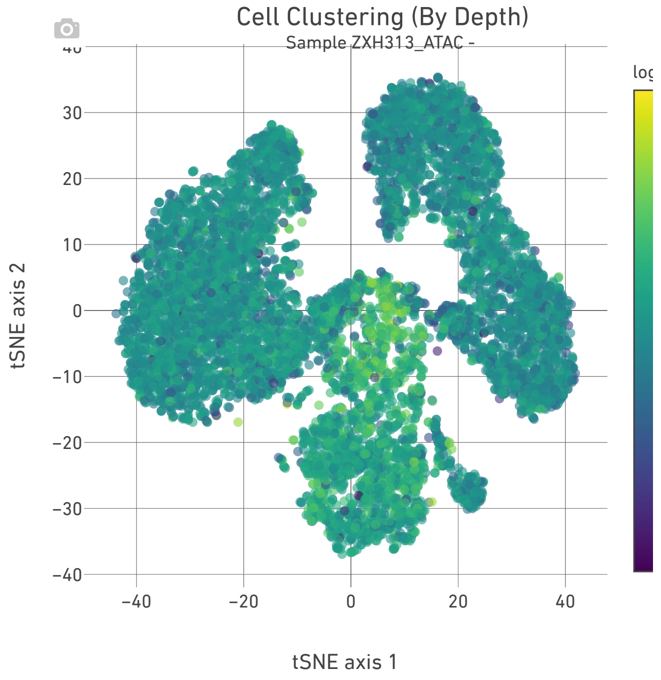

Insert Sizes

Fragments in nucleosome-free regions	31.4%
Fragments flanking a single nucleosome	52.9%

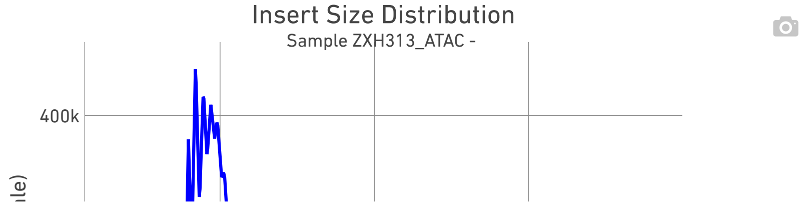

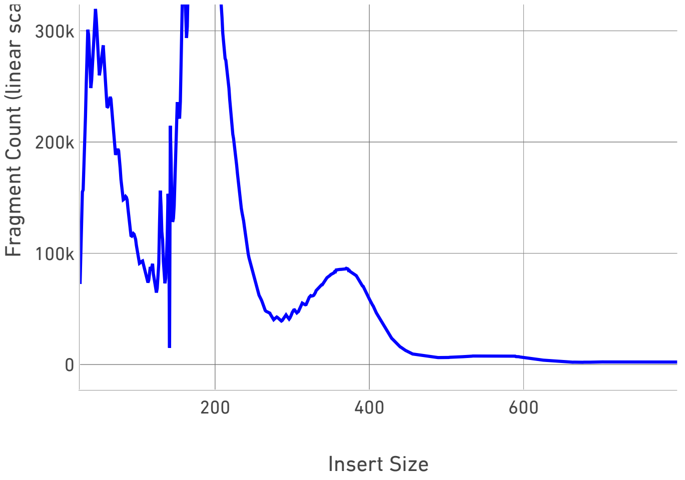

Targeting

Enrichment score of transcription start sites	4.27
Fraction of fragments overlapping TSS	27.0%
Fraction of fragments overlapping called peaks	33.2%
Fraction of transposition events in peaks in cell barcodes	31.4%
Fraction of fragments overlapping any targeted region	57.5%
Fraction of total read pairs mapped confidently to genome (>30 mapq)	87.8%
Fraction of total read pairs that are unmapped and in cell barcodes	0.2%
Fraction of total read pairs in mitochondria and in cell barcodes	0.2%

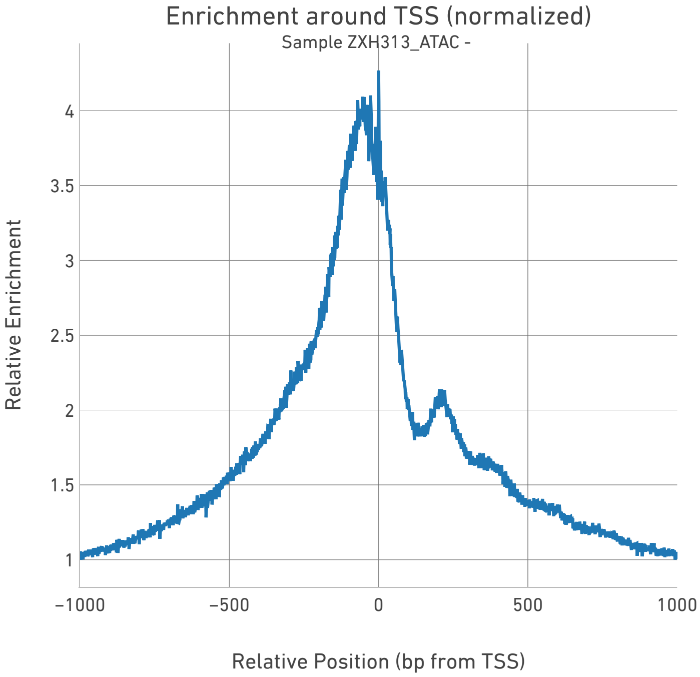

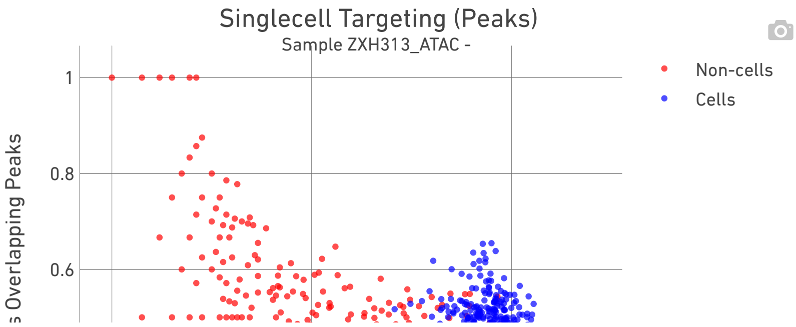

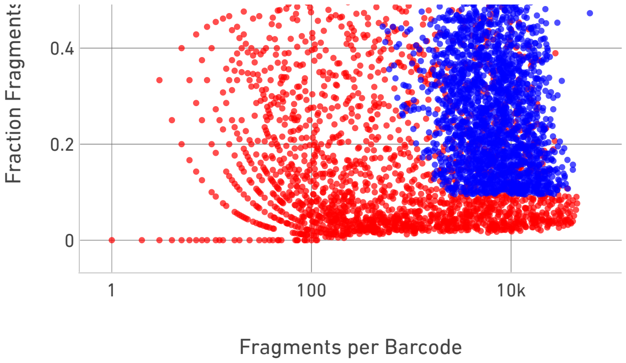

Library Complexity

Percent duplicates	5.2%
Sequencing saturation	17.9%
Estimated bulk library complexity	1,001,289,954

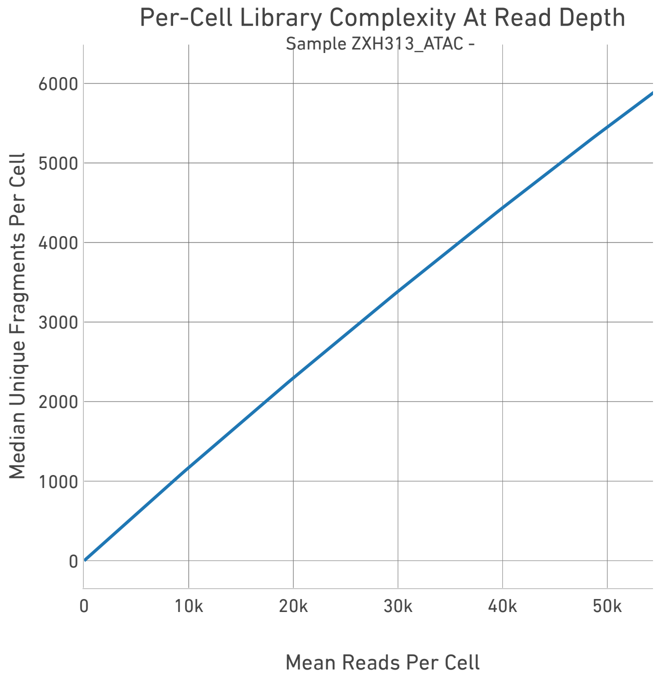

Supplement: Supplementary file 22 [file Data_Sheet_8.PDF]
