# Supplementary material for: Epigenetic Landscapes of Single-Cell Chromatin Accessibility and Transcriptomic Immune Profiles of T Cells in COVID-19 Patients
Source: Front Immunol. 2021 Feb 24;12:625881. doi: 10.3389/fimmu.2021.625881 (PMC7943924; doi:10.3389/fimmu.2021.625881)

10x GENOMICS Cell Ranger ATAC

HSZ417_ATAC

Alerts

The analysis detected ⚠ 2 warnings.

	Alert	Value	Detail
⚠	Fraction of fragments in targeted regions is low	53.0%	The fraction of fragments (that passed all filters) overlapping targeted regions is expected to be above 55%. Note that this number may be much lower for custom references that do not include targeting files related to enhancers and other functional domains.
⚠	The percentage of transposition events falling within peaks is low	20.9%	It is expected that more than 25% of the transposition events fall within peak regions. A lower value could suggest peak undercalling or low sequencing depth.

For guidance, please consult "Interpreting Cell Ranger ATAC Web Summary Files" or contact 10x Genomics Support (support@10xgenomics.com)

3,670
Estimated number of cells

7,800
Median fragments per cell

53.0%
Fraction of fragments overlapping any targeted region

20.9%
Fraction of transposition events in peaks in cell barcodes

Sample

Sample ID	HSZ417_ATAC
Sample description	
FASTQ path	…200508/ATAC/HSZ417_ATAC
Pipeline version	1.2.0
Reference path	…abase/ATAC/Homo_sapiens
Organism	Homo_sapiens
Assembly	custom
Annotation	custom

Sequencing

Total number of read pairs	372,986,576
Fraction of read pairs with a valid barcode	97.8%
Q30 bases in Read 1	91.7%
Q30 bases in Read 2	91.6%
Q30 bases in Barcode	89.1%
Q30 bases in Sample Index	91.2%

Cells

Estimated number of cells	3,670
Lower threshold on the number of fragments overlapping peaks per barcode to annotate barcode as cell	248.00
Median fragments per cell	7,800
Median fragments per non-cell barcode	2

Cells

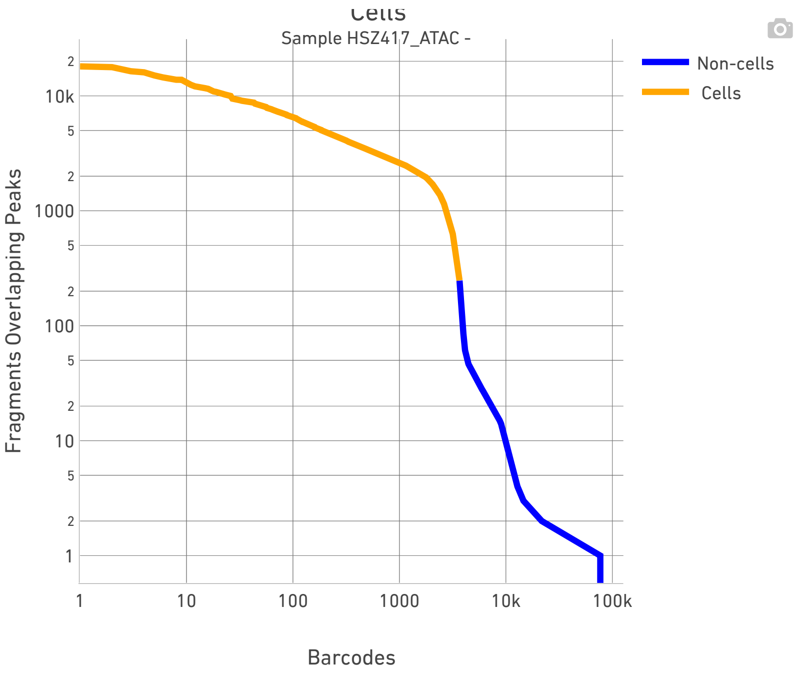

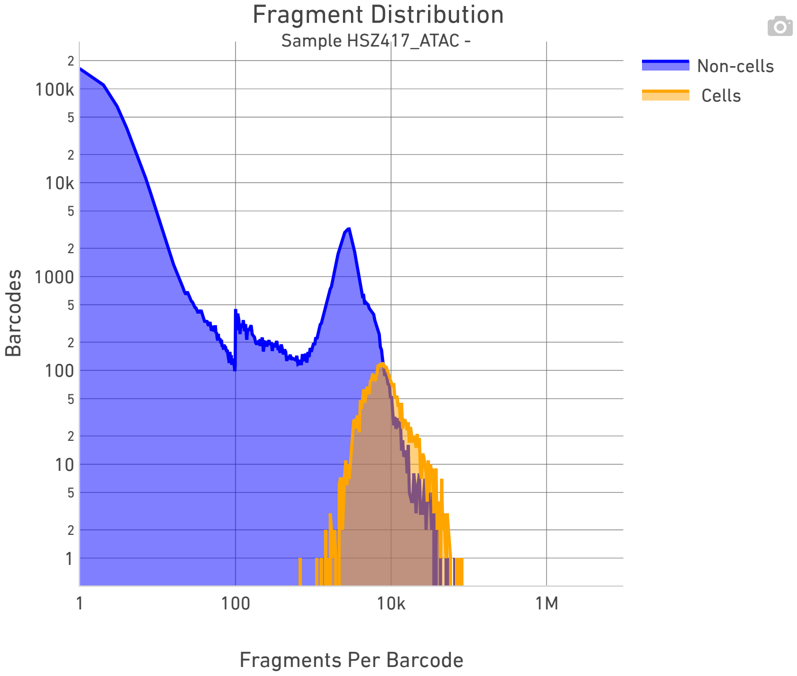

Cell Clustering

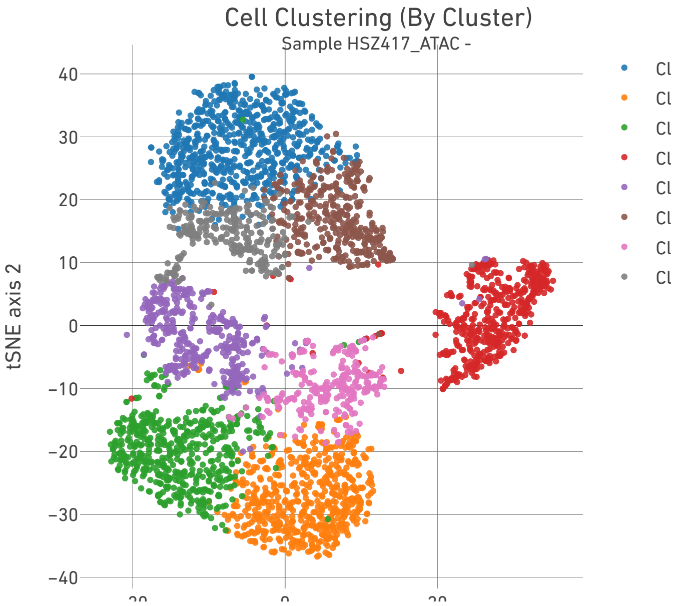

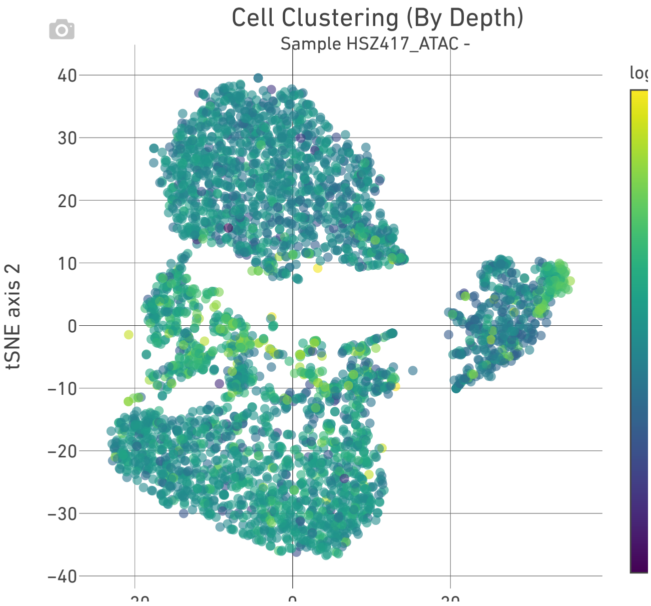

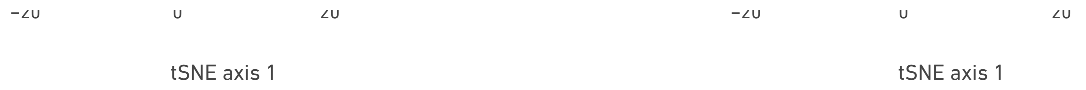

Insert Sizes

Fragments in nucleosome-free regions	27.3%
Fragments flanking a single nucleosome	64.0%

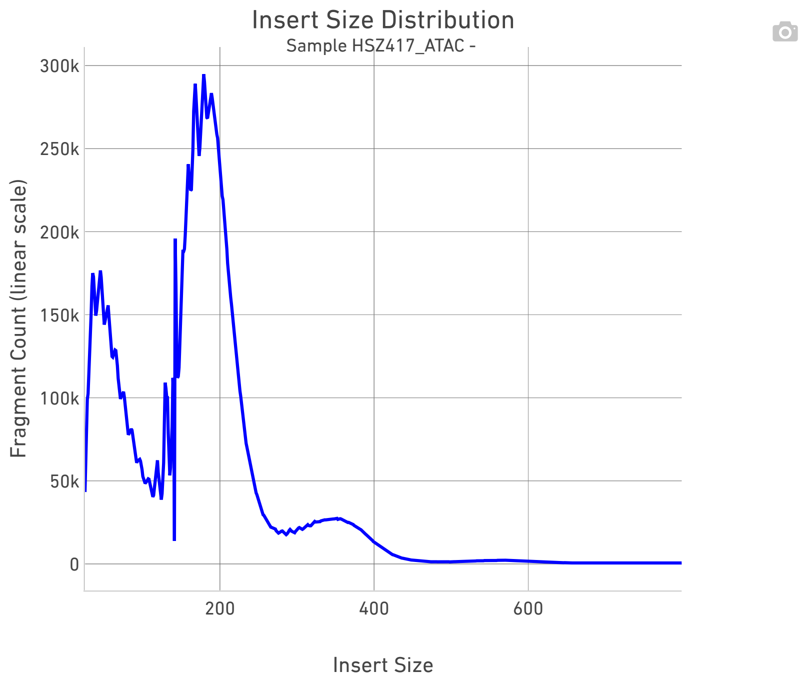

Targeting

Enrichment score of transcription start sites	2.75
Fraction of fragments overlapping TSS	23.8%
Fraction of fragments overlapping called peaks	22.4%
Fraction of transposition events in peaks in cell barcodes	20.9%
Fraction of fragments overlapping any targeted region	53.0%

Fraction of total read pairs mapped confidently to genome (>30 mapq)	85.8%
Fraction of total read pairs that are unmapped and in cell barcodes	0.1%
Fraction of total read pairs in mitochondria and in cell barcodes	0.1%

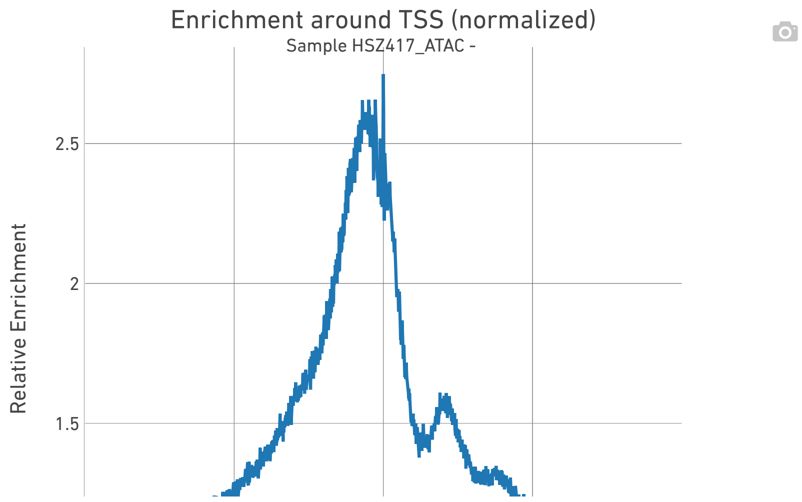

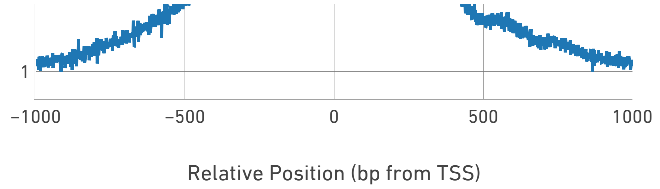

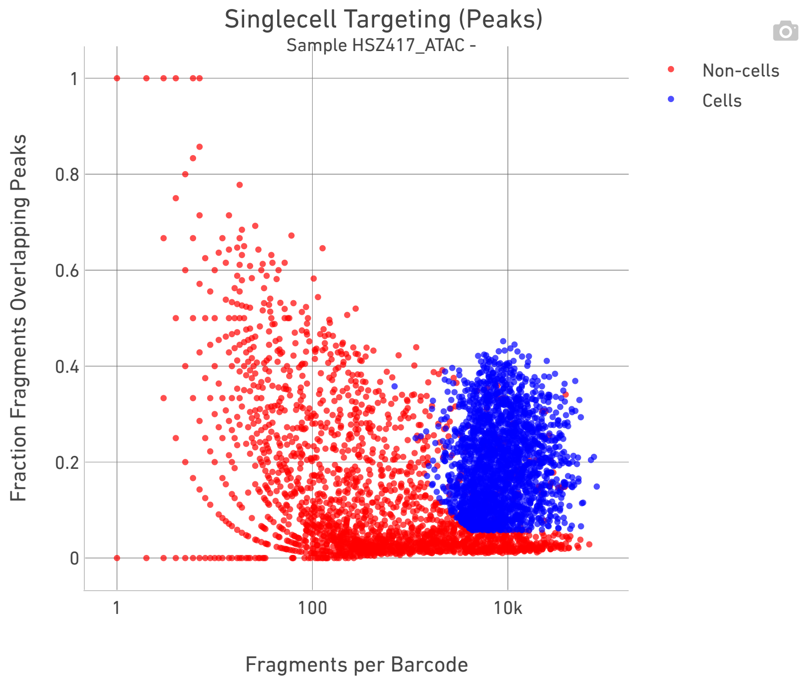

Library Complexity ⓘ

Percent duplicates	2.5%
Sequencing saturation	23.0%
Estimated bulk library complexity	1,110,838,297

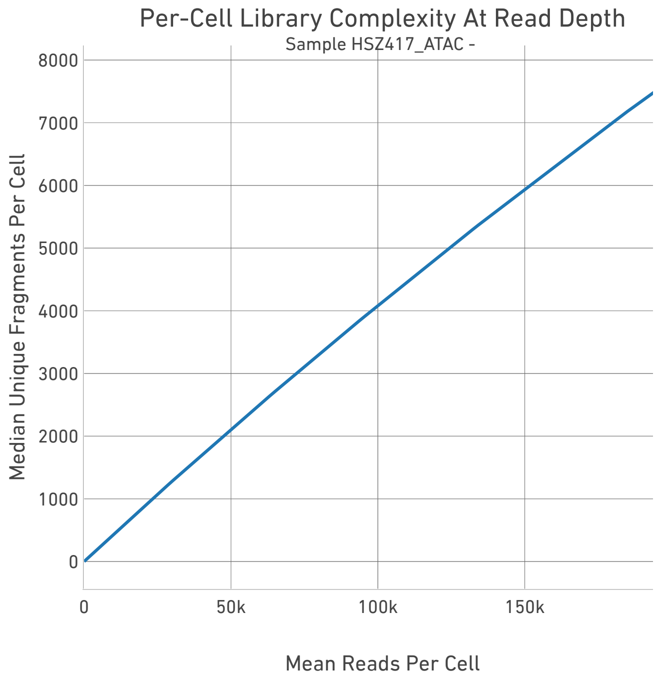

Supplement: Supplementary file 23 [file Data_Sheet_9.PDF]
