# Supplementary material for: Epigenetic Landscapes of Single-Cell Chromatin Accessibility and Transcriptomic Immune Profiles of T Cells in COVID-19 Patients
Source: Front Immunol. 2021 Feb 24;12:625881. doi: 10.3389/fimmu.2021.625881 (PMC7943924; doi:10.3389/fimmu.2021.625881)

10x GENOMICS Cell Ranger ATAC

CSX320_ATAC

Alerts

The analysis detected ⚠ 2 warnings.

	Alert	Value	Detail
⚠	Fraction of fragments in targeted regions is low	54.2%	The fraction of fragments (that passed all filters) overlapping targeted regions is expected to be above 55%. Note that this number may be much lower for custom references that do not include targeting files related to enhancers and other functional domains.
⚠	The percentage of transposition events falling within peaks is low	25.0%	It is expected that more than 25% of the transposition events fall within peak regions. A lower value could suggest peak undercalling or low sequencing depth.

For guidance, please consult "Interpreting Cell Ranger ATAC Web Summary Files" or contact 10x Genomics Support (support@10xgenomics.com)

8,219
Estimated number of cells

6,447
Median fragments per cell

54.2%
Fraction of fragments overlapping any targeted region

25.0%
Fraction of transposition events in peaks in cell barcodes

Sample

Sample ID	CSX320_ATAC
Sample description	
FASTQ path	…200508/ATAC/CSX320_ATAC
Pipeline version	1.2.0
Reference path	…abase/ATAC/Homo_sapiens
Organism	Homo_sapiens
Assembly	custom
Annotation	custom

Sequencing ⓘ

Total number of read pairs	421,780,857
Fraction of read pairs with a valid barcode	90.7%
Q30 bases in Read 1	91.5%
Q30 bases in Read 2	91.4%
Q30 bases in Barcode	90.0%
Q30 bases in Sample Index	90.9%

Cells ⓘ

Estimated number of cells	8,219
Lower threshold on the number of fragments overlapping peaks per barcode to annotate barcode as cell	274.00
Median fragments per cell	6,447
Median fragments per non-cell barcode	2

Cells

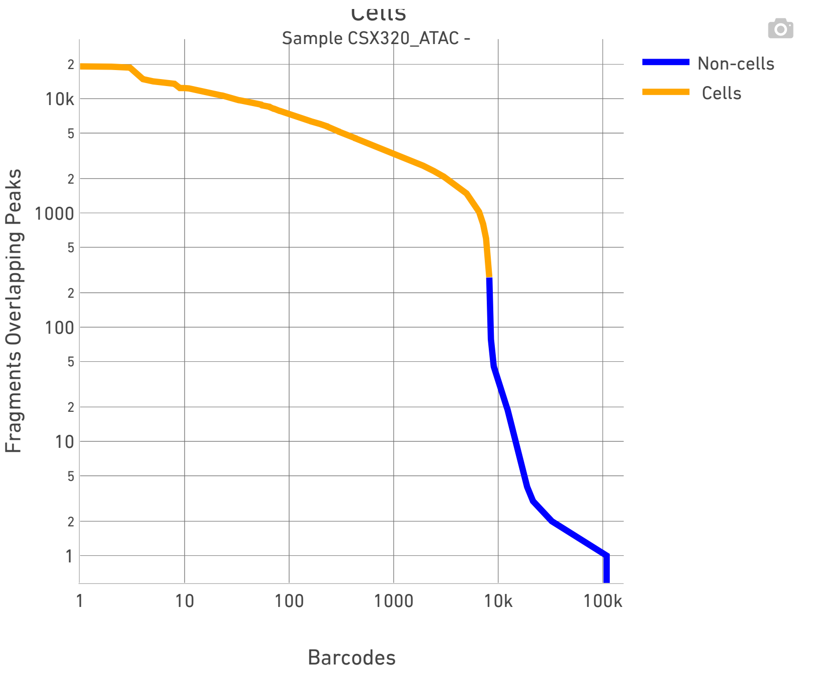

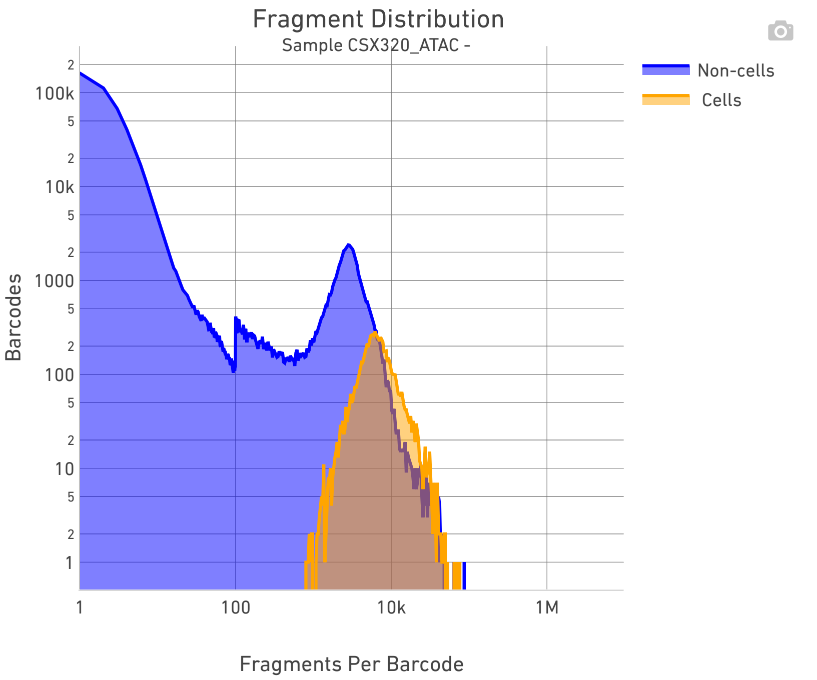

Cell Clustering

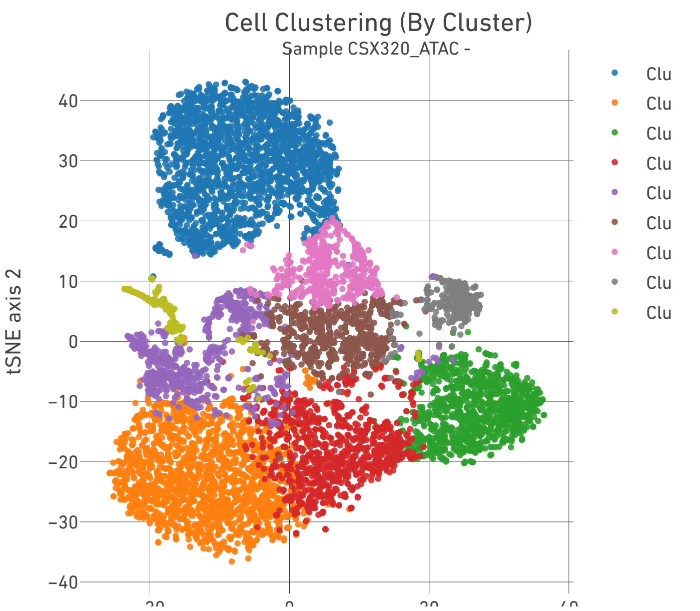

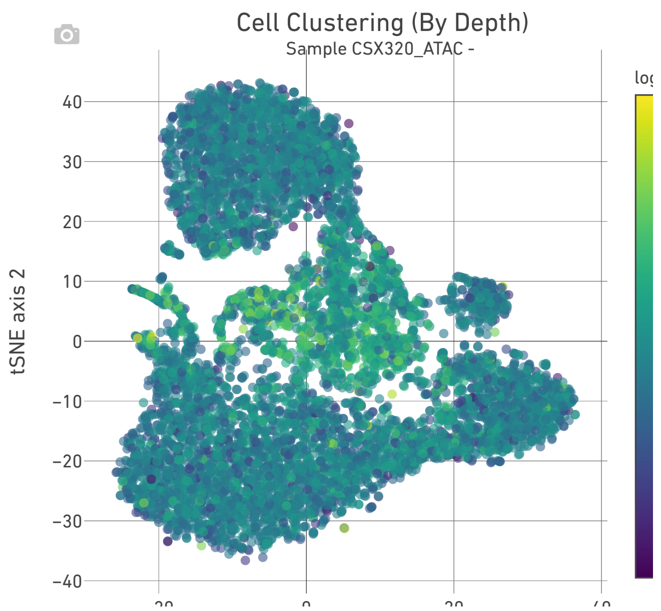

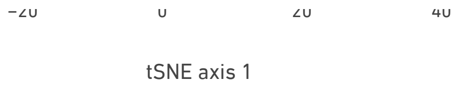

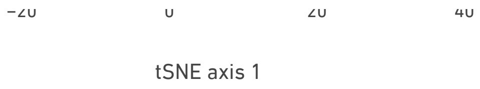

Insert Sizes

Fragments in nucleosome-free regions	40.2%
Fragments flanking a single nucleosome	52.2%

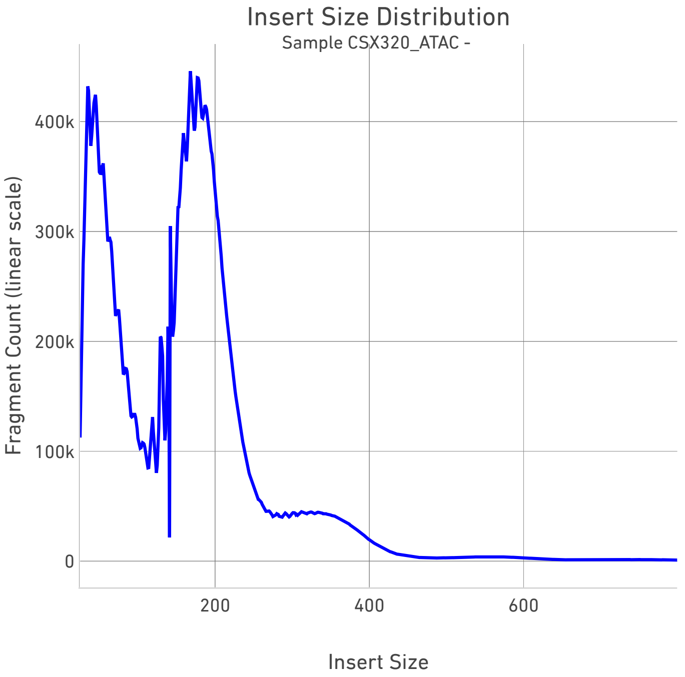

Targeting

Enrichment score of transcription start sites	3.62
Fraction of fragments overlapping TSS	25.2%
Fraction of fragments overlapping called peaks	26.4%
Fraction of transposition events in peaks in cell barcodes	25.0%
Fraction of fragments overlapping any targeted region	54.2%

Fraction of total read pairs mapped confidently to genome (>30 mapq)	78.1%
Fraction of total read pairs that are unmapped and in cell barcodes	0.2%
Fraction of total read pairs in mitochondria and in cell barcodes	0.1%

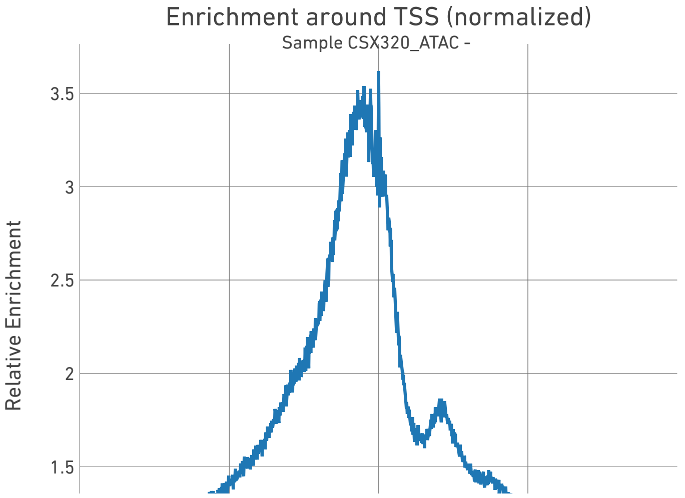

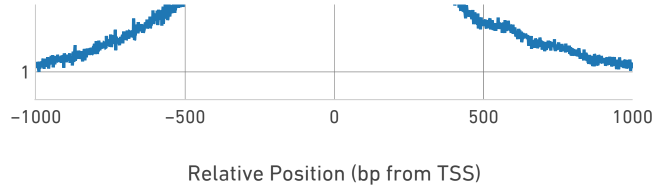

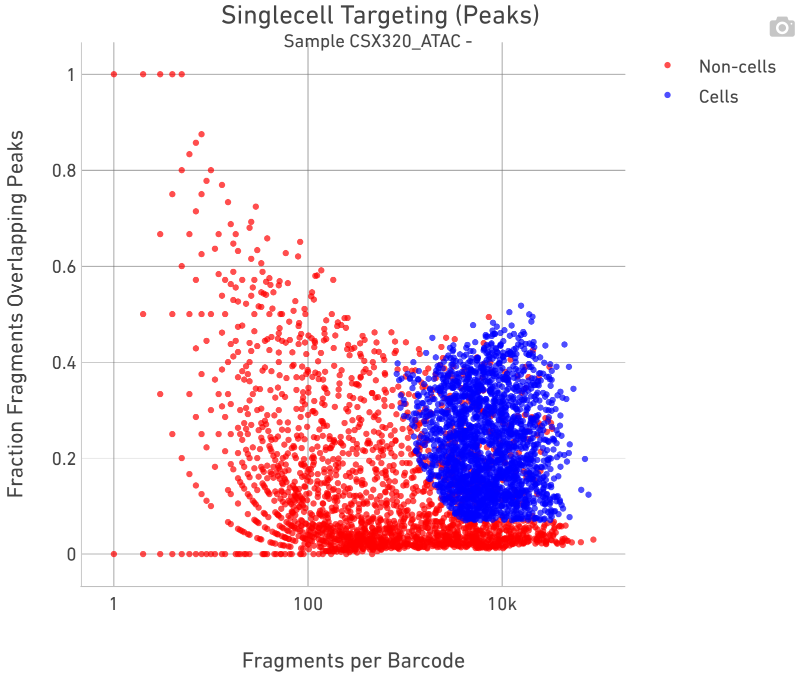

Library Complexity

Percent duplicates	3.7%
Sequencing saturation	20.9%
Estimated bulk library complexity	1,270,286,513

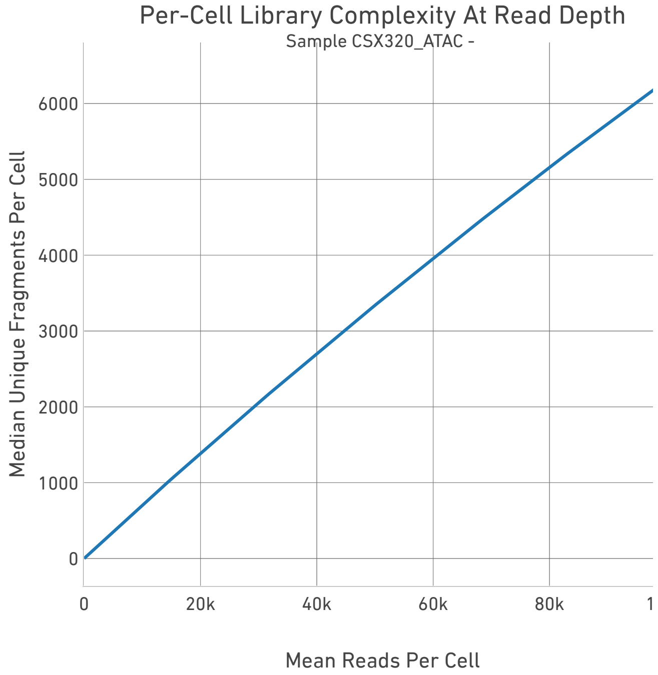

Supplement: Supplementary file 24 [file Data_Sheet_10.PDF]
